# Pseudo‐Ring Methodology to Control the Conformations of Macrocyclic Peptides

**DOI:** 10.1002/chem.202500581

**Published:** 2025-03-27

**Authors:** Matthew Diamandas, Yang (Daniel) Ou, Ben Zhen Huang, Andrei K. Yudin

**Affiliations:** ^1^ Davenport Research Laboratories University of Toronto 80 St. George St Toronto Ontario M5S 3H6 Canada

**Keywords:** conformation, fluorescence, heterocycles, macrocycles, peptides, pseudo‐rings

## Abstract

A pseudo‐ring is a medium‐sized cyclic structure, held together by a strong hydrogen bond, that can act as a mimetic of a covalently linked heterocycle. Molecules that contain pseudo‐rings can switch between “open” and “closed” forms. This paper examines the utility of pseudo‐ring constructs to control conformations and properties of macrocycles. The spectroscopic evidence suggests that the “open” and “closed” pseudo‐ring‐containing macrocycles differ in their three‐dimensional characteristics, which results in markedly different lipophilicities. The dynamic nature of pseudo‐rings has led to the emergence of tunable fluorescence that should be useful in environment‐sensitive applications.

## Graphical abstract

Pseudo‐ring grafts represent a powerful conformational control tool in macrocyclic peptides. “Open” and “closed” conformations can be accessed from a single building block which allow for macrocyclic peptides with unique conformations, lipophilicities and fluorescence.

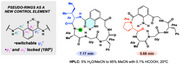

## 1 Introduction

**Figure 1:**
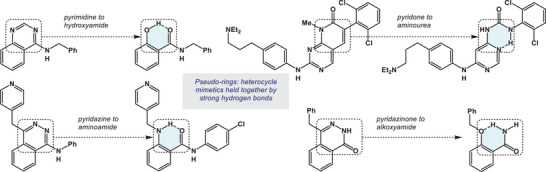
6‐Membered pseudo‐rings that serve as heterocycle mimics in small molecule bioactive compounds.

The dynamic conformational behavior of peptide macrocycles determines their properties. We and others have been intrigued by the role of heterocycles during site‐specific amino acid replacement.^[^ ^1^, ^2^, ^3^, ^4^, ^5^, ^6^, ^7^, ^8^, ^9^, ^10^, ^11^, ^12^ ^]^ Thermodynamic stability is one of the main benefits of heterocycles, but it is also their downside because the corresponding molecules are not responsive to the environments they are placed in. In this paper, we investigate pseudo‐rings as conformational control elements. In small molecules, pseudo‐ring frameworks can mimic the structural features of their heterocyclic congeners. This was first demonstrated by Hodge and Pierce in 1993. In that work, 4‐aminoquinazolinone was shown to mimic the structure of the hydrogen‐bound, 6‐membered ring of a salicylamide‐containing scytalone dehydratase (SD) inhibitor (Figure 1, pyrimidine to hydroxyamide).^[^ ^13^ ^]^ Since then, a handful of other examples have surfaced where 6‐membered hydrogen‐bonded pseudo‐rings serve as mimetics of aromatic heterocycles (Figure 1).^[^ ^14^, ^15^, ^16^ ^]^ We hypothesized that pseudo‐rings held together by hydrogen bonds might lead to discrete conformational states in peptide macrocycles. Like in small molecules, the 6‐membered hydrogen‐bonded residue in a macrocycle is likely to exist in one of two forms: a closed form (with an intact 6‐membered hydrogen‐bound ring) or an open form (with a disrupted 6‐membered hydrogen‐bound ring).^[^ ^17^ ^]^ A parallel can be drawn to the transannular hydrogen bond networks formed in the backbones of peptides, although those interactions are typically weaker and longer in range. Transannular hydrogen bonds between amino acid residues of the peptide backbone can exist depending on the environment.^[^ ^18^ ^]^ Conformational switching is pivotal to the properties of many cyclic peptides, most notably cyclosporine A.^[^ ^18^, ^19^ ^]^ Our work suggests that the modulation of open and closed forms of pseudo‐rings can serve as an effective element of structural control otherwise not achievable using conventional methods and may offer a new method for conformational switching in macrocycles.

## 2 Results and Discussion

**Figure 2:**
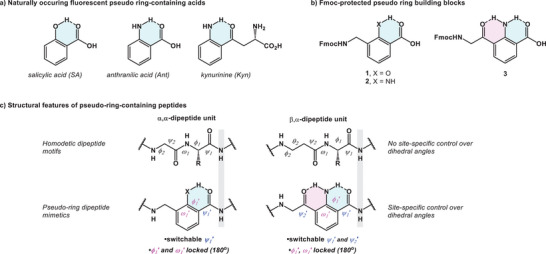
a) Naturally occurring pseudo‐ring residues; b) Fmoc‐protected pseudo‐ring building blocks; c) structural features of homodetic peptides and pseudo‐ring peptides.

An early report by Etter outlining the trends of hydrogen bond formation indicated that “six‐membered‐ring intramolecular hydrogen bonds form in preference to intermolecular hydrogen bonds.” ^[^ ^20^, ^21^ ^]^ In nature, there are three well‐defined non‐proteinogenic residues that feature such a 6‐membered intramolecular hydrogen‐bound pseudo‐ring structure. These molecules, salicylic acid (SA), anthranilic acid (Ant), and kynurenine (Kyn), all contain a phenol or aniline hydrogen bond donor flanked by a carbonyl hydrogen bond acceptor (Figure 2a). With these naturally occurring blocks in mind, we devised Fmoc‐protected pseudo‐ring‐containing building blocks **1**–**3** that are compatible with conventional Fmoc SPPS protocol (Figure 2b). Both **1** and **2** feature a single pseudo‐ring formed between a phenol or aniline hydrogen bond donor and the adjacent carbonyl oxygen. The building block **3** features two pseudo‐rings as its central aniline portion can form 6‐membered hydrogen bond networks with the carboxylic acid oxygen (or amide oxygen once incorporated into the backbone of the peptide) and the adjacent ketone oxygen. In typical homodetic peptides, the dihedral angles *ϕ*_1_ */ψ*_1_ and *ϕ*_2_ */ψ*_2_ (in an α,α‐amino acid dipeptide unit) or *ϕ*_1_ */ψ*_1_ and *ϕ*_2_ */θ*_2_ */ψ*_2_ (in a β,α‐amino acid dipeptide unit) are influenced by intramolecular hydrogen bonding (IMHB) interactions. We hypothesized that pseudo‐ring frameworks would allow for control of dihedral angles *ψ*_1_′, *ψ*_2_′, and *ϕ*_1_′, while locking *ω*_1_′ at 180° (Figure 2c). Given these unique features of pseudo‐ring peptides, we were keen to answer three questions: 1) Would each individual pseudo‐ring be conserved once **1**–**3** are inserted into the backbone of a macrocycle? 2) How would the photophysical properties of backbone‐embedded pseudo‐ring‐containing peptides differ from their small molecule counterparts? and 3) Would the inclusion of **1**–**3** improve the solubility and the lipophilicity of a macrocycle when compared to its all‐amino acid congener?

**Figure 3:**
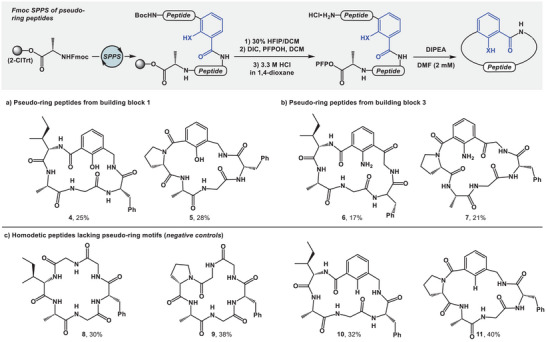
The general synthetic strategy to access: a) pseudo‐ring peptide from building block **1** (**4**–**5**); b) pseudo‐ring peptides from building block **3** (**6**–**7**); c) homodetic peptides **8**–**11**.

We considered building blocks **1**–**3** as Fmoc‐protected pseudo‐ring building blocks that could be implemented into the Fmoc SPPS approach. The building block **1** was readily prepared from 3‐methyl SA (see Supporting Information). Despite our efforts, building block **2** was not accessible, as all attempts to produce the aniline from the corresponding nitroaromatic starting material resulted in low conversions and yields. The building block **3** was made from commercially available 7‐carboxyindole over seven steps. This procedure was scalable and required no chromatographic purifications (see Supporting Information). With **1** and **3** in hand, we turned our attention towards their application in SPPS. Remarkably, **1** and **3** proved to be compatible with Fmoc SPPS protocols without the need to protect the OH or NH_2_ functionality, which speaks to the stability of strong intramolecular hydrogen bonds in these molecules (see Supporting Information). Through cyclization of the linear pentafluorophenol (PFP) ester at high dilution in DMF with DIPEA, cyclic peptides **4** (25% overall yield) and **5** (28% overall yield) were obtained from building block **1**. The cyclic peptides **6** (17% overall yield) and **7** (21% overall yield) were prepared from building block **3** (Figure 3a,b). At the same time, we synthesized cyclic peptide controls devoid of the pseudo‐ring core through a conventional Fmoc SPPS strategy (Figure 3c) with a Gly‐Gly in place of the pseudo‐ring scaffold. Similarly, we prepared peptides **10** and **11**, which represent depeptidized homodetic sequences that contain a backbone‐embedded arene that lacks the X─H pseudo‐ring hydrogen bond donor (Figure 3c). To draw further comparison between pseudo‐ring‐containing and regular heterocycle‐containing peptides, we attempted to synthesize the indole‐containing analogues of peptides **6** and **7** (see Supporting Information). However, peptides **S22a** and **S22b** proved to be difficult to obtain, and their syntheses gave only trace amounts of each desired macrocycle. During the preparation of cyclic peptides **4**–**11**, we noticed that macrocycles devoid of a pseudo‐ring were frustratingly insoluble in all organic solvents as well as in water. As result, these peptides (**8**–**11**) were purified by hot trituration in methanol/water. The solubilities/lipophilicities of peptides **4**–**11** will be discussed later in this paper.

### 2.1 Structural analysis

**Figure 4:**
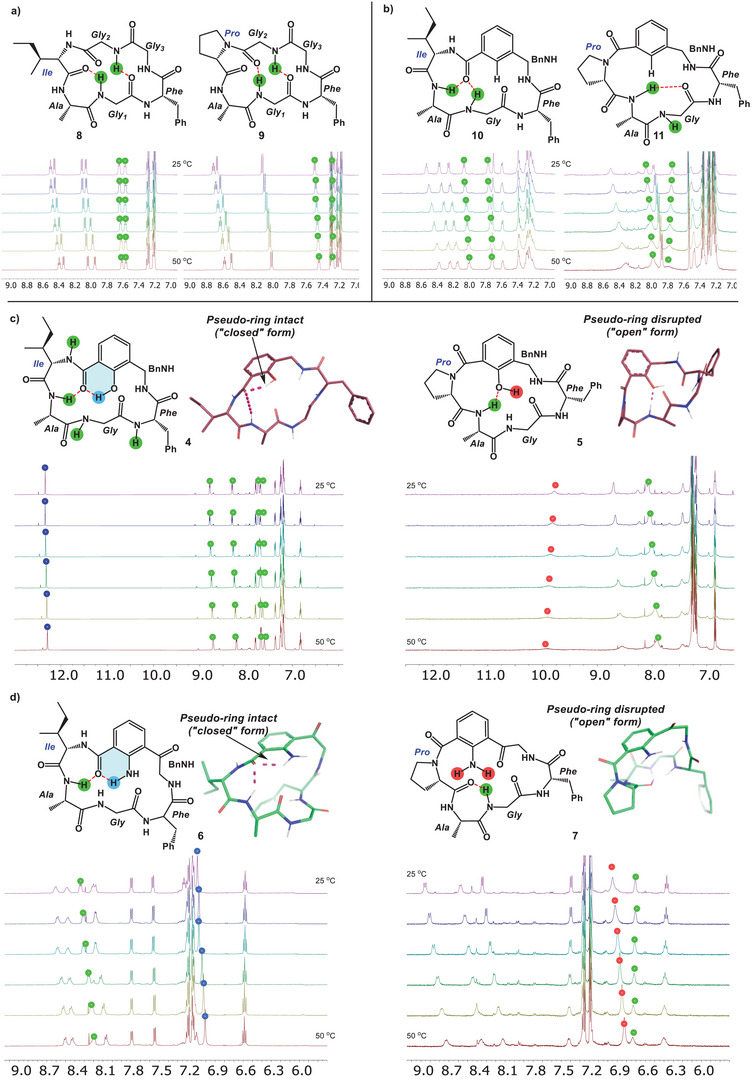
The preferred conformations and VT‐NMR of peptides **4**–**11** in *d6*‐DMSO.

We began our study by performing structural analysis of homodetic peptides **8**–**9** and **10**–**11**, which lacked any pseudo‐ring motifs. Peptides **8–11** proved to be only sparingly soluble in *d_6_*‐DMSO and, as a result, sonication and gentle heating were required to adequately solubilize these samples at concentrations necessary for NMR analysis (ca. 10 mg/mL). These peptides were subjected to solvent‐explicit molecular dynamics (OPLS4) simulations as well as variable–temperature (VT) ^1^H‐NMR analysis. Despite their poor solubilities in DMSO, both **8** and **9** displayed well‐organized secondary structures. In **8**, two turn elements were observed (*γ*‐ and *β*‐turns), whereas **9** was found to contain two facing *β*‐turns (Figure 4a). In both peptides, the NH donors (Gly1 and Gly2) remained the same. The Pro residue in **9** led to differences in *ϕ*_1_/*ψ*_1_ (32°/−103° in **8** and −176° /−134° in **9**) but to little effect in *ϕ*_2_/*ψ*_2_ (4°/10° in **8** and 4°/96° in 9). We were interested in determining the structural effects of including a backbone‐embedded arene (peptides **10** and **11**). The inclusion of the backbone‐embedded arene removes a single H─bond donor (Gly2NH) and locks angle *ϕ*_1_′ to 180° (Figure 4b). Much like in the fully peptidic homodetic macrocycles, the orientation of the aryl C═O could be altered through the inclusion of a Pro residue at the arene C‐terminus (*ψ*_1_′ = 120° in **10** and −60° in **11**). Dislocation of the carbonyl in **11** results in the removal of two key IMHB between the NH of the Ala and the Gly. This results in a much more flexible system, which is evidenced by the broad and poorly defined NMR spectrum of **11** at room temperature. In **10** and **11**, we could not reliably alter the secondary structure. Similarly, we found that only changing *ψ*_1_′ resulted in no significant changes in the number of amides with low temperature coefficients (likely involved in IMHB) within **10** and **11**. Next, we were keen to determine whether inclusion of a pseudo‐ring residue would enable further modulation of the *ψ*_1_′ angle. Our structural analysis of pseudo‐ring peptides began with **4** and **5** in *d_6_*‐DMSO (Figure 4c). Both peptides were readily soluble in *d_6_*‐DMSO, allowing for straightforward NMR analysis. Solvent explicit molecular dynamics (OPLS4) simulations as well as VT ^1^H‐NMR analysis revealed that peptide **4** indeed retained a rigid H─bond between the phenolic hydrogen and the adjacent carbonyl (*ψ*_1_′ = 140°). The VT coefficient of pseudo‐ring phenol OH resonance was found to be 2 ppb/K. Typically, temperature coefficients less than 4 ppb/K indicate that a hydrogen bond donor is non‐solvent exposed and is involved in an IMHB interaction.^[^ ^22^ ^]^ In stark contrast, peptide **5** lacked this pseudo‐ring element. This was immediately recognized by looking at the VT coefficient of the phenol OH resonance. In peptide **5**, the VT coefficient of the phenol OH resonance was determined to be 5.6 ppb/K, indicating that the pseudo‐ring had been completely disrupted. In linear peptides, a similar phenomenon was observed by Ito and coworkers.^[^ ^23^ ^]^ In their work, the 6‐membered hydrogen bond network of Ant could be disrupted upon coupling the C‐terminal carboxylate of Ant with secondary amines (such as dimethyl amine or proline). However, the carbonyl group in our pseudo‐ring peptide **5** was oriented unlike that of Ito and coworkers. In peptide **5**, the carbonyl of arene is oriented close to perpendicular to the aromatic plane (*ψ*_1_′ = −66°). This modulation of *ψ* ′ results in a significant downstream structural effect within the peptide macrocycle. With the pseudo‐ring intact in peptide **4**, all amides except the benzyl amide NH were found to be shielded from the solvent, and many of them were likely involved in IMHBs, which is evident by their low temperature coefficients. In **4**, the AlaNH forms a *γ*‐turn with the C‐terminal C═O connected to the aryl group. Disruption of the pseudo‐ring H─bond in **5** results in perturbation of all IMHBs except that of the AlaNH. However, upon looking at the preferred conformation of **5** from the MD simulation, a 9‐membered IMHB was formed between the AlaNH and the hydrogen bond‐accepting phenolic oxygen. This result is interesting as it indicates that, when embedded within a macrocycle, the pseudo‐ring framework is environment‐sensitive. Like previously mentioned peptides, incorporation of the Pro residue and, in the case of peptide **5**, disruption of the pseudo‐ring result in a highly flexible conformation, which is evident by the broad and poorly defined NMR spectrum of **11** at room temperature. We were keen to further investigate the modulation of pseudo‐ring IMHBs by studying the more complex dicarbonyl‐containing pseudo‐ring peptides **6** and **7**. In small molecules, dicarbonyl systems such as 2‐aminoisophthalic acid form two equivalent hydrogen bonds from the aniline NH_2_.^[^ ^24^ ^]^ Like the previous peptides, **6** and **7** were also soluble in *d_6_*‐DMSO, allowing for easy NMR analysis. VT‐NMR and MD analysis of **6** revealed an asymmetry in the hydrogen bond‐forming abilities of the two C═O flanking the central aniline NH_2_ (Figure 4d). The VT coefficient of the aniline NH_2_ was found to be 3.2 ppb/K, indicating the presence of an IMHB of moderate strength. However, analysis of the most populated MD conformation revealed that the anthranilamide‐like pseudo‐ring (in blue) remained intact, while the Kyn‐like pseudo‐ring (depicted in pink in **3** in Figure 3) was disrupted. This finding is reflected in the values of *ψ*_1_′ and *ψ*_2_ ′, which are −151° and 89°, respectively. The orientation of the ketone is surprising as it lies perpendicular to the arene plane. Further, a similar IMHB formed with the AlaNH residue observed in **4** was also observed in peptide **6**. In fact, despite having an additional carbonyl oxygen, the conformations of peptides **4** and **6** were nearly identical in *d_6_*‐DMSO. As mentioned above, a Pro residue conjoined to the C‐terminus of the pseudo‐ring residue resulted in the disruption of its 6‐membered IMHB. In peptide **6**, both pseudo‐ring systems become disrupted, leading to a fully “open” pseudo‐ring conformation (Figure 4d). This “open” conformation is distinguished by significant variation of *ψ*_1_′ (−77°) and *ψ*_2_ ′ (139°) angles. The dislocation of this anthranilamide‐like pseudo‐ring has a two‐fold downstream structural effect. The Kyn‐like C═O contorts, now sitting more closely in the plane of the arene, which is a remarkable push/pull effect within the macrocycle. As one pseudo‐ring deplanarizes, the other hydrogen bond‐accepting pseudo‐ring C═O reorients itself to lie more closely in plane with the hydrogen bond‐donating NH_2_. The second downstream structural effect of this “open” conformational form is the translocation of other backbone IMHBs. The g‐turn of peptide **6** (AlaNH/Aryl C═O) is translocated by two residues and is formed between the GlyNH and the C═O of the pro residue. These results indicate that “open” and “closed” pseudo‐rings allow for significant alteration of the secondary structures of peptide macrocycles. In the “closed” forms, pseudo‐rings containing peptides seem to prefer the formation of tight transannular IMHB formed between the C‐terminus aryl C═O. At the same time, in the “closed” pseudo‐ring **4**, we observe fewer solvent‐exposed amides. In contrast, “open” states lead to disruption of these tight turns and lead to the preferential formation of hydrogen bonding interaction with NH donors located further downstream of the pseudo‐ring system.

### 2.2 Fluorescence study

**Figure 5:**
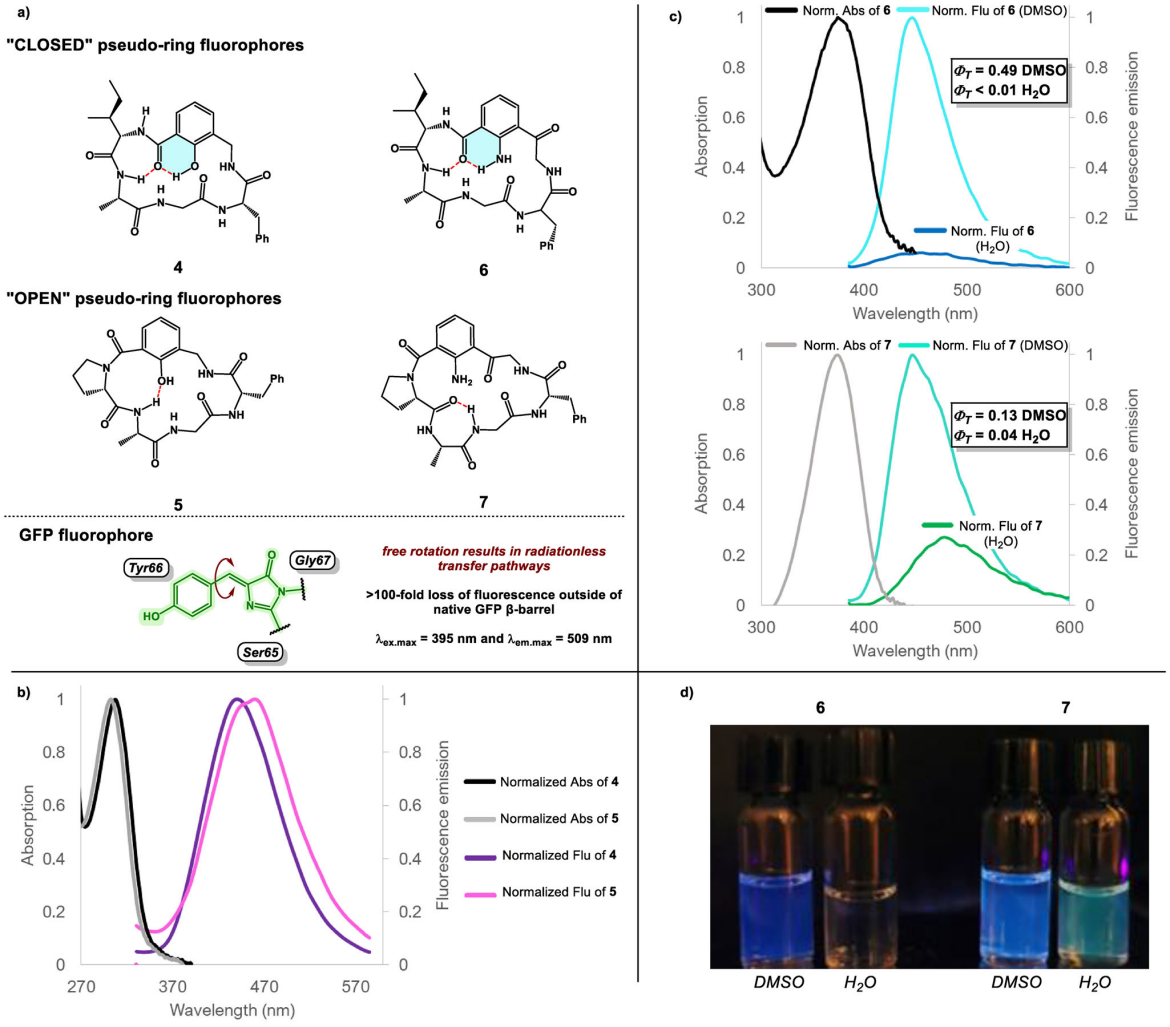
a) Structures of fluorescent peptides; b) normalized absorption and fluorescence of peptides **4**–**5** in DMSO; c) normalized absorption and fluorescence of peptides **6**–**7** in DMSO/H_2_O; d) visual fluorescence of peptides **6**–**7** in DMSO/H_2_O (ca. 10 mM).

With the structural insights in hand, we turned our attention to the photophysical properties of pseudo‐rings embedded into macrocycle backbones. Small molecules SA, Ant, and Kyn are all inherently fluorescent. The unique fluorescent properties of SA, Ant, and Kyn can be attributed to intramolecular proton transfer—from the OH/NH_2_ to the adjacent carbonyl—made possible by the 6‐membered pseudo‐ring.^[^ ^25^ ^]^ However, SA (*Φ_T_* = 0.022 in cyclohexane), Ant (*Φ_T_* = 0.40 in methanol), and Kyn (*Φ_T_* = 0.018 in neutral water) are not suitable backbone‐embedded fluorophores.^[^ ^23^, ^26^, ^27^ ^]^ SA and Ant do not retain their fluorescence upon acylation of the XH group, making them ineffective as backbone‐embedded fluorophores within macrocycles, whereas Kyn is located within the side chain. The photophysical properties of peptides **4**–**7** (Figure 5a) were characterized by optical absorption and fluorescence measurements. Peptides **4** and **5** both showed weak fluorescence in DMSO with λ_ex_._max_ of 310 and 305 and λ_m_._max_ of 445 nm and 447 nm, respectively (Figure 5b). Interestingly, **5** showed significantly weaker fluorescence than **4**. This is likely a result of the flexibility of the fluorophore system—a consequence of pseudo‐ring disruption—leading to increased radiationless transfer. The Stokes shift in both peptides is rather large (130 and 140 nm). This emission band is characteristic of the keto/enol tautomeric form in SA and is likely involved in the fluorescence of peptides **4** and **5**. Despite this, the fluorescence intensities of **4** and **5** were quite low in DMSO, and thus their quantum yields were not measured. We were hopeful that our next pseudo‐ring system—the one based on the building block **3**—would have an improved fluorescent intensity and resulting quantum yield. To our delight, we found that peptides **6** and **7** were highly fluorescent in DMSO. The λ_ex.max_ values of **6** and **7** are 383 nm and 374 nm, respectively, while the λ_em.max_ values of **4** and **5** were 447 nm. Peptide **6** proved to be exceptionally fluorescent with a quantum yield of 0.49 in DMSO (Figure 5c). Again, the “open” pseudo‐ring in **7** results in attenuated fluorescence with a quantum yield of 0.28 in DMSO. As previously mentioned, this is likely a result of the flexibility of the fluorophore system leading to increased radiationless transfer. Interestingly, despite having an extra electron‐withdrawing carbonyl group on the arene of the fluorophore, the photophysical properties of pseudo‐ring‐containing **6** and **7** closely matched that of native Kyn. Computationally, we determined that the key transition during photoexcitation takes place between the non‐bonding orbital of the aniline NH_2_ and the antibonding orbital of the flanking ketone C═O (see Supporting Information). This indicates that the second anthranilamide‐like pseudo‐ring (NH_2_ to amide C═O) has little impact on the excitation and the emission profile of the fluorophore. We were interested to understand how the photophysical properties of these peptides would be altered when moving to an aqueous environment. Typically, in small molecules, the fluorescence of pseudo‐ring‐containing molecules is often almost completely quenched in aqueous environments. Hydrogen bonding of the solvent with electron acceptors stabilizes the intramolecular charge transfer (ICT) state, enhancing the internal conversion that leads to quenching of the ICT state in many molecules.^[^ ^25^ ^]^ This phenomenon has been used to great effect in studying membrane binding of the Kyn‐containing antibiotic daptomycin. In daptomycin, membrane permeation results in significant enhancement of the quantum yield when the peptide transitions from a polar aqueous environment to the non‐polar lipid bilayer. In water, the fluorescence of **6** is greatly reduced with a dramatic 43‐fold reduction in the quantum yield of this compound (Figure 5d). Evidently, in the case of **6**, water molecules can interact with the fluorophore, leading to significant fluorescent quenching. Remarkably, we did not observe fluorescence quenching when we studied **7** in water. In this case, the λ_em.max_ red‐shifts significantly (478 nm), and we observed only a 3.5‐fold reduction in the fluorescence of **7** when compared to DMSO. In water, it is likely that the Kyn‐like fluorophore of **7** becomes shielded from the aqueous environment by embedding itself into the core of the macrocycle. The behavior of this system is not unlike that of native GFP. The native fluorophore of GFP is only weakly fluorescent outside of the β‐barrel of the GFP protein due to significant radiationless transfer pathways.^[^ ^28^, ^29^, ^30^ ^]^ However, when located within the GFP protein framework, the GFP fluorophore fluorescence profile is enhanced (>100‐fold).^[^ ^30^ ^]^ Our study demonstrates that conformational changes in macrocycles equipped with pseudo‐rings can result in attenuated fluorescent profiles without requiring the complexity of the native GFP protein.

### 2.3 Solubility and lipophilicity study

The solubility of peptide macrocycles can be altered through the inclusion of polar groups to improve aqueous solubility and non‐polar groups to improve solubility in lipophilic media. Despite this, accessing highly soluble macrocycles is challenging. *N*‐methylation of backbone amides has long been the classical way of augmenting the lipophilicity of cyclic peptides.^[^ ^31^ ^]^ The *N*‐methylation strategy is inspired by the chameleonic naturally occurring peptide cyclosporine A, which shows excellent passive permeability but poor aqueous solubility. More recently, the Lokey group has been able to improve membrane permeability and lipophilicity by sequestering amide bond NHs through interaction with side chain hydrogen bond acceptors.^[^ ^22^ ^]^ In contrast to other findings, work by Fairlie has suggested that peptide permeability is largely dictated by the connection of the peptide's hydrophobic surfaces rather than transannular hydrogen bonding interactions.^[^ ^32^ ^]^ We suspected that pseudo‐rings could similarly mask polar groups, thus augmenting the macrocycle lipophilicity. Interestingly, in a small molecule prodrug of psilocybin, Lenz et al. demonstrated that the energetic gains from pseudo‐ring IMHB impede the protonation of the hydrogen bond‐accepting atom (*N* of an amine) and impede the deprotonation of the hydrogen bond‐donating group (phenolic OH).^[^ ^33^ ^]^ Another consequence of IMHB is that the polar groups within this psilocybin prodrug self‐mask themselves, resulting in an increased free energy of solvation in apolar media. Altogether, Lenz et al. demonstrated that this pseudo‐ring strategy was effective in improving the cell‐penetrating ability of the prodrug and that the ability of a sequence to form IMHBs results in a higher number of uncharged species that can cross the blood–brain barrier.^[^ ^33^ ^]^

**Figure 6:**
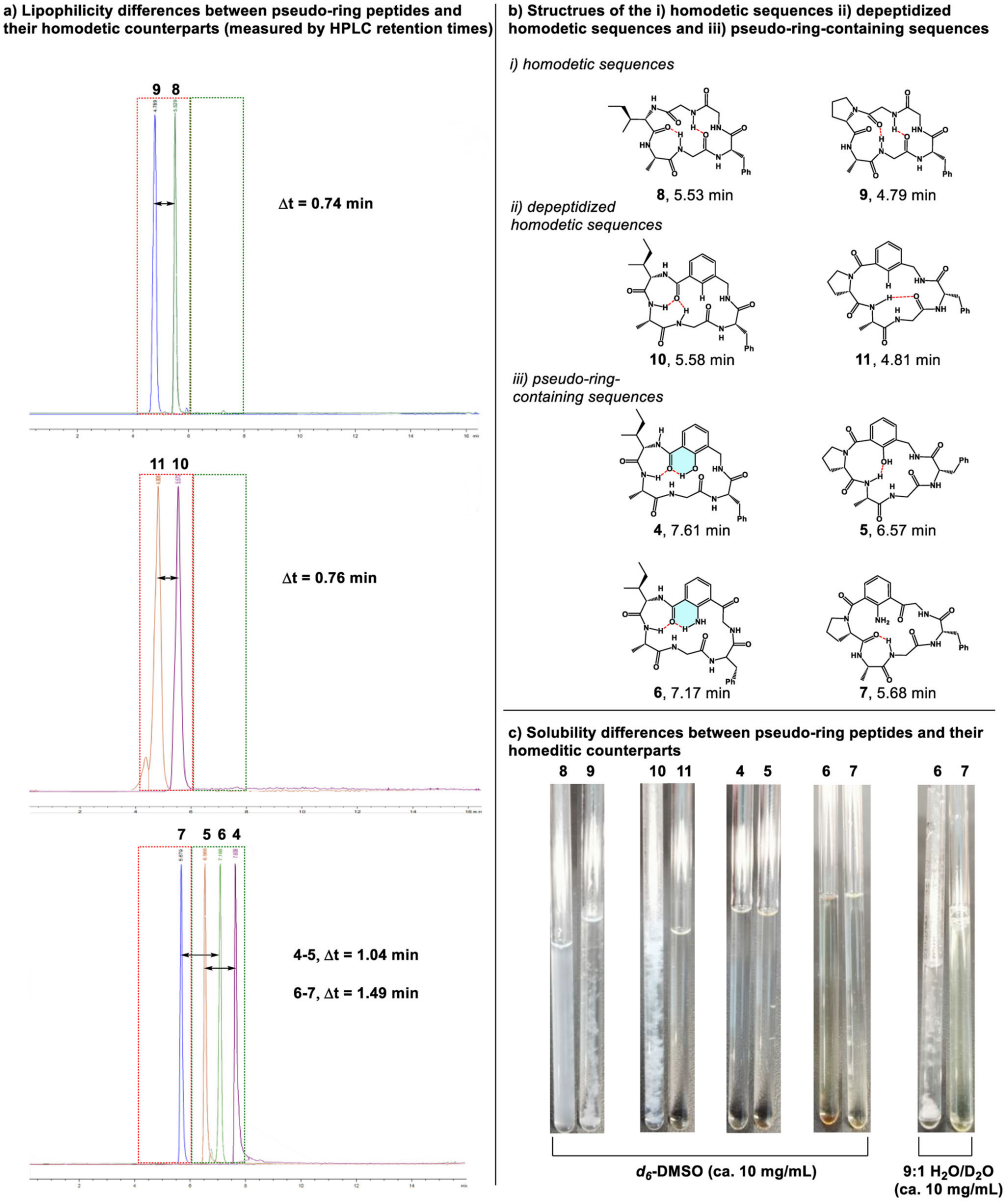
a) HPLC retention times of peptides **4**–**11** on a 15 min run from 5% MeCN to 95% with 0.1% formic acid (red boxed area ═ retention times using homodetic sequences, green boxed area ═ retention times using pseudo‐ring dipeptide mimetics, peaks represented extracted MS signals) b) structures of i) homodetic peptides **8**–**9,** ii) depeptidized homodetic peptides **10**–**11**, iii) pseudo‐ring containing sequences **4**–**7** c) Solubility differences between pseudo‐ring peptides and their homodetic sequence counterparts in DMSO and H_2_O.

Reversed‐phase HPLC retention times have proven to be a useful metric in determining the permeability of peptide macrocycles, as longer retention times typically equate to lower aqueous solubility, lower polarity, and increased lipophilicity.^[^ ^34^, ^35^, ^36^, ^37^ ^]^ We were keen to use these HPLC retention times as a tool to assess the modulation of lipophilicity of the pseudo‐ring‐containing macrocycles. At the same time, we were interested in collecting solubility measurements for each macrocycle in both organic solvents (DMSO) as well as in water by visually determining each macrocycle's solubility. Our analysis began with the proteinogenic peptides **8** and **9** (Figure 6a). From the NMR studies, we recognized that these peptides had extremely poor solubility in *d_6_*‐DMSO (Figure 6c). Indeed, we found that peptides **8** and **9** could not be fully solubilized in *d_6_*‐DMSO at room temperature at a concentration of 10 mg/mL. HPLC analysis of these peptides confirmed that proteinogenic sequences correspond to extremely polar peptides. On a 15‐min HPLC gradient (from 5% MeCN/H_2_O to 95% MeCN/H_2_O with 0.1% formic acid), macrocycles **8** and **9** had HPLC retention times of 5.3 min and 4.8 min, respectively. Both **8** and **9** contain similar hydrogen bonding patterns, which is reflected in their similar hydrophilicities and HPLC retention times. Similar results were obtained with peptides **10** and **11** (Figure 6a). Much like with the previous macrocycles, compound 10 was poorly soluble in *d_6_*‐DMSO at room temperature (at 10 mg/mL), while macrocycle **11** could be solubilized in *d_6_*‐DMSO at room temperature only after sonication (at 10 mg/mL) (Figure 6c). Despite these solubility differences, both molecules were found to have very short HPLC retention times. In both cases, two amides have low temperature coefficients (indicating that they are likely involved in IMHB), explaining why both sequences have very similar HPLC polarities/HPLC retention times (5.6 min and 4.8 min, respectively). Interestingly, our data indicated that the solubility and lipophilicity of these 18‐membered macrocycles cannot be easily modulated through the variation of a single residue (Ile versus Pro). We were keen to decipher whether the inclusion of either “open” or “closed” pseudo‐ring residues could lead to noticeable solubility and lipophilicity differences. It became clear that pseudo‐ring‐containing macrocycles **4** and **5** were much more soluble in DMSO than their non‐pseudo‐ring counterparts (Figure 6a). Likewise, we have found that macrocycle **4** (containing the “closed” phenolic pseudo‐ring moiety) was much more lipophilic than the other peptide. Macrocycle **4** was found to have the HPLC retention time of 7.7 min. With an open pseudo‐ring, peptide **5** was found to be more hydrophilic than the closed pseudo‐ring‐containing macrocycle **4** with an HPLC retention time of 6.6 min. This increase in hydrophobicity likely results from the now exposed polar phenolic OH group paired with the disrupted IMHB networks that were present within the backbone of **4**. This modulation of “open” and “closed” pseudo‐rings leads to HPLC retention times differing by over 1 min, whereas in the previous homodetic macrocycles, HPLC retention times were found to differ by only ∼0.7 min. All the macrocycles up until this point have proven to be poorly soluble in water. With this in mind, we began analyzing our dicarbonyl‐containing macrocycles **6** and **7**. Macrocycles **6** and **7**, much like in the case of the previous pseudo‐ring molecules, proved to be highly soluble in DMSO (at a concentration of 10 mg/mL) (Figure 6c). However, unlike all the other peptides studies up until this point, the “open” pseudo‐ring macrocycle **7** was found to be highly soluble in H_2_O (at a concentration of 10 mg/mL) (Figure 6c). This result is interesting as, up until this point in our lab's work, we have not been able to synthesize a macrocycle that was readily soluble in both an organic and an aqueous environment. Similarly, this marks the first instance in which we have been able to access a class of macrocycles with chameleonic behavior whereby we can reliably access both “open” and “closed” forms using pseudo‐rings. When looking at the lipophilicities of the macrocycles, we see that the closed pseudo‐ring macrocycle **6** is notably more lipophilic than the homodetic peptides **8**–**11** with an HPLC retention time of 7.2 min (Figure 6a). Unique to this series of macrocycles, the “open” pseudo‐ring macrocycle **7** is extremely hydrophilic. The HPLC retention time of 5.6 min of **7** indicates that this macrocycle has become much more hydrophilic when compared to the closed pseudo‐ring macrocycle **6**. This greatly increased hydrophilicity is not something we have observed with the previous macrocycles. The ability to improve the aqueous solubility and hydrophilicity of a macrocycle is a powerful tool that we have not been able to demonstrate in our lab up until now. We believe that the dicarbonyl pseudo‐ring block will prove to be a useful moiety for others as they design macrocycles with improved solubilities and permeability.

## 3 Conclusion

In this study, we have shown that pseudo‐rings represent powerful elements of conformational control in peptide macrocycles. The pseudo‐ring building blocks described in this paper enable an elusive ability to constrain torsional angles of the dipeptide mimetic. At the same time, the preferred conformation of the pseudo‐ring mimetic can be tailored, allowing for nuanced alteration of dihedral angle *ϕ*′ in macrocycles containing building block **1** and dihedral angles *ϕ_′_*/*ϕ*_2_′ in macrocycles containing building block **3**. We have demonstrated that the replacement of a polar dipeptide unit within the macrocycle backbone with pseudo‐ring block leads to molecules with improved solubility in organic solvents when compared to analogous non‐pseudo‐ring‐containing peptide macrocycles. Strategic placement of a tertiary amide at the C‐terminus of a peptidic pseudo‐ring leads to deplanarization of the 6‐membered hydrogen‐bound system. This concept was applied to a novel dicarbonyl aniline‐containing pseudo‐ring system, inspired by non‐proteinogenic amino acids Kyn and Ant. This resulted in a peptide macrocycle with high aqueous solubility. At the same time, we have shown that our backbone pseudo‐ring amino acids are highly fluorescent molecules (*Φ*_T_ ∼ 0.5). The quantum yield of these fluorophores can be modulated through the deplanarization and increased flexibility of the 6‐membered H‐bound ring. Most importantly, we have demonstrated that backbone‐embedded pseudo‐ring fluorophores can mimic the unique fluorescent properties of the native GFP protein. In aqueous environment, the non‐planar pseudo‐ring fluorophore can embed itself within the inner core of the peptide macrocycle. This phenomenon shields the fluorophore from rapid quenching by water and results in minimal loss is fluorescence intensity and a significant red shift to the emission wavelength. The unique properties of our pseudo‐ring building blocks have applications as environmentally sensitive fluorophores. For example, the permeation of a pseudo‐ring‐bearing macrocycle can be visually tracked by its fluorescence. The fluorescence of the pseudo‐ring moiety blue‐shifts as it begins to enter the cellular membrane and then red‐shifts as it re‐enters an aqueous environment (in the intracellular environment). Unlike other similar fluorophores, the fluorescence of our pseudo‐ring is not quenched in aqueous environments meaning that tracking the specific location of the pseudo‐ring‐containing macrocycle in in vitro and in vivo assays is now made possible.

We propose the following strategy to implement pseudo‐ring replacement in macrocycles: 1) Recognize a polar dipeptide sequence, such as a Gly‐Gly motif, where one or more of its amides NH or amide C═O are involved in IMHB, and 2) replace this dipeptide with dipeptide mimetic **1** or **3** to create pseudo‐ring imbedded macrocycles. This strategy should result in macrocycles with modified conformations where the pseudo‐ring C‐terminal carbonyl or the pseudo‐ring aryl heteroatom (X group) could act as the backbone “structural pin” (i.e., a H─bond that is primarily responsible for stabilizing the entire macrocycle backbone conformation).^[^ ^19^ ^]^ Further, pseudo‐ring‐containing macrocycles should have much improved solubilities and lipophilicities when compared to the original homodetic sequence. We suspect that many other pseudo‐ring frameworks could be designed using our findings and applied in efforts to access macrocycles with improved solubilities, permeabilities, and unique conformation features.

## Conflict of Interests

The authors declare no conflicts of interest.

## Supporting information



Supporting Information

## Data Availability

Additional references cited within the Supporting Information.^[^ ^38^, ^39^, ^40^, ^41^, ^42^, ^43^, ^44^, ^45^, ^46^, ^47^, ^48^, ^49^, ^50^, ^51^, ^52^, ^53^, ^54^, ^55^, ^56^ ^]^ The data that support the findings of this study are available in the supporting information of this article, which is available at: https://doi.org/10.1002/chem.202500581.
