# Supplementary material for: Pseudo‐Ring Methodology to Control the Conformations of Macrocyclic Peptides
Source: Chemistry. 2025 Mar 27;31(24):e202500581. doi: 10.1002/chem.202500581 (PMC12043042; doi:10.1002/chem.202500581)
Supplement: Supplementary file 1 — Supporting Information [file CHEM-31-e202500581-s001.pdf]

Table of Contents

General Experimental Method	2
Building block syntheses	3-6
Building block NMR	7-14
Solid-Phase Peptide Synthesis	15-17
Characterization of peptides **4-11**	18-57
LC traces (cyclization of **S22a** and **S22b**)	58-29
Conformational Analysis	60-64
Photophysical data	65-71
Acknowledgements	72
References	73-74

General Experimental Method

All reagents were utilized as received from commercial sources unless otherwise noted. All solvents were of reagent grade quality and freshly distilled prior to use. Dichloromethane was distilled over CaH_2 indicator under an atmosphere of nitrogen. Chromatography: Flash-column chromatography was performed using Merck silica gel 60 (40- 63 µm). Thin-layer chromatography (TLC) was performed using Merck silica gel 60 F 254 plates, with UV (254 nm) detection followed by $KMnO_4$ or Ninhydrin stain. NMR Spectrometry: All NMR spectra were recorded on either a Bruker DPX300, Bruker AV 300, Bruker AV 400 at 300 K, Agilent 500 MHz DD2 NMR Spectrometer at 298 K or a Varian 600 Unity spectrometer at 298 K. 1H NMR spectra chemical shifts (δ) are reported in parts per million (ppm) referenced to residual protonated solvent peak (DMSO-*d*6 δ = 2.50). Spectral data is reported as follows: chemical shift, multiplicity (s = singlet, d = doublet, t = triplet, q = quartet, dd = doublet of doublets, dt = doublet of triplets, ddt = doublet of doublet of triplets, dtd = doublet of triplet of doublets, m = multiplet, br = broad, h = heptet, dddd = doublet of doublet of doublet of doublets, qd = quartet of doublets, td = triplet of doublets, tt = triplet of triplets), coupling constant (J) in Hertz (Hz), and integration. 13C NMR spectra chemical shifts (δ) are reported in parts per million (ppm) and were referenced to carbon resonances in the NMR solvent (DMSO-*d*6 δ = 39.5). Mass Spectrometry: High-resolution mass spectrometry (HRMS) ESI (m/z) spectra were recorded on a Bruker MicroTof or an Orbitrap LTQ XL (Nanospray) of Thermo Scientific. At the University of Toronto high resolution mass spectra were obtained on a VG 70-250S (double focusing) mass spectrometer at 70 eV or on an ABI/Sciex Qstar mass spectrometer with ESI source, MS/MS and accurate mass capabilities or on JEOL AccuTOF-DART instrument. RP-HPLC/MS: Low-resolution mass spectra (ESI) were collected on an Agilent Technologies 1200 series HPLC paired to a 6130 Mass Spectrometer. Compounds were resolved on Phenomenex's Kinetex 2.6µ C18 50x4.6mm column at room temperature with a flow of 1 mL/min. The gradient consisted of eluents A (0.1% formic acid in double distilled water) and B (0.1% formic acid in HPLC-grade acetonitrile). LCMS method: A linear gradient starting from 5% of B to 95% over 15 min or 6 min at a flow rate of 1.0 mL/min.

Experimental Procedure

Building block syntheses

Fmoc protected pseudo ring building blocks **1** and **3** were synthesized and implemented in a Fmoc SPPS protocol to prepare macrocycles **4-7**. Failed attempts to prepare building block **2** are also described below.

Scheme S1. Synthesis of building block 1.

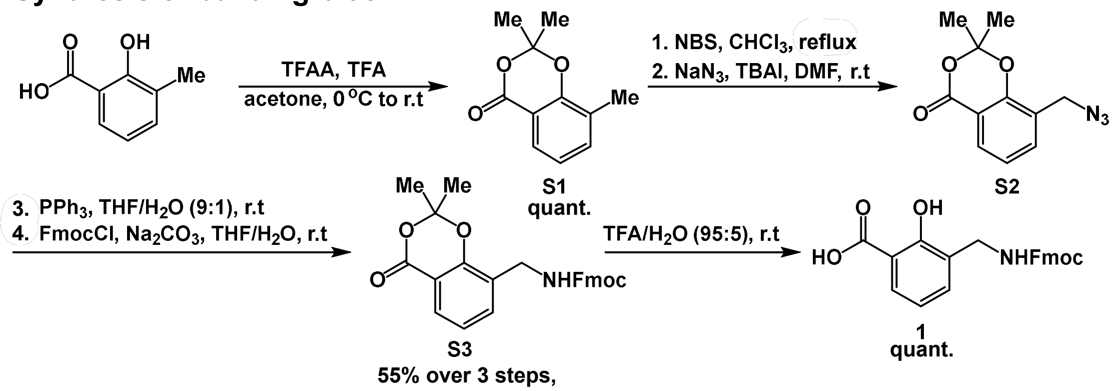

Compound S1: Acetonide protected **S1** was made following previously reported literature conditions.[39] To an ice-cold suspension of substituted salicylic acid 1 (3.0 g) in trifluoroacetic acid (24 mL) were added trifluoroacetic anhydride (15 mL) and acetone (3 mL). The mixture warmed slowly to room temperature and then stirred for 16 h. The slightly yellow solution was then concentrated on the rotary evaporator, poured into a saturated solution of aqueous $NaHCO_3$ (200 mL), and extracted EtOAc (100 mLx3). Pooled extracts were washed with water and brine, dried (Na_2SO_4), and concentrated to leave the crude product. Silica FC eluting with 5% EtOAc/hexanes as eluent afforded **S1** (3.79 g) as an off white solid in a quantitative yield. ^{1}H NMR (500 MHz, cdcl$_3$) δ 7.82 – 7.77 (m, 1H), 7.39 (ddd, *J* = 7.4, 1.5, 0.7 Hz, 1H), 7.00 (t, *J* = 7.6 Hz, 1H), 2.22 (s, 3H), 1.74 (s, 6H). LCMS (ESI+) m/z calculated for $C11H13O3^+$ $[M+H]^+$ = 193.1, found = 193.1.

Compound S2:

Bromination: Compound **S1** (2.00 g, 10.41 mmol), *N*-bromosuccinimide (1.94 g, 10.93 mmol, 1.05 equiv.), AIBN (0.171 g, 1.04 mmol, 0.1 equiv.) were mixed in chloroform (110 mL). After the solution was refluxed and stirred for 16 h, the solvent was allowed to cool to r.t and then filtered. The solvent was then removed, and the crude product was used without further purification.

Azidation: Crude benzyl bromide from above (1.00 g, 3.69 mmol), Sodium azide (0.72 g, 11.07 mmol, 3.0 equiv.), were stirred in 40 mL DMF at room temperature, tetrabutylammonium iodide (0.14 g, 0.37 mmol, 0.1 equiv) was added into the reaction flask, and stirred for 16 h. 120 mL water was added into the reaction flask and extracted with EtOAc (100 mL x 3). The extracts were washed with water (100 mL) and brine (100 mL), dried (Na_2SO_4), and concentrated to leave the crude product. The crude product was used without further purification.

Compound S3:

Staudinger: **S2** (311 mg,1.34 mmol) was dissolved in (9:1) THF:H_2O (10 mL). Triphenylphosphine (386mg,1.47mmol, 1.1 equiv.) was then added to into the flask. The mixture was stirred at room temperature for 3 h. The reaction was monitored by TLC and LCMS. The reaction mixture was carried forward directly without any manipulation.

Fmoc protection: A 10% Na_2CO_3 solution (8 mL) was added to the above reaction mixture, followed by adding FmocCl (277 mg, 1.07 mmol, 0.9 equiv.) to the reaction mixture. After 16 h at room temperature, the reaction was concentrated in vacuo. EtOAc (100 mL x 3) was used to extract three times. The extracts were washed with water and brine, dried (Na_2SO_4), and concentrated to leave the crude product. The crude mixture was further purified by silica gel column chromatograph using 10% EtOAc/hexanes to obtain compound **S3** (316 mg) in 55% yield over 4 steps. 1H NMR (500 MHz, cdcl3) δ 7.90 (dd, *J* = 7.8, 1.7 Hz, 1H), 7.76 (d, *J* = 7.6 Hz, 1H), 7.57 (d, *J* = 7.5 Hz, 2H), 7.52 – 7.44 (m, 1H), 7.40 (tt, *J* = 7.5, 0.9 Hz, 2H), 7.33 – 7.26 (m, 2H), 7.08 (t, *J* = 7.7 Hz, 1H), 5.12 (t, *J* = 6.3 Hz, 1H), 4.47 (d, *J* = 6.6 Hz, 2H), 4.36 (d, *J* = 6.3 Hz, 2H), 4.21 (t, *J* = 6.5 Hz, 1H). ^{13}C NMR (126 MHz, cdcl$_3$) δ 161.0, 156.5, 154.0, 143.9, 141.5, 135.9, 129.1, 127.90, 127.2, 127.1, 125.0, 122.6, 120.2, 113.8,

106.6, 77.4, 77.2, 76.9, 66.8, 47.4, 39.5, 26.0. HRMS (ESI+) m/z calculated for C26H24NO5$^+$ [M+H]$^+$ = 430.1649, found = 430.1652.

Compound 1: Acetonide **S3** (500 mg, 1.16 mmol) suspended in 9:1 TFA/H_2O (30 mL) at room temperature. The mixture was stirred at room temperature for 3 h at which point the mixture had become homogeneous. the reaction was concentrated in vacuo.The crude material was dissolved in EtOAC (50 mL) and washed with H_2O (100 mL). EtOAc (100 mL x 3) was used to extract three times. The extracts were washed with water (100 mL) and brine (100 mL), dried (Na_2SO_4), and concentrated to afford **1** (447 mg) as an off-white solid in a 99% yield. 1H NMR (500 MHz, dmso) δ 11.71 (s, 1H), 7.90 (d, *J* = 7.5 Hz, 2H), 7.76 – 7.66 (m, 4H), 7.42 (t, *J* = 7.4 Hz, 2H), 7.37 – 7.28 (m, 3H), 6.89 (t, *J* = 7.7 Hz, 1H), 4.36 (d, *J* = 6.9 Hz, 2H), 4.26 – 4.16 (m, 3H). ^{13}C NMR (126 MHz, dmso) δ 172.4, 158.7, 156.4, 143.9, 140.8, 133.3, 128.6, 127.6, 127.1, 127.0, 125.2, 120.1, 118.6, 118.3, 112.2, 65.3, 46.8, 40.0, 39.9, 39.9, 39.8, 39.7, 39.5, 39.4, 39.2, 39.0, 38.3. HRMS (ESI+) m/z calculated for C23H20NO5+ [M+H]$^+$ = 390.1336, found = 390.1351.

Scheme S2. Synthesis of building block 3.

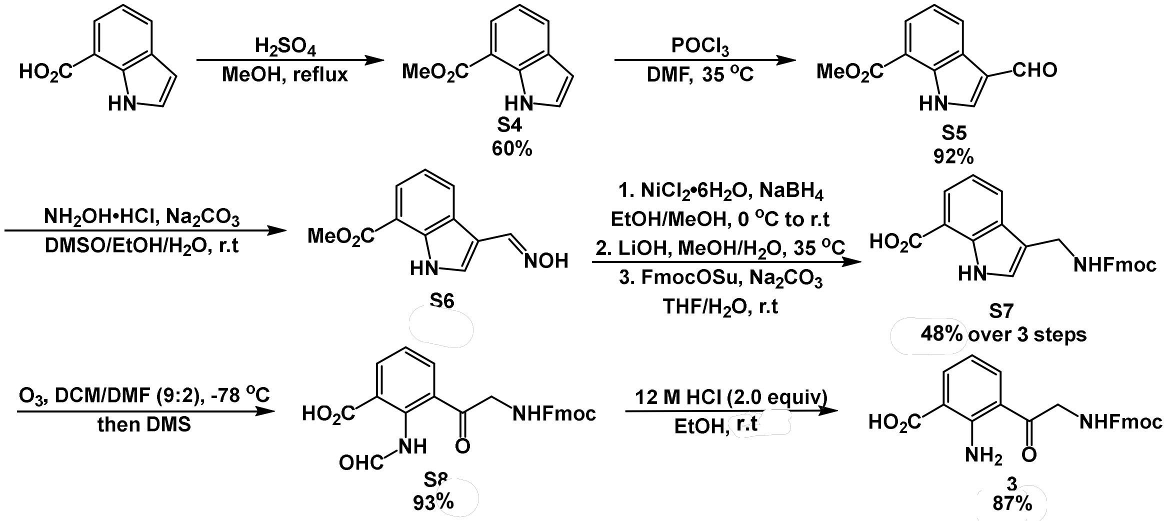

Compound S4 and **S5:** Compounds **S4**, **S5** were made following Fisher and Vilsmeier-Haack formylation formations protocols as previously described.[40,41]

Compound S6 was made following slightly modified literature conditions[40,41]

<u>Oxime formation:</u> A homogeneous solution of **S5** (5.06 g, 24.90 mmol) in DMSO (5 mL)/EtOH (50 mL)//H2O (50 mL) was treated NH_2OH•HCl (2.93 g, 42.33 mmol, 1.7 equiv.) followed by solid Na_2CO_3 (1.83 g, 17.43 mmol, 0.7 equiv.) at room temperature.The following reaction mixture was stirred at room temperature for 2 h before all the aldehyde starting was consumed. The volatiles were removed and then the reaction mixture was then diluted with EtOAc (400 mL) and H_2O (400 mL). The layers were separated, and the aqueous layer was extracted with EtOAc (3 x 100 mL). The organic extracts were pooled and washed with brine and then drained. The organics were dried (Na_2SO_4) and evaporated in vacuo to give **S6** (4.52 g) as a white solid obtained in an 83% yield which was used without any further purification.

Compound S7:

<u>Oxime reduction</u>: A suspension of oxime **S6** (4.52 g, 20.71 mmol) and $NiCl_2$•6H_2O (3.20g, 24.85 mmol, 1.2 equiv.) in 3:2 EtOH/MeOH (207 mL) was sonicated until the reaction mixture became homogeneous. The reaction mixture, equipped with an internal thermometer and was then placed in an ice bath and allowed to cool to 0 °C. $NaBH_4$ (3.09 g, 82.84 mmol, 4 equiv.) was added to the above mixture in small portions (over 1 h) such that the internal temperature of the reaction did not exceed 4 °C (Note: the indole is reduced to the indoline when

temperature are not carefully controlled). After the final addition of $NaBH_4$, the reaction mixture was allowed to stir for 1 h at 0 °C and then was removed from the ice bath and allowed to slowly warm to room temperature. After the full consumption of the oxime (ca. 1 h) was tracked by TLC the reaction filtered through a pad of celite. The filtrate was quenched with a solution of $NH_4OH_{(aq)}$ (200 mL) and then the EtOH/MeOH was carefully removed on the rotovap (no heating bath). The aqueous solution was extracted with EtOAc (3 x 300 mL). The organic extracts were then washed with 9:1 brine/ NH_4OH (400 mL) and then dried (Na_2SO_4) and evaporated in vacuo to give the corresponding crude amine (3.77 g) as a yellow oil which was carried forward without any further purification.

Ester hydrolysis: The crude indolic amine (3. 77 g, 18.46 mmol) was taken up in an 85:15 solution of MeOH/H_2O (120 mL) and then treated with 1 M $LiOH_{(aq)}$ (37 mL, 37 mmol, 2.0 equiv.) followed by solid LiOH•H_2O (1.55 g, 37 mmol, 2.0 equiv.). The reaction mixture was heated to 35 °C and stirred overnight. After overnight stirring the reaction mixture was cooled and placed on the rotovap to remove the MeOH. Then, the basic reaction mixture was cooled to 0 °C, diluted with THF (30 mL) and carefully neutralized with AcOH (3.7 mL, ca. 3.5 equiv.). This solution was carried forward directly.

Fmoc protection: The above mixture was treated with 10% $Na_2CO_{3\,(aq)}$ (12 mL) followed by a solution of FmocOSu (6.23 g, 18.46 mmol, 1.0 equiv.) in THF (50 mL). The reaction mixture was removed from the ice bath and allowed to stir at room temperature overnight. After overnight stirring, the reaction was diluted with EtOAc (400 mL) and acidified with 1 M HCl (200 mL, ca. pH 2). The layers were separated and the aqueous extracted with EtOAc (3 x 100 mL). The organic extracts were pooled and washed with brine (200 mL) and then dried (Na_2SO_4) and evaporated in vacuo to give the crude Fmoc protected product. The crude product was dissolved in hot 2:1 EtOH/H_2O (300 mL) and left to cool to room temperature. The resulting precipitate was collected by vacuum filtration and the solid was rinsed with cold 1:1:4 MeOH/EtOH/H_2O (3x) and then once with 1:1 DCM/hexanes. The solid was transferred to a flask and azeotroped with MeCN to produce **S7** (3.55 g) as a fluffy white solid in a 47% yield over 3 steps. HRMS (ESI-) m/z calculated for C25H19N2O4- $[M-H]^-$ = 411.1350, found = 411.1352.

Compound S8:

Ozonolysis: In a 2-neck flask, a solution of **S7** (3.71 g, 8.99 mmol) in DMF (24 mL) was slowly treated with DCM (132 mL). This homogeneous solution was cooled to -78 °C and then was treated with a stream of O_3/O_2 (1.2 atm O_2 pressure, 100% ozone) The reaction mixture was buvvled with ozone for 20 min. After, the ozone generator was turned off and the solution was bubbled with O2 for 5 min. Then, the reaction mxiutre was slowly treated with DMS (6.6 mL, 89.90 mmol, 10 equiv.). The mixture was removed from cooling and allowed to stir at room temperature for 1 h. The DCM was then removed in vacuo and the mixture was diluted with H_2O (200 mL). EtOAc (300 mL) was added and the two layers were separated. The aqueous was extracted with EtOAc (3x200 mL). The organic extracts were then washed with brine (4x200mL), dried (Na_2SO_4), filted and evaporated to give **S8** (3.91 g) as an yellow solid in a 98% yield. 1H NMR (400 MHz, dmso) δ 13.37 (s, 1H), 10.60 (s, 1H), 8.26 (s, 1H), 8.03 (d, *J* = 7.8 Hz, 1H), 7.91 – 7.85 (m, 3H), 7.74 – 7.67 (m, 3H), 7.45 – 7.36 (m, 3H), 7.36 – 7.29 (m, 2H), 4.42 – 4.19 (m, 5H). ^{13}C NMR (101 MHz, dmso) δ 198.2, 167.7, 161.0, 156.5, 143.9, 140.8, 133.2, 132.8, 131.5, 127.7, 127.1, 125.3, 125.2, 125.0, 120.1, 65.9, 48.4, 46.7, 40.2, 39.9, 39.7, 39.5, 39.3, 39.1, 38.9. HRMS (ESI+) m/z calculated for C25H21N2O6$^+$ $[M+H]^+$ = 445.1394, found = 445.1400.

Compound 3: A suspension of **S8** (3.75 g, 8.44 mmol) in 95% EtOH (110 mL) was treated with 12 M HCl (1.40 mL, 2.0 equiv., 16.88 mmol). The resulting suspension was stirred at room temperature for 20 h. The now homogeneous reaction mixture was evaporated in vacuo. The crude solid was suspended in 1:1 EtOH/H_2O (150 mL). The mixture was heated to boil. The suspension was allowed to cool to room temperature and stand for 3 h. The mixture was further chilled to -20 °C and the the solid was collected by filtration and the solid rinsed with cold 1:4 MeOH/H_2O and allowed to dry. The solid was transferred to a flask and azeotroped with MeCN (3x) to give **3** (3.05 g) as a pale-yellow solid obtained in a 87% yield. ^1H NMR (400 MHz, dmso) δ 12.96 (s, 1H), 8.57

(s, 2H), 8.18 – 8.02 (m, 2H), 7.90 (d, J = 7.5 Hz, 2H), 7.75 (d, J = 7.5 Hz, 2H), 7.56 (t, J = 6.0 Hz, 1H), 7.39 (dt, J = 32.0, 7.5 Hz, 4H), 6.63 (t, J = 7.9 Hz, 1H), 4.49 (d, J = 6.0 Hz, 2H), 4.37 – 4.18 (m, 3H). ^{13}C NMR (101 MHz, dmso) δ 196.9, 169.1, 156.7, 152.3, 143.9, 140.7, 138.1, 136.8, 127.6, 127.1, 125.3, 120.1, 116.5, 113.4, 112.2, 65.7, 47.5, 46.7. HRMS (ESI-) m/z calculated for C24H19N2O5- [M-H]$^-$ = 415.1299, found = 415.1300.

Scheme S3. Attempted synthesis of building block 2.
The synthesis of **2** proved to be problematic and not suitable for further scale up reactions. a) We found that the reduction of the NO_2 with H_2/Pd resulted in unknown byproducts that could not be easily separated from the desired aniline. b) We found that the reduction of the NO_2 with Zn resulted in a somewhat cleaner reaction, however hydrolysis of the resulting ester was sluggish and required harsh reaction conditions (35 equiv. of LiOH). The Fmoc protection could be achieved but building block **2** could not be separated from TPPO and the overall reaction yield was unacceptable (<10%).

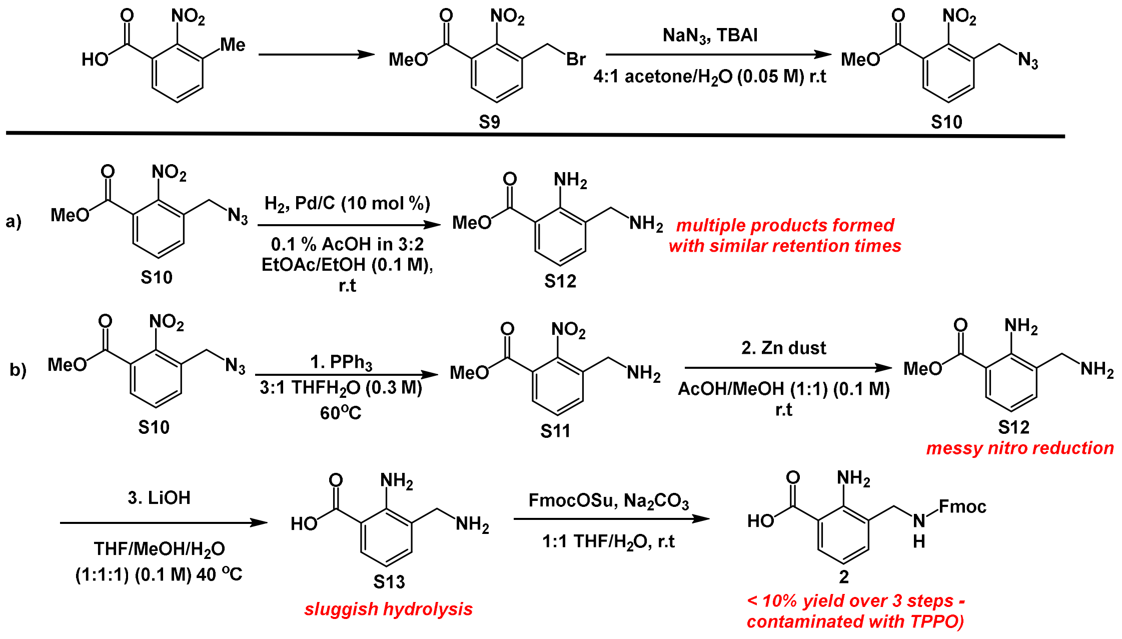

Compound S10: benzyl bromide **S9**[42] (1.2 g, 4.4 mmol) in 4:1 THF/H_2O (0.05 M) was treated with TBAI (0.1 equiv.) followed by NaN_3 (1.5 equiv.). The resulting mixture was stirred for 18 h. Afterwards, the acetone was removed, and the aqueous phase was extracted with EtOAc (3x). The extracts we wash with brine (2x) and the dried (Na_2SO_4), filtered and evaporated in vacuo. The crude material was then purified by FC (10% EtOAc/hexanes) to give **S10** as a yellow oil (0.9 g, 87% yield). ^{1}H NMR (500 MHz, dmso) δ 8.00 (dd, J = 7.7, 1.5 Hz, 1H), 7.93 (dd, J = 7.8, 1.5 Hz, 1H), 7.81 (t, J = 7.8 Hz, 1H), 4.60 (s, 2H), 3.85 (s, 3H). ^{13}C NMR (126 MHz, dmso) δ 176.2, 163.6, 148.6, 135.0, 131.9, 130.8, 128.9, 123.9, 53.3, 49.0. LCMS (ESI+) m/z calculated for C9H9N4O4+ [M+H]+ = 237.1, found = 237.1.

<u>Routes a) and b):</u> *These routes were attempted using 0.5 mmol **S10**. NMR spectra for isolated material can be found below.*

Figure S1:1H-NMR Compound **S3** (500 MHz, DMSO-*d*6)

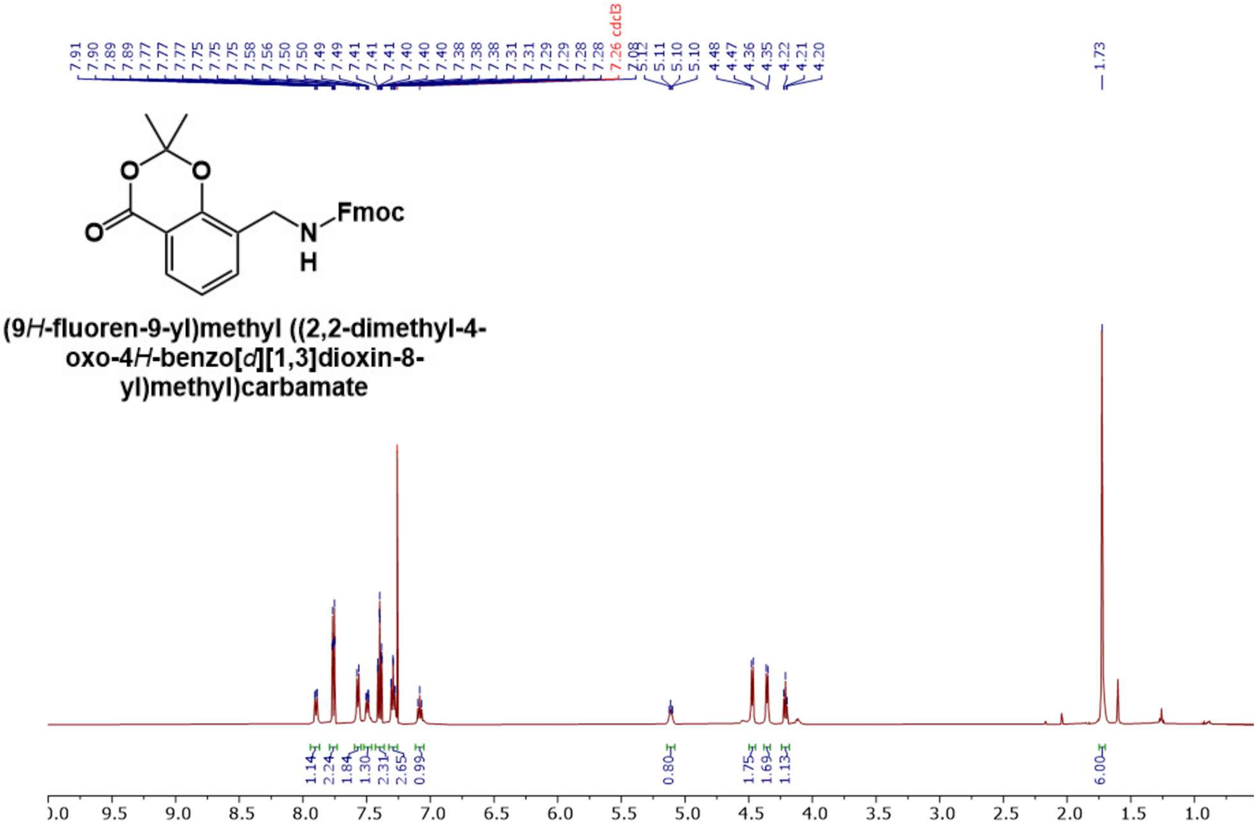

Figure S2: 13C-NMR Compound **S3** (126 MHz, DMSO-*d*6)

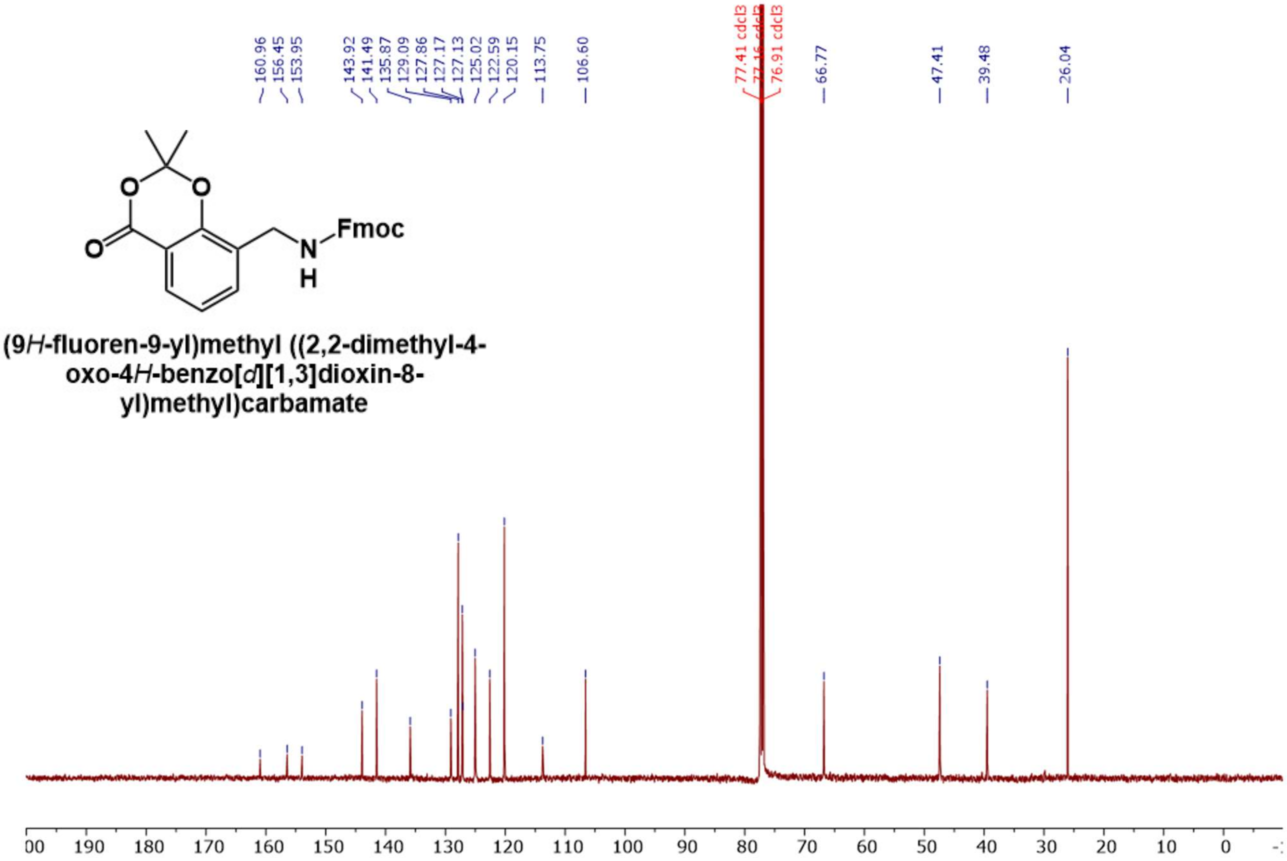

Figure S3: 1H-NMR Compound **1** (500 MHz, DMSO-*d*6)

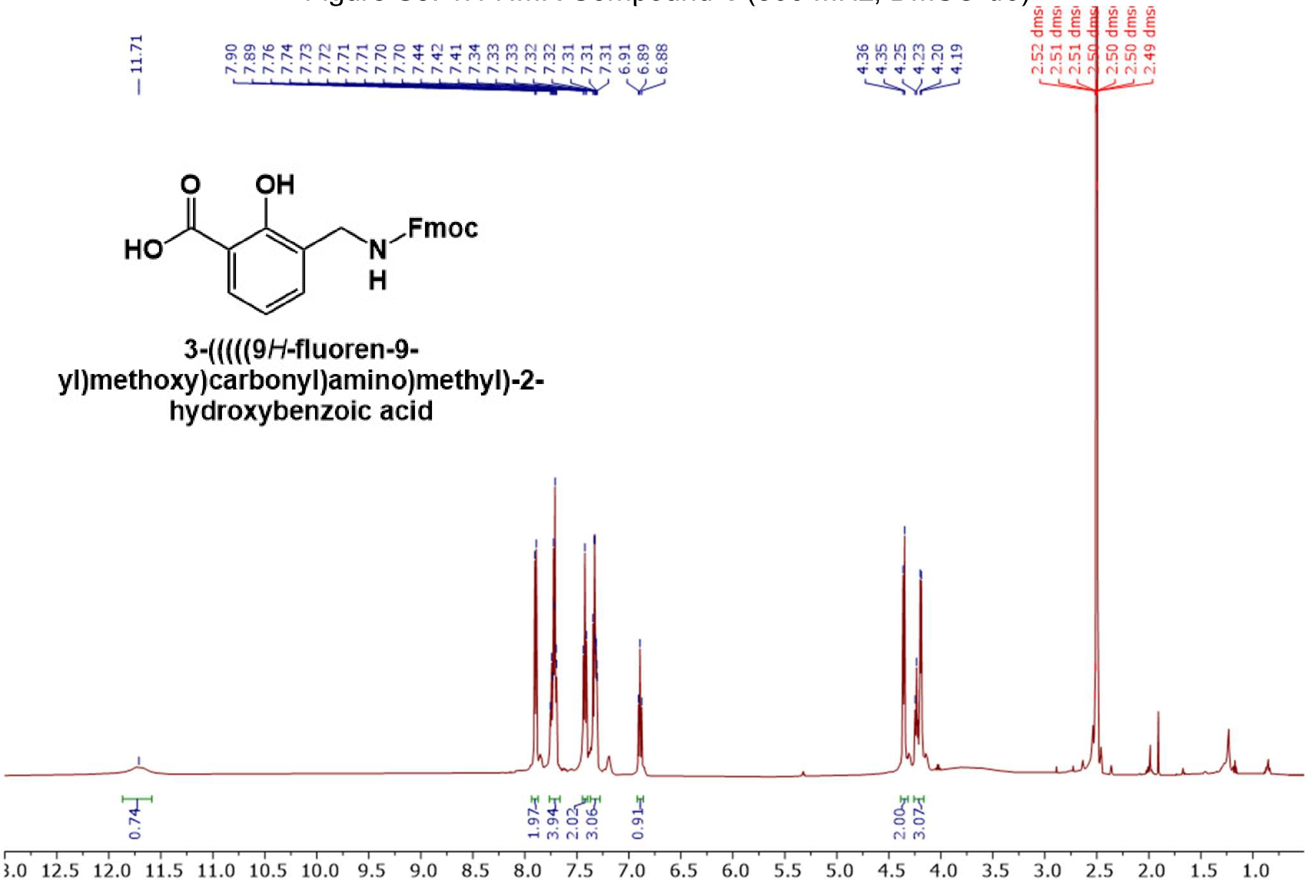

Figure S4: 13C-NMR Compound **1** (126 MHz, DMSO-*d*6)

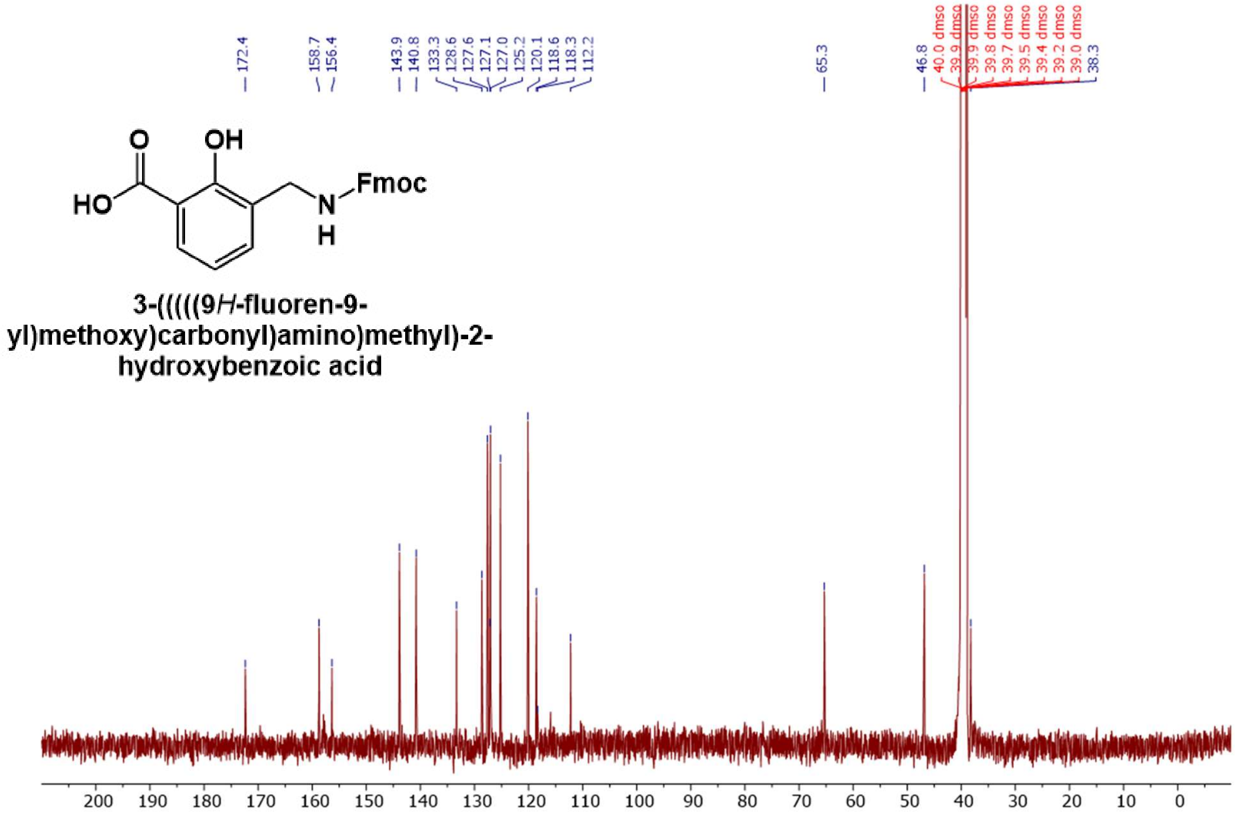

Figure S5: 1H-NMR Compound **S7** (400 MHz, DMSO-*d*6)

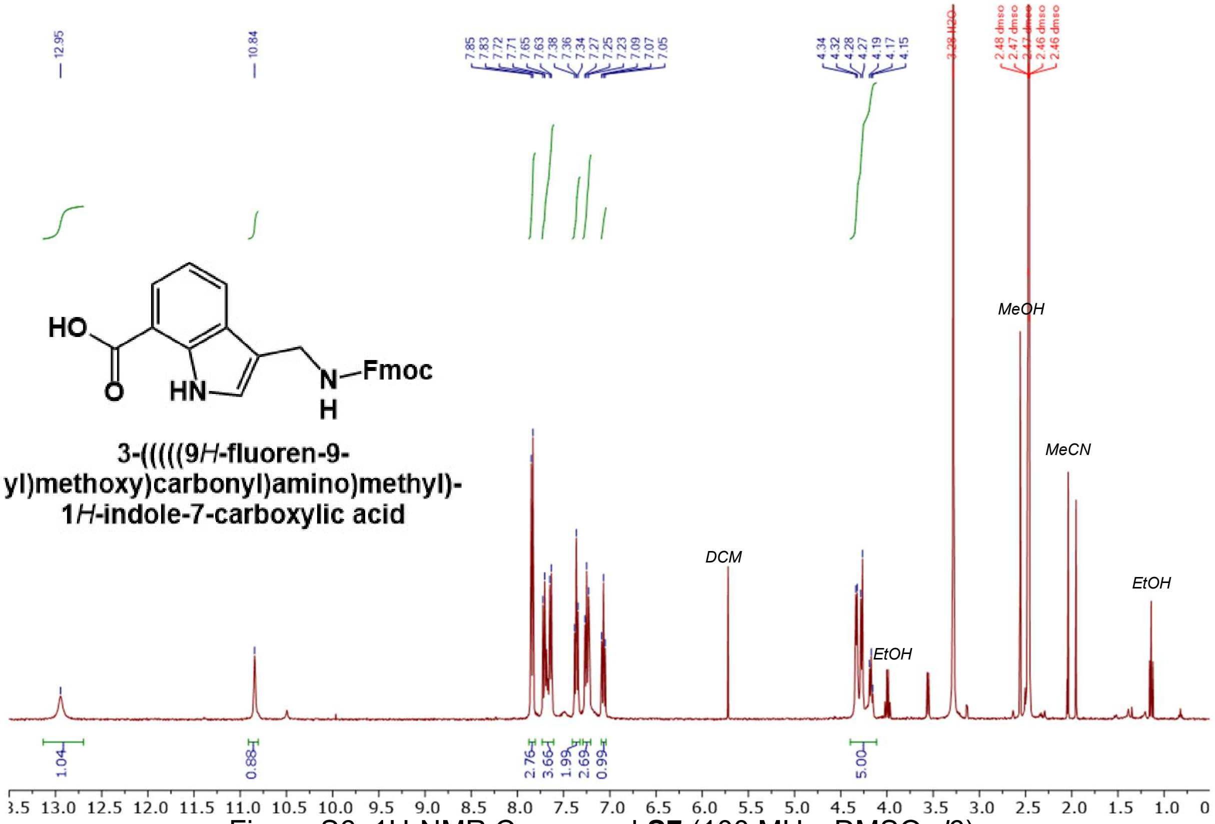

Figure S6: 1H-NMR Compound **S7** (100 MHz, DMSO-*d*6)

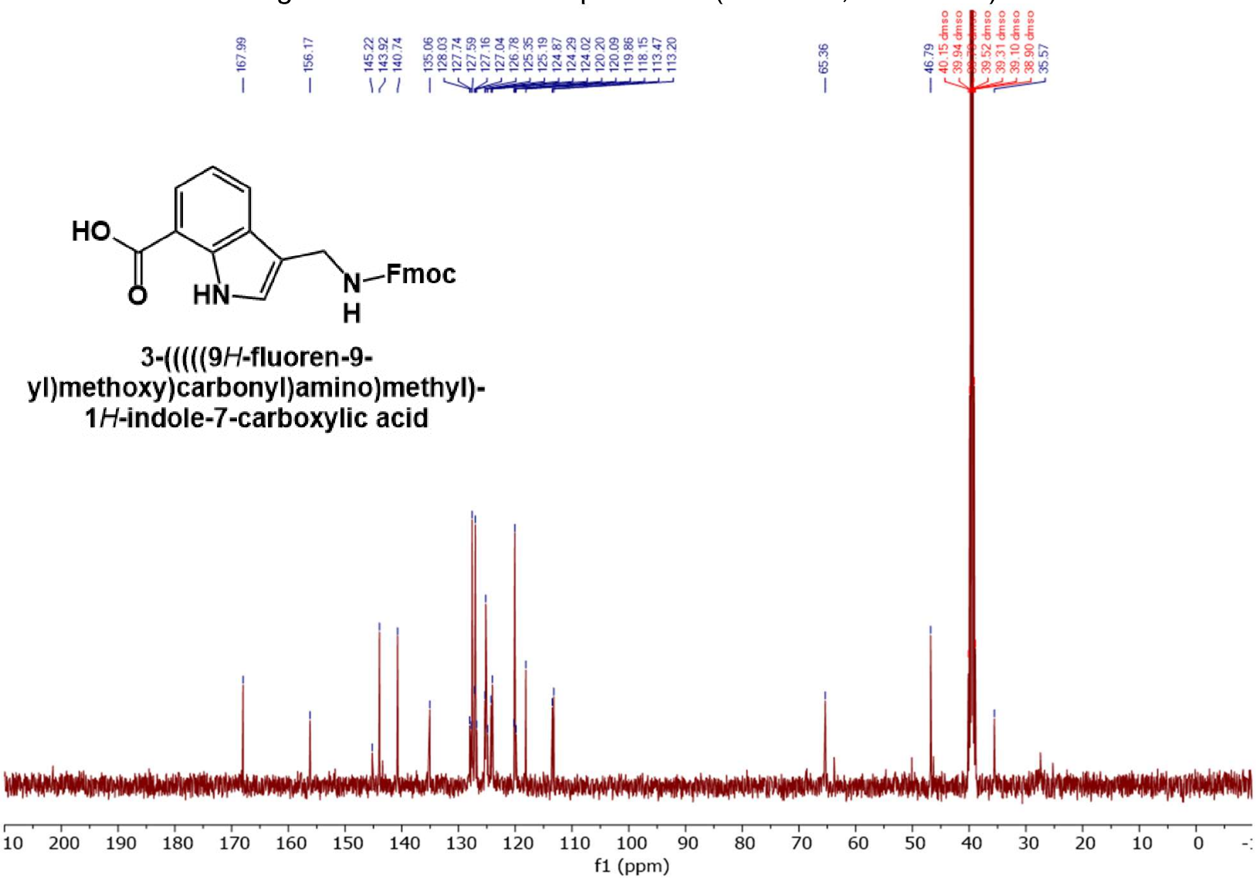

Figure S7: 1H-NMR Compound **S8** (400 MHz, DMSO-*d*6)

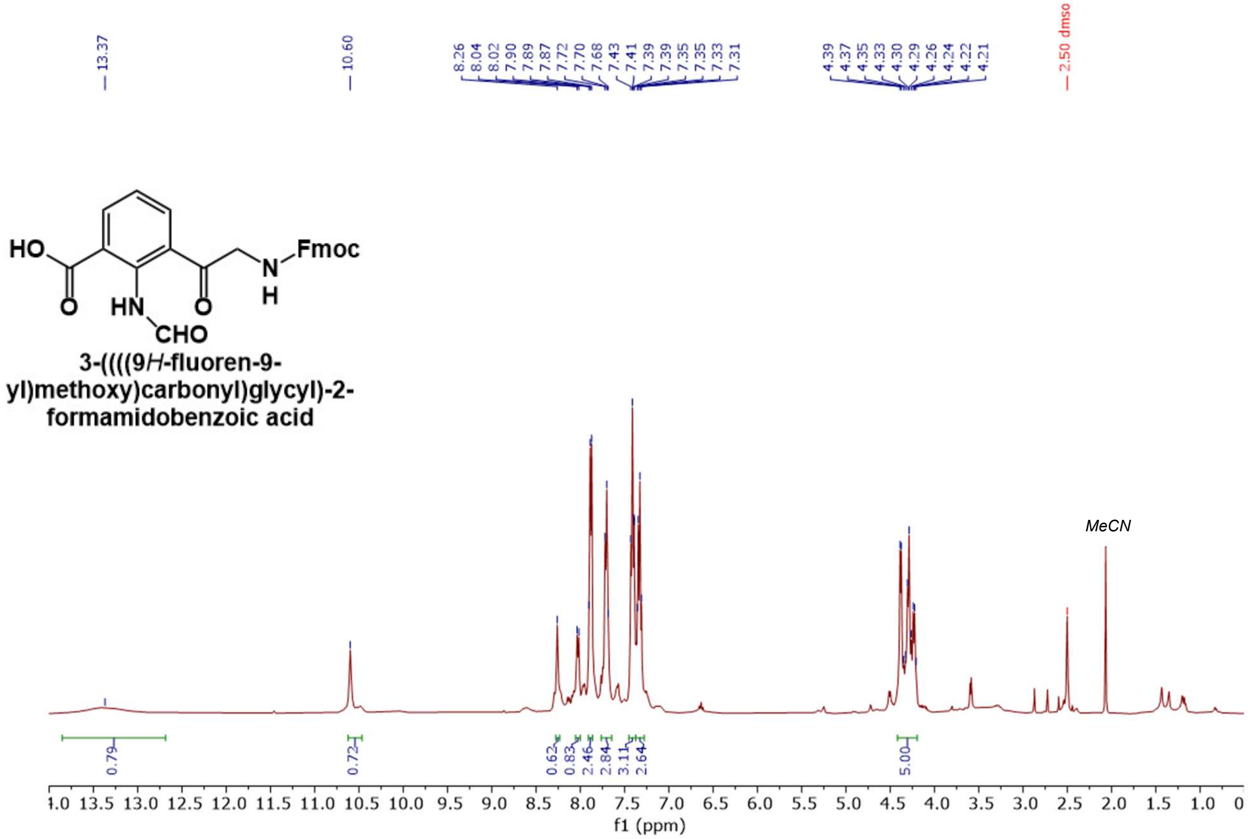

Figure S8: 1H-NMR Compound **S8** (100 MHz, DMSO-*d*6)

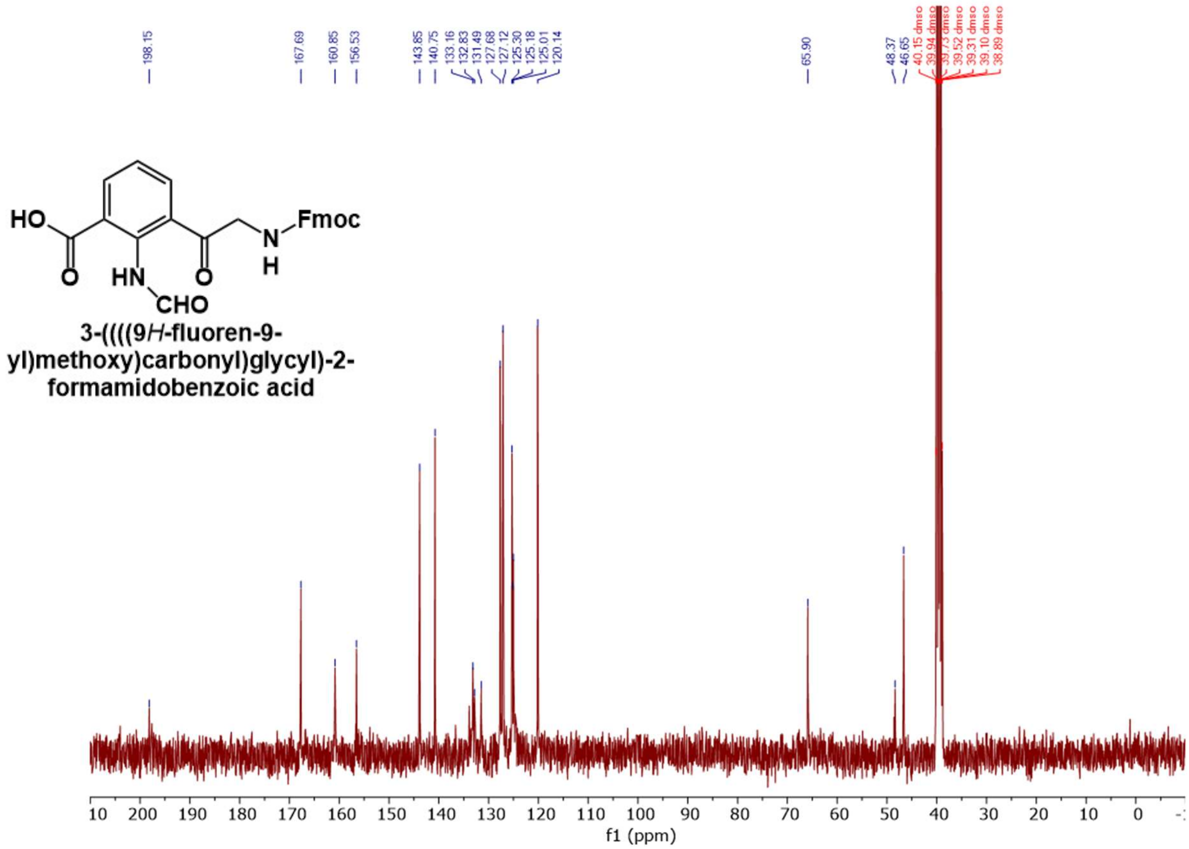

Figure S9: 1H-NMR Compound **3** (400 MHz, DMSO-*d*6)

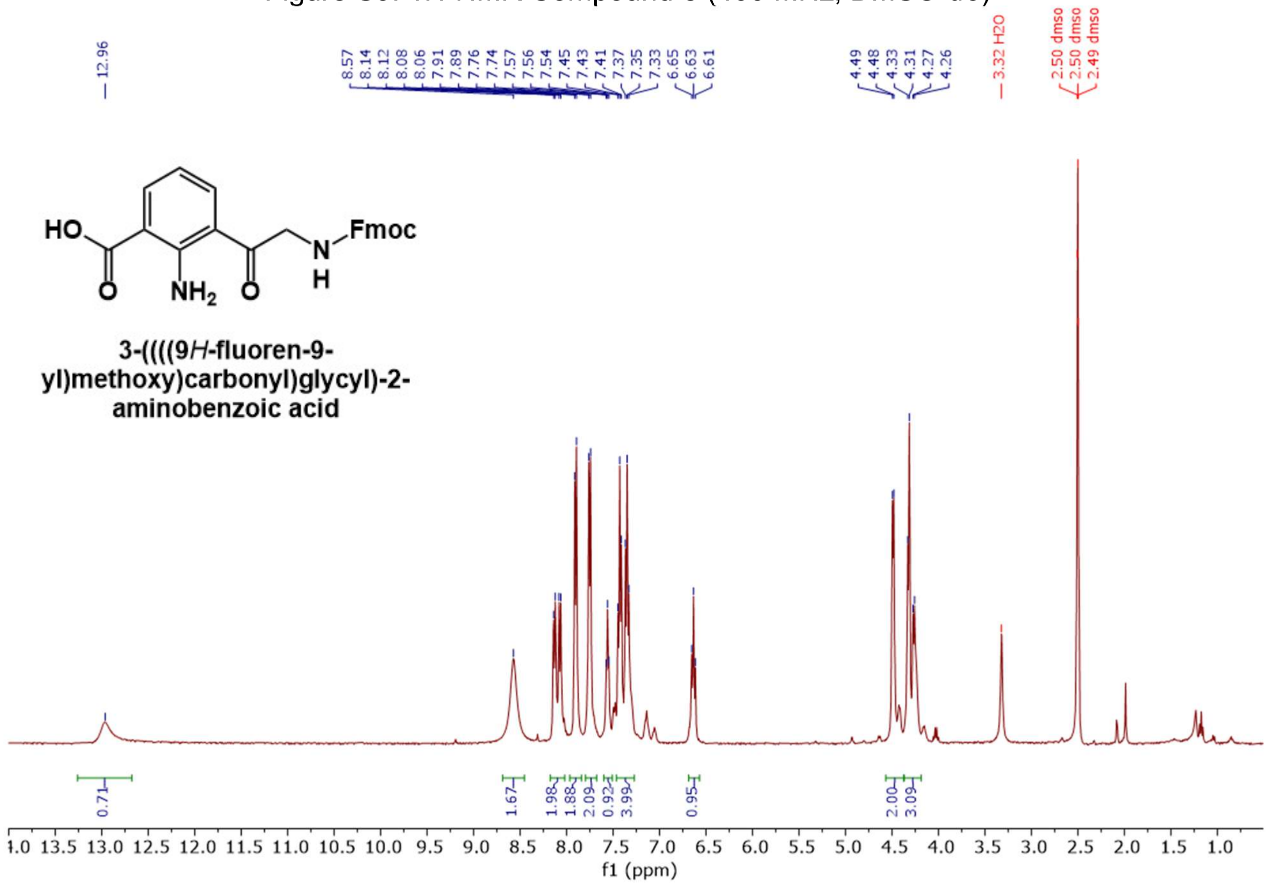

Figure S10: 1H-NMR Compound **3** (100 MHz, DMSO-*d*6)

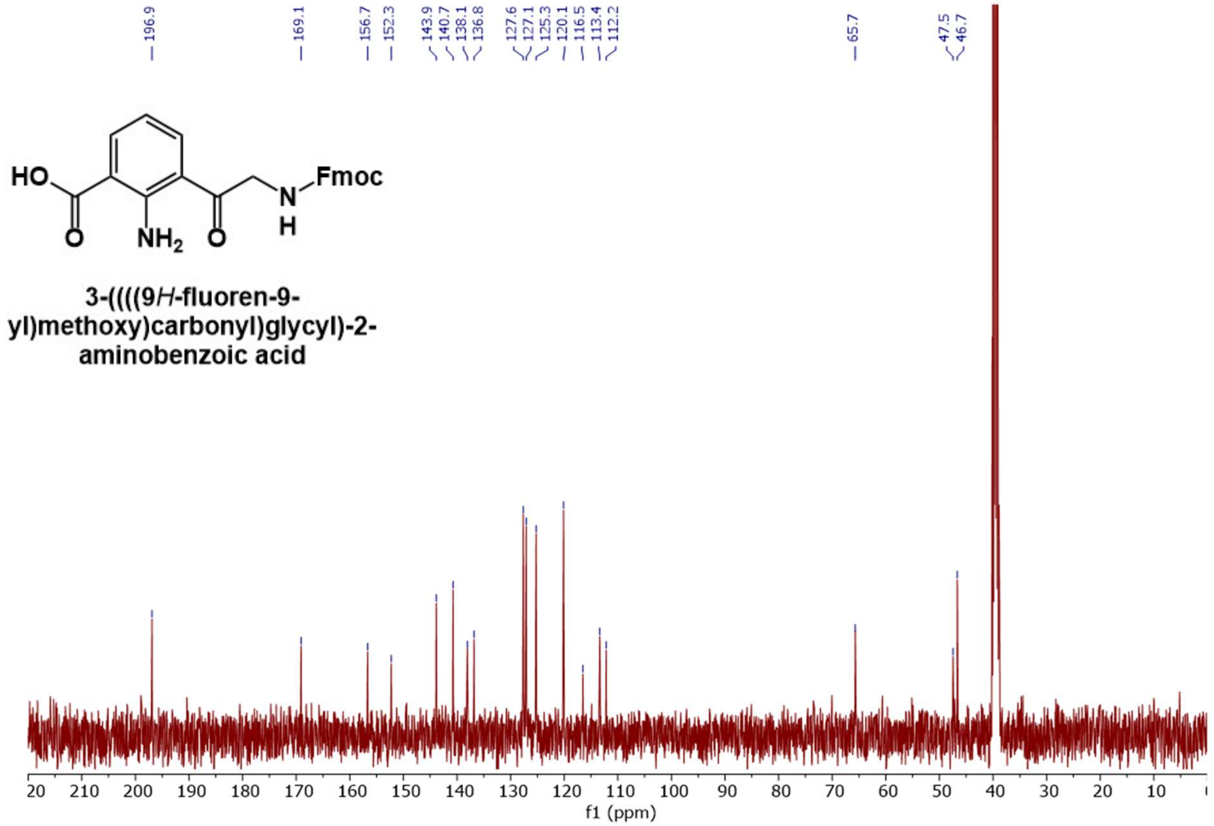

Figure S11: 1H-NMR Compound **S10** (500 MHz, DMSO-*d*6)

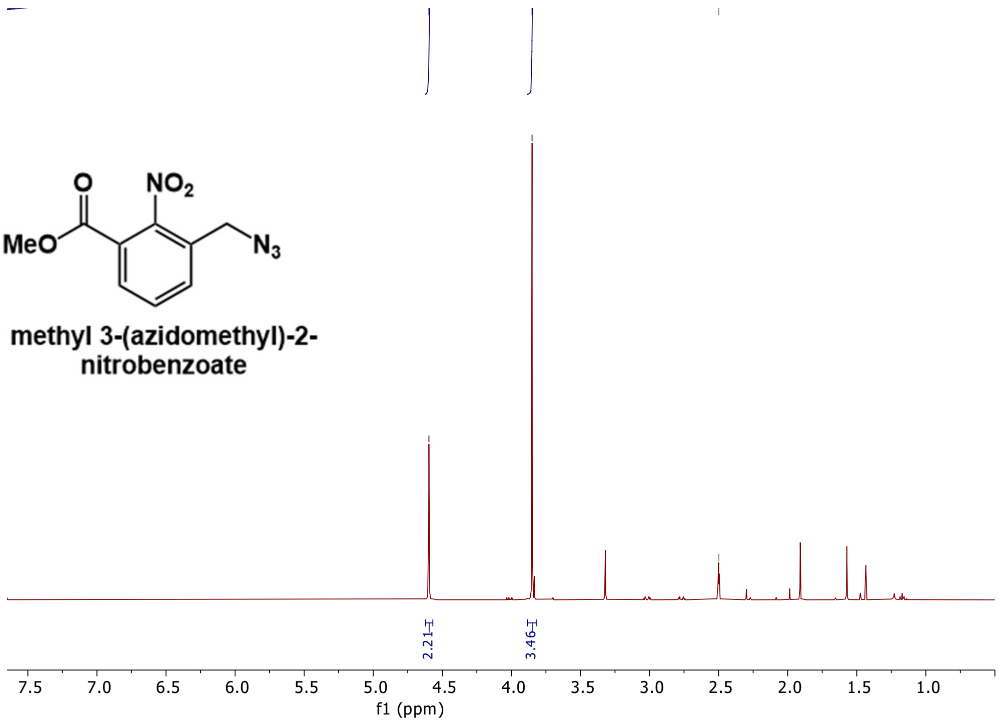

Figure S12: 13C-NMR Compound **S10** (125 MHz, DMSO-*d*6)

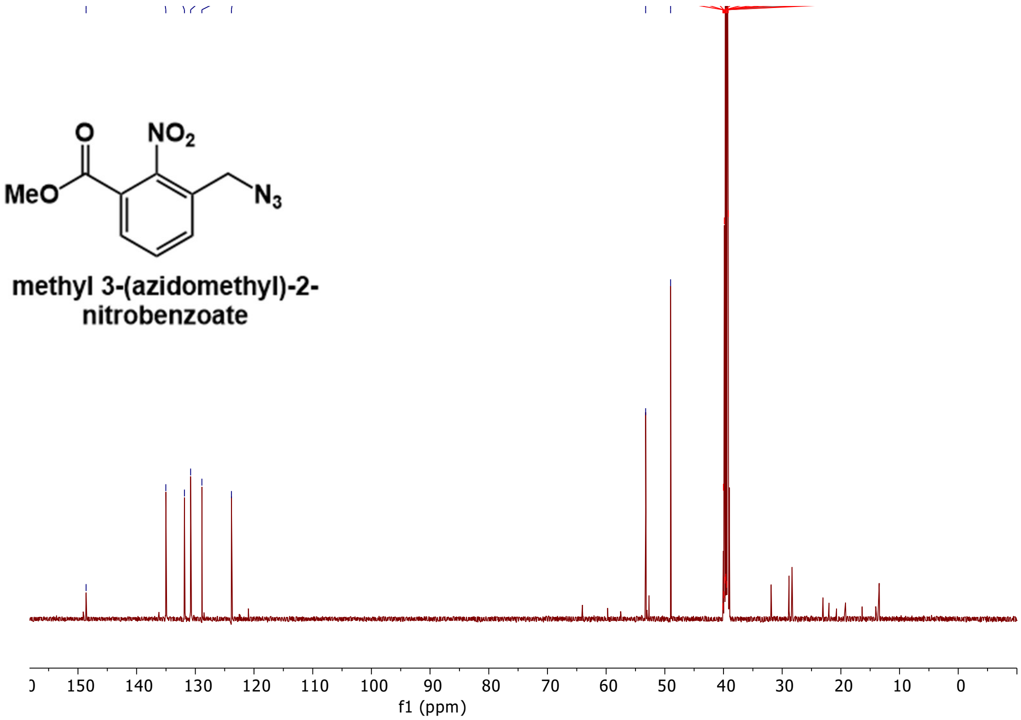

Figure S13: 1H-NMR Crude compound **S11** (500 MHz, DMSO-*d*6)

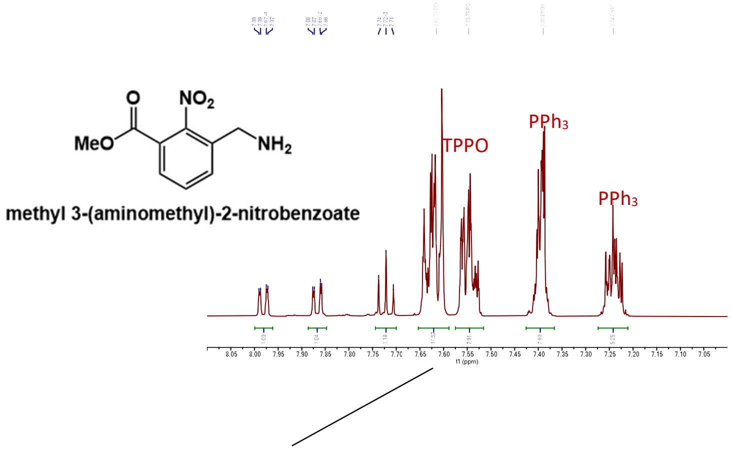

Figure S14: 13C-NMR Crude compound **S11** (125 MHz, DMSO-*d*6)

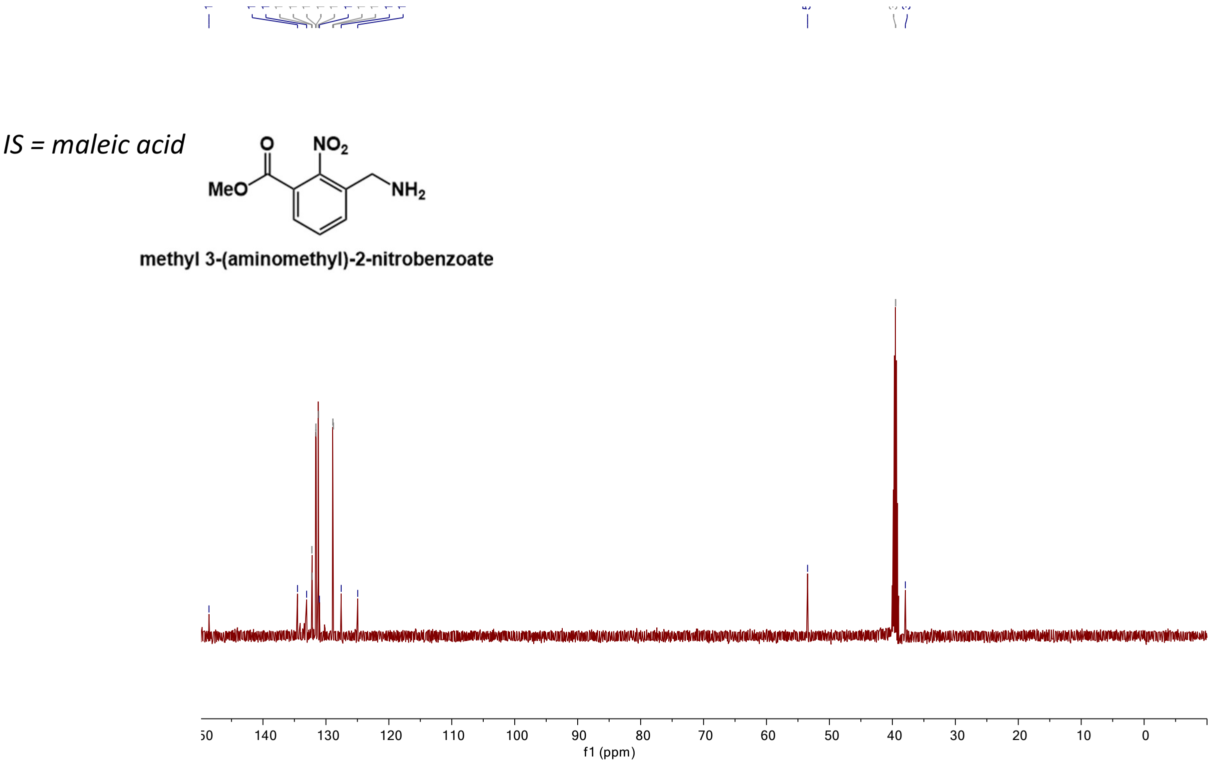

Figure S15: 1H-NMR Crude compound **2** (500 MHz, DMSO-*d*6)

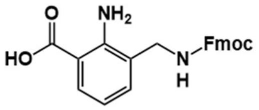

3-(((((9*H*-fluoren-9-yl)methoxy)carbonyl)amino)methyl)-2-aminobenzoic acid

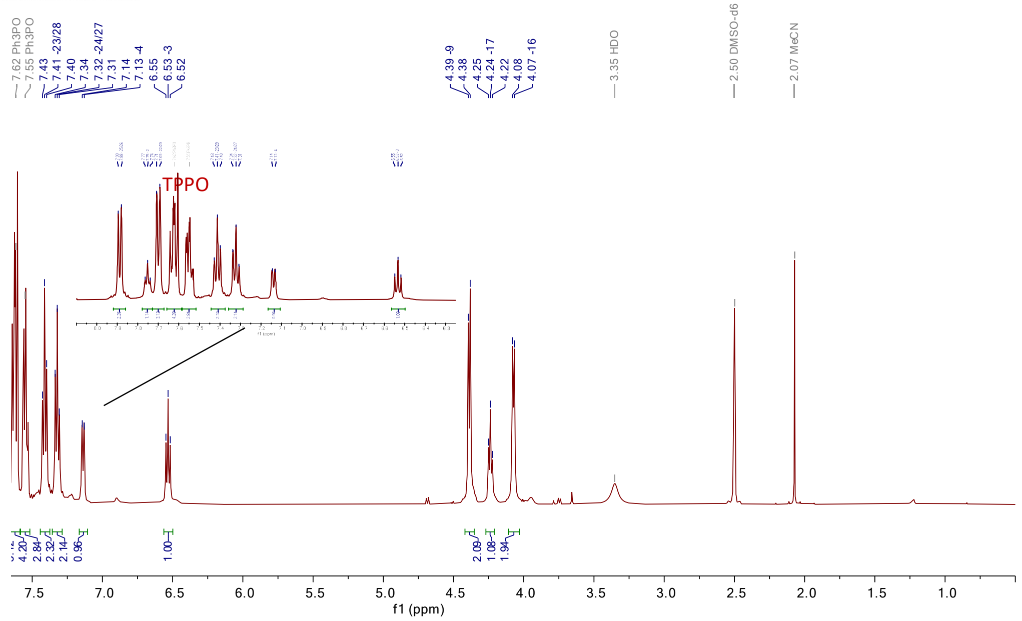

Figure S16: 13C-NMR Crude compound **2** (125 MHz, DMSO-*d*6)

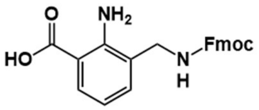

3-(((((9*H*-fluoren-9-yl)methoxy)carbonyl)amino)methyl)-2-aminobenzoic acid

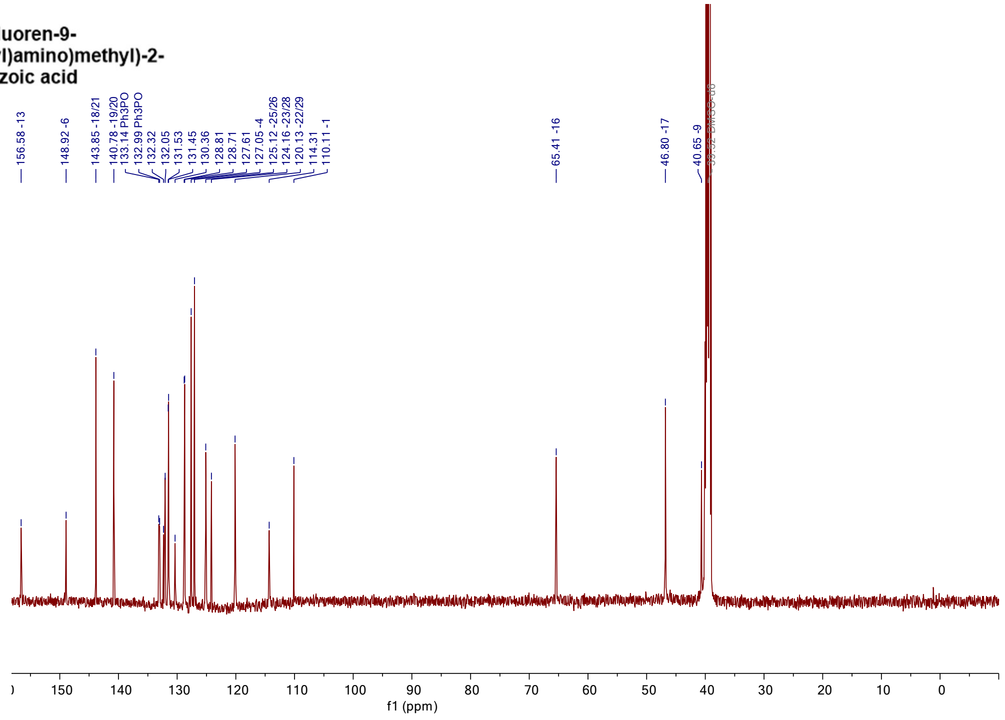

Solid-Phase Peptide Synthesis

General Procedure for Resin Loading and Fmoc SPPS (linear peptides S13-20)

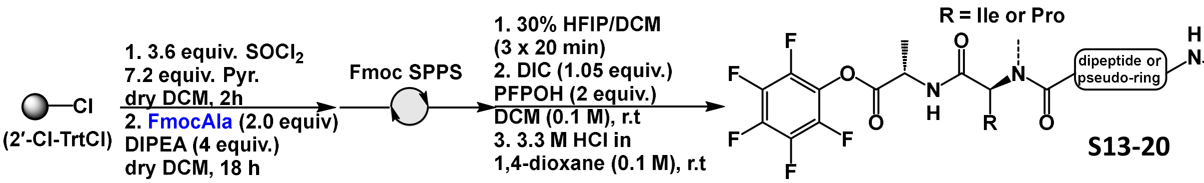

The SPPS synthesis was performed manually beginning from 2′-Cl-TrtCl polystyrene resin (theoretical substitution = 1.1 mmol/g, 90.9 mg, 0.1 mmol, 1 equiv). The 2′-Cl-TrtCl polystyrene resin was preactivated in dry DCM (20 mL) with $SOCl_2$ (13 µL, 3.6 equiv) and pyridine (29 µL, 7.2 equiv) under reflux for 2 h. The resin was then transferred to a disposable peptide cartridge and rinsed with dry DCM, followed by loading with FmocAlaOH (2.0 equiv) and DIPEA (4.0 equiv) in dry DCM (1.0 mL) (18 h). The resin was capped with 17:2:1 DCM/MeOH/DIPEA (3 × 10 min).

*Synthesis of homodetic peptides **S13-16***

All commercial Fmoc -amino acids and amino acid **S18** (4 equiv) were activated for 5 min using HCTU (4 equiv) and DIPEA (8 equiv) in DMF (1.0 mL) and then coupled for 1 h. All Fmoc groups were removed using 20% pipiridine in DMF (1.0 mL) (2 x 10 min). In between each deprotection and coupling step the resin was rinsed with DMF (3x). The final amino acid, BocGlyOH, was activated for 5 min using HCTU (4 equiv) and DIPEA (8 equiv) in DMF (1.0 mL) and then coupled for 1 h. The global deprotection was executed by treating the resin bound peptide with 30 % hexafluoroisopropanol (HFIP) in DCM (3x20 min). The resin was then rinsed with DCM (2x) and then 1 % MeOH in DCM. The cleavage cocktail was condensed under a stream of N_2 to remove volatiles. The peptide residue was then precipitated with ice cold Et_2O. The Et_2O was carefully removed using a stream of N_2 to afford each linear peptide. The crude linear Boc protected peptides were converted into the pentafluorophenol (PFPOH) ester. This was done by treating a solution of the peptides and PFPOH (2.0 equiv.) in DCM (0.1 M) with DIC (1.05 equiv.). The reactions were monitored until completion. The mixtures were condensed in vacuo and then treated with 3.3 M HCl in 1,4-dioxane (0.1 M) to remove the N-terminal Boc group. The reactions were monitored until completion. The mixtures were condensed in vacuo and then azeotroped with heptane (3x) to afford crude peptides **S13-16** as oily tan residues.

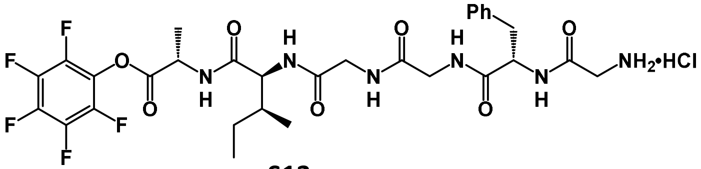

S13

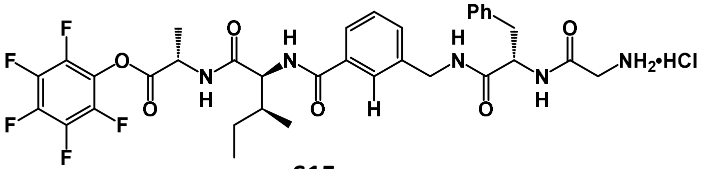

S15

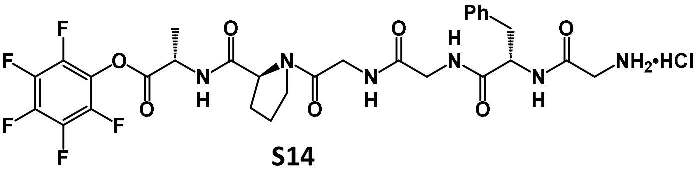

S14

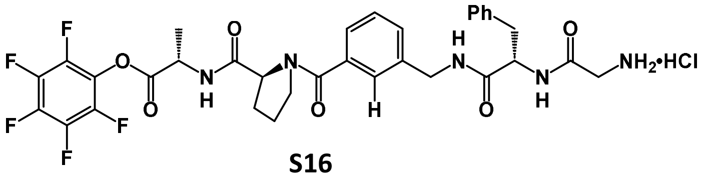

S16

*Synthesis of pseudo-ring peptides **S17-18***

All Fmoc groups were removed using 20% pipiridine in DMF (1.0 mL) (2 x 10 min). The first amino acid after resin loading (either FmocIle or FmocPro) was activated for 5 min using HCTU (4 equiv) and DIPEA (8 equiv) in DMF (1.0 mL) and then coupled for 1 h. Building block **1** was coupled as follows: **1** (1.2 equiv.) and 6Cl-HOBt (4.0 equiv.) was dissolved in DMF (2 mL) and treated with DIC (1.2 equiv.). This mixture was allowed to incubate for 5 min before it was added to the resin. The coupling was left overnight. All subsequent amino acids were coupled as follows: Fmoc/Boc amino acids (4.0 equiv.) and 6Cl-HOBt (5.0 equiv.) were dissolved in DMF (0.1 M) and then treated with DIC (4.0 equiv.) These mixtures were allowed to incubate for 5 min before they were added to the resin. The couplings were left for 3 h. In between each deprotection and coupling step the resin was rinsed with DMF (3x). The global deprotection was executed by treating the resin bound peptide with 30 % hexafluoroisopropanol (HFIP) in DCM (3 x 20 min). The resin was then rinsed with DCM (2x) and then 1% MeOH

in DCM. The cleavage cocktail was condensed under a stream of N_2 to remove volatiles. The peptide residue was then precipitated with ice cold Et_2O. The Et_2O was carefully removed using a stream of N_2 to afford each linear peptide. The crude linear Boc protected peptides were converted into the pentafluorophenol (PFPOH) ester. This was done by treating a solution of the peptides and PFPOH (2.0 equiv.) in DCM (0.1 M) with DIC (1.05 equiv.). The reactions were monitored until completion. The mixtures were condensed in vacuo and then treated with 3.3 M HCl in 1,4-dioxane (0.1 M) to remove the N-terminal Boc group. The reactions were monitored until completion. The mixtures were condensed in vacuo and then azeotroped with heptane (3x) to afford crude peptides **S17-18** as oily tan residues.

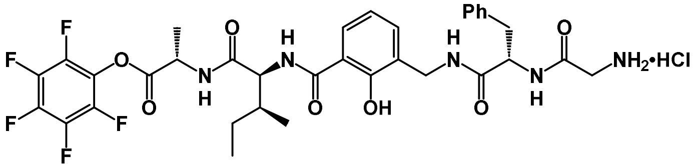

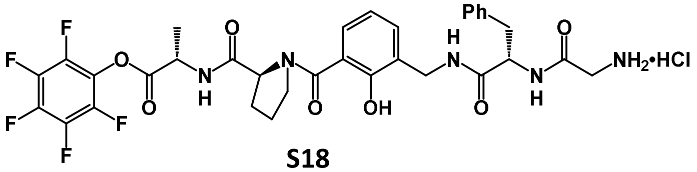

S18

*Synthesis of pseudo-ring peptides **S19-20***

All Fmoc groups were removed using 1% DBU in DMF (2.0 mL) (3x1 min). All commercial (proteinogenic) Fmoc -amino acids (4.0 equiv) were activated for 5 min using HCTU (4 equiv) and DIPEA (8 equiv) in DMF (2.0 mL) and then coupled for 1 h. Building block **3** was coupled as follows: **3** (1.2 equiv.) and HATU (1.2 equiv.) were dissolved in DMF (2 mL) and treated with DIPEA (2.4 equiv.). This mixture was allowed to incubate for 5 min before it was added to the resin. The coupling was left overnight. The final amino acid, BocGlyOH, was activated for 5 min using HCTU (4 equiv) and DIPEA (8 equiv) in DMF (1.0 mL) and then coupled for 1 h. The global deprotection was executed by treating the resin bound peptide with 30% hexafluoroisopropanol (HFIP) in DCM (3 x 20 min). The resin was then rinsed with DCM (2x) and then 1 % MeOH in DCM. The cleavage cocktail was condensed under a stream of N_2 to remove volatiles. The peptide residue was then precipitated with ice cold Et_2O. The Et_2O was carefully removed using a stream of N_2 to afford each linear peptide. The crude linear Boc protected peptides were converted into the pentafluorophenol (PFPOH) ester. This was done by treating a solution of the peptides and PFPOH (2.0 equiv.) in DCM (0.1 M) with DIC (1.05 equiv.). The reactions were monitored until completion. The mixtures were condensed in vacuo and then treated with 3.3 M HCl in 1,4-dioxane (0.1 M) to remove the N-terminal Boc group. The reactions were monitored until completion. The mixtures were condensed in vacuo and then azeotroped with heptane (3x) to afford crude peptides **S19-20** as oily tan residues.

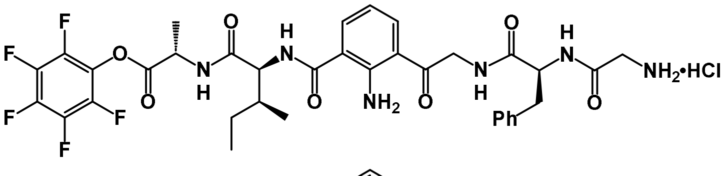

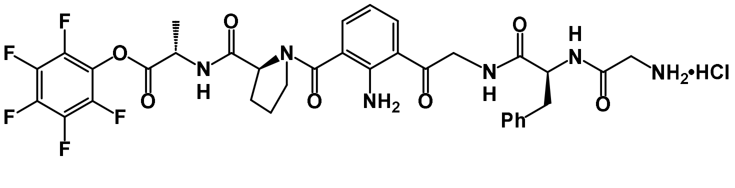

Building block **S21 was made following the protocol outline by Huh et al.[43]*

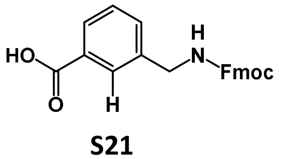

S21

General Procedure for macrocyclization

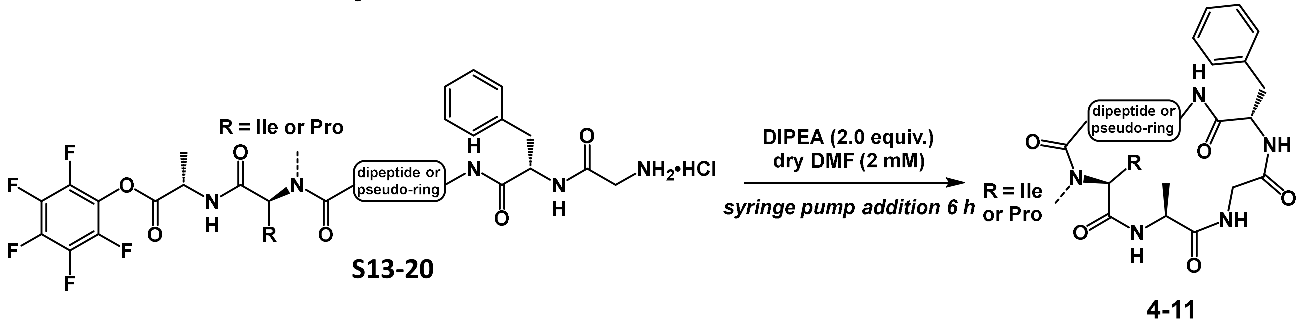

General Procedure for Peptide Macrocyclization Reactions for peptides 4-11

Linear peptide (**S13-20**) was taken up in dry DMF (5 mL). In a separate 100 mL round bottom flask, dry DMF (45 mL) was treated with DIPEA (2.0 equiv.). This solution was then slowly treated with a solution of linear peptide in DMF (syringe pump addition at a rate of 1 mL/h). After the addition of the peptide, the mixture was left to stir for 18 h at room temperature. After 18 h the reaction mixture was evaporated and the crude peptide was purified:

*Purification of peptides **4-7,11***

The crude mixture was dissolved in a 1:1 MeCN/H_2O. The peptide was then purified by C18-reversed-phase chromatography and then peak fractions were pooled and lyophilized to afford the desired peptide **4-7,11** as fluffy white or yellowish solids.

*Purification of peptides **8-10***

The crude mixture was suspended in a 1:1 EtOH/H_2O. The suspension was heated to a boil and then left to cool to room temperate (no dissolution throughout the heating process). The mixture was filtered and the solid was collected and rinsed with 1:4 EtOH/H_2O. The solid was allowed to dry and then was transferred to a vial and azeotroped with MeCN (3x) to afford peptides **8-10** as white solids.

Characterization of compound **8**

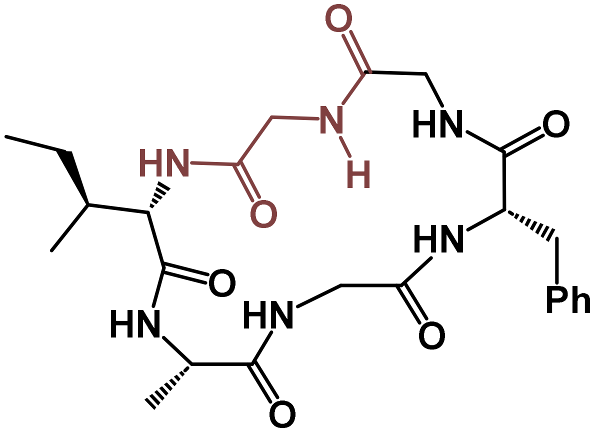

15.6 mg of a white solid obtained in a 30% overall yield (based on 100 % resin loading). 1H NMR (500 MHz, dmso) δ 8.49 (t, *J* = 5.9 Hz, 1H), 8.44 (d, *J* = 6.7 Hz, 1H), 8.09 (d, *J* = 7.9 Hz, 1H), 8.04 (d, *J* = 6.4 Hz, 1H), 7.65 – 7.60 (m, 1H), 7.58 – 7.53 (m, 1H), 7.27 (t, *J* = 7.5 Hz, 2H), 7.19 (t, *J* = 8.2 Hz, 3H), 4.26 (t, *J* = 7.2 Hz, 2H), 3.98 – 3.93 (m, 2H), 3.93 – 3.83 (m, 2H), 3.79 – 3.72 (m, 1H), 3.51 – 3.37 (m, 2H), 3.06 (dd, *J* = 13.8, 5.9 Hz, 1H), 2.84 (dd, *J* = 13.7, 9.0 Hz, 1H), 1.43 (dt, *J* = 12.3, 5.9 Hz, 1H), 1.28 – 1.17 (m, 4H), 0.90 – 0.81 (m, 6H). HRMS (ESI+) m/z calculated for C24 H35 N6 O6+ $[M+H]^+$ = 503.2623, found = 503.2613.

Figure S17:1H-NMR **8** (500 MHz, DMSO-*d*6)

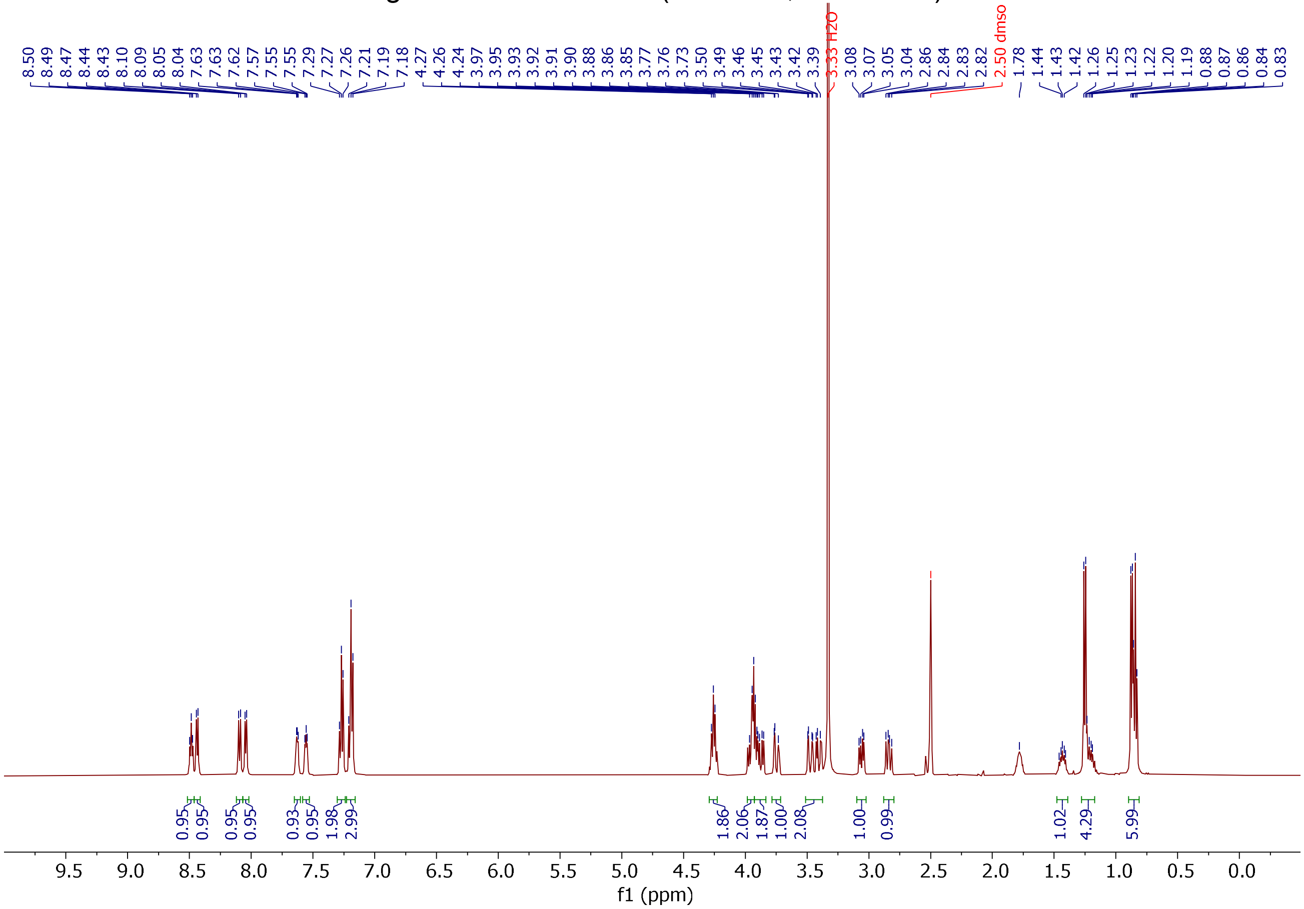

Figure S18: 2D-COSY Compound **8** (500 MHz, DMSO-*d*6)

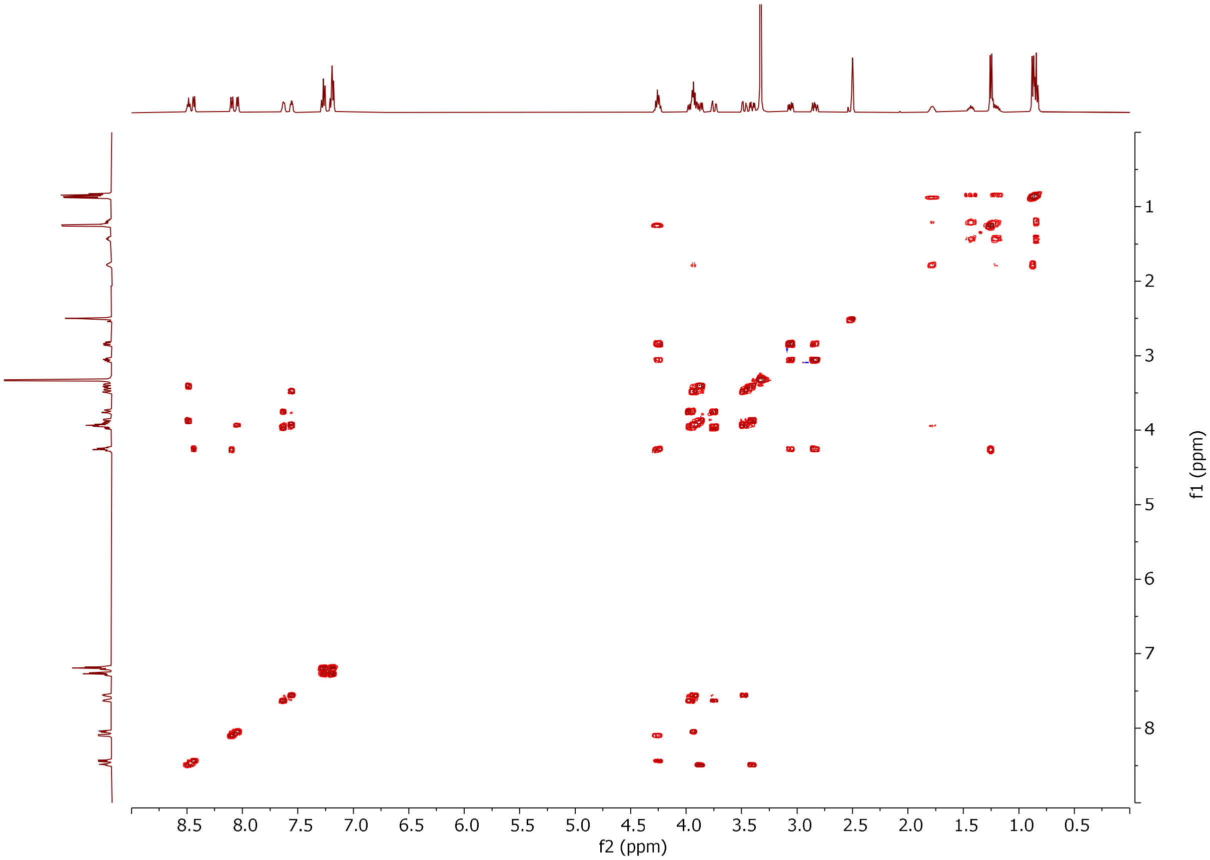

Figure S19: 2D-TOCSY Compound **8** (500 MHz, DMSO-*d*6)

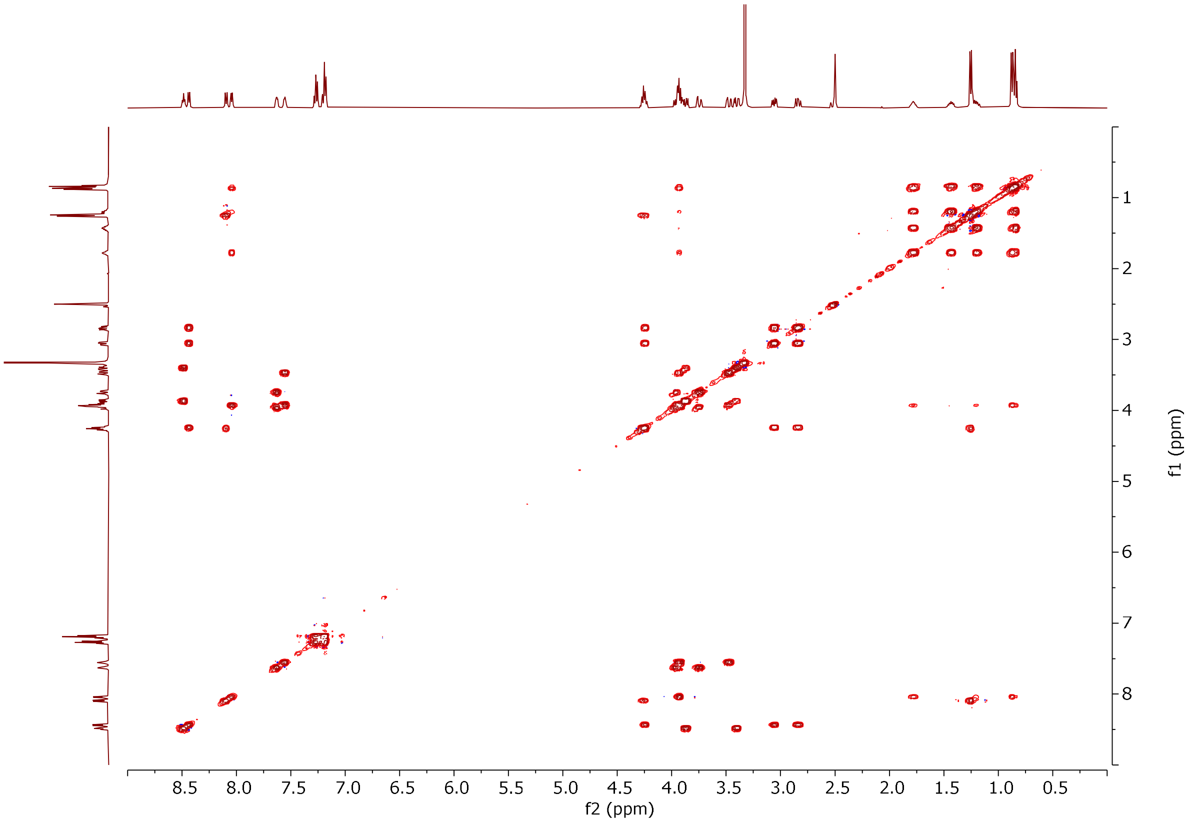

Figure S20: 2D-ROESY Compound **8** (500 MHz, DMSO-*d*6)

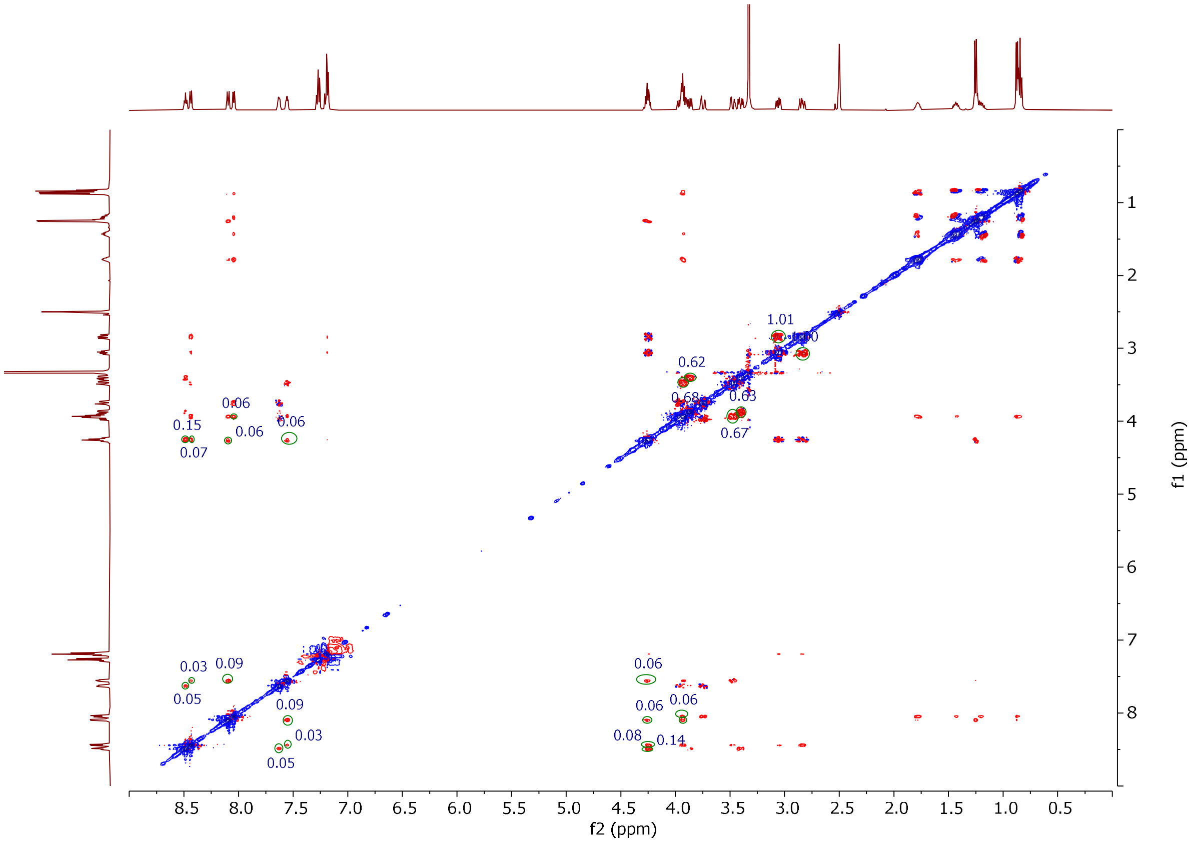

Figure S21: 2D-HSQC Compound **8** (500 MHz, DMSO-*d*6)

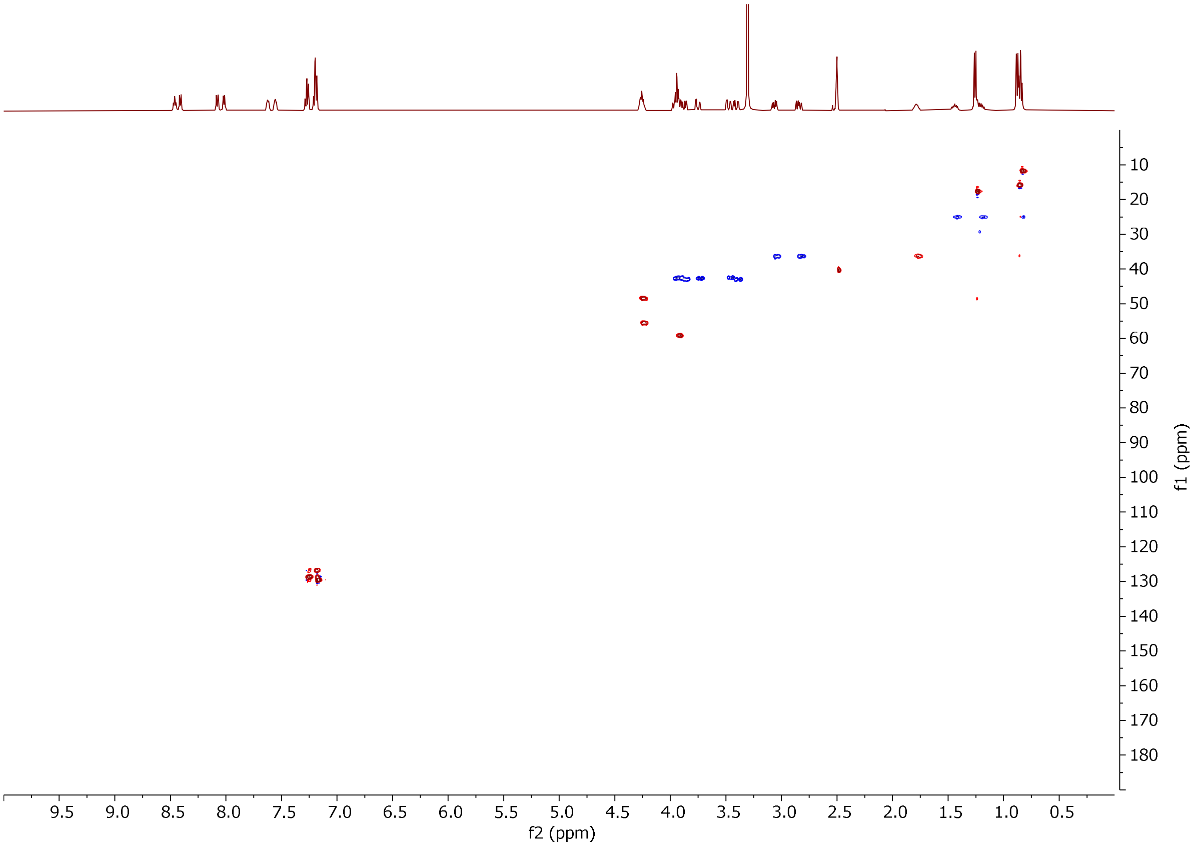

Figure S22: 2D-HMBC Compound **8** (500 MHz, DMSO-*d*6)

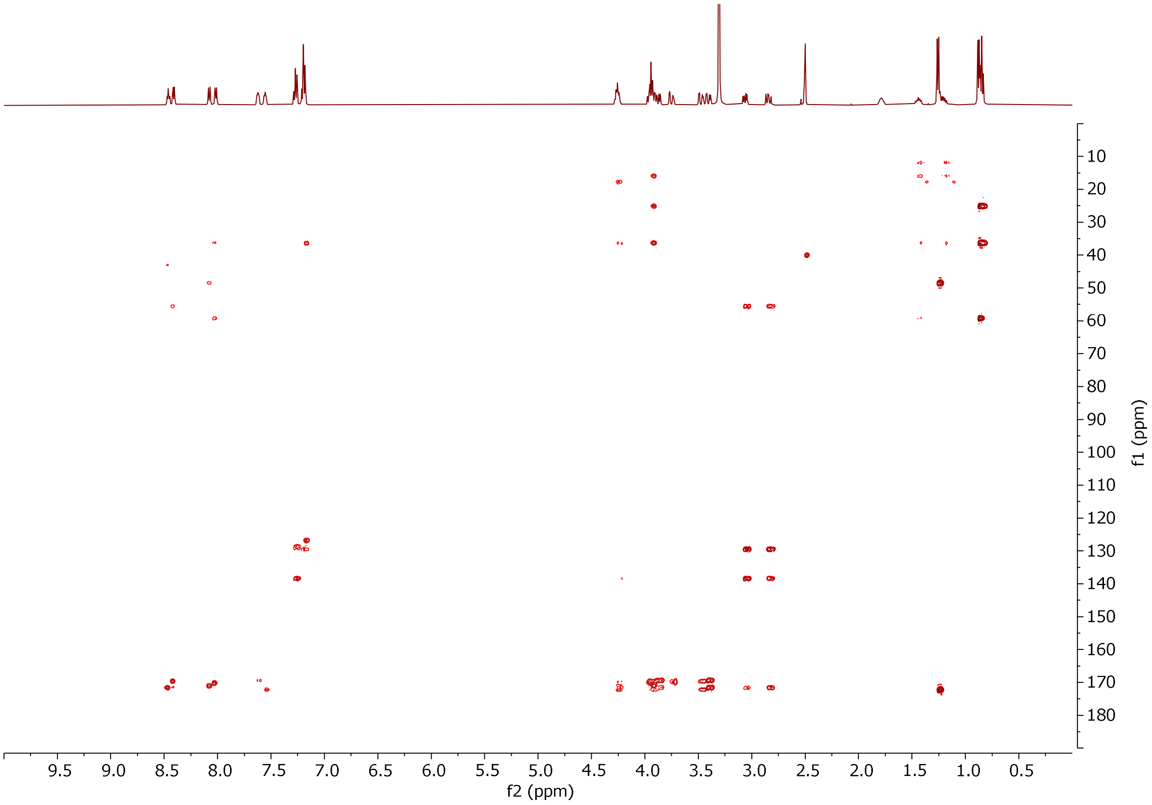

Figure S23: VT 1H-NMR Compound **8** (500 MHz, DMSO-*d*6)

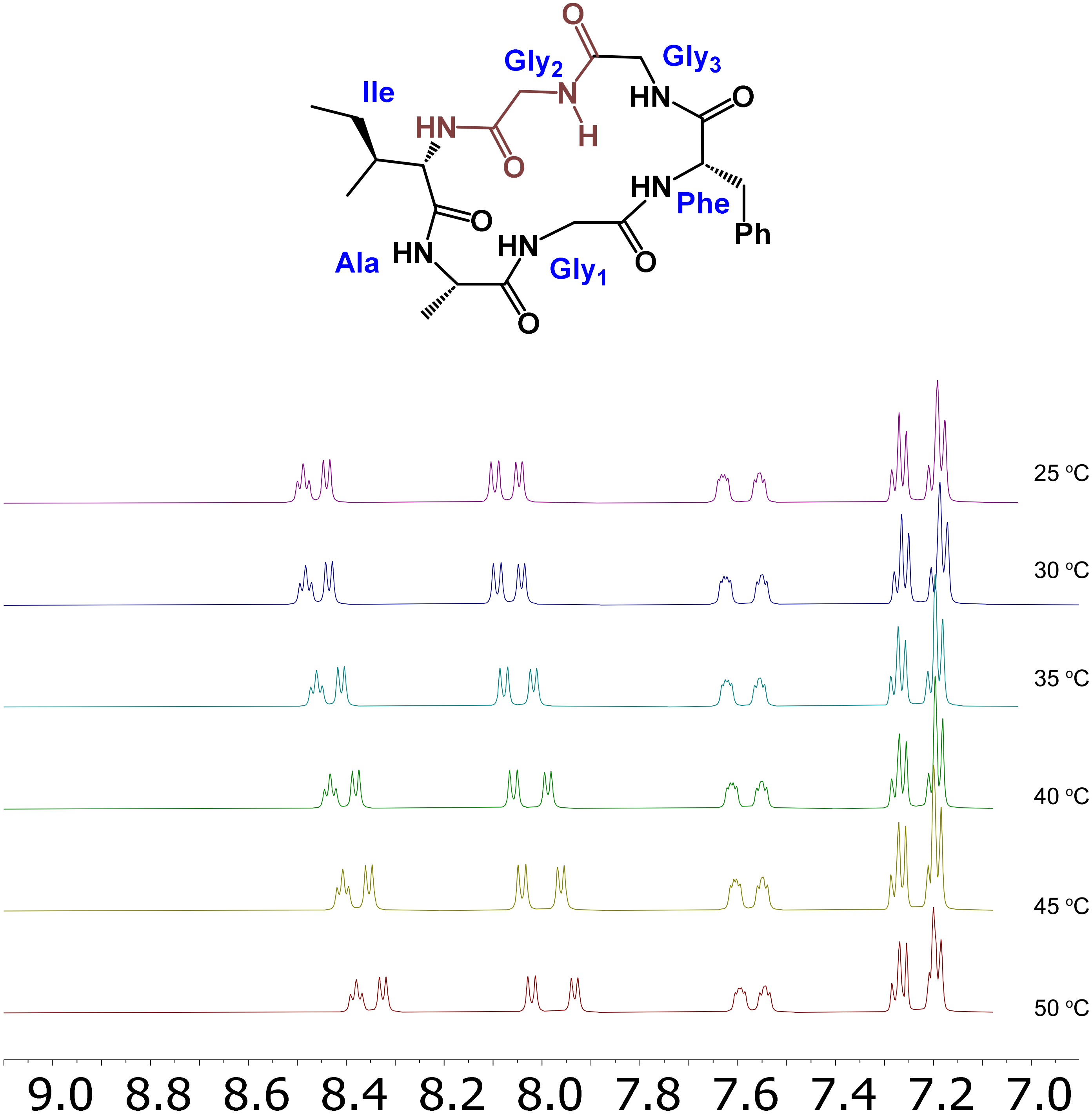

	Shifts (ppm)					
K	*AlaNH*	*IleNH*	*Gly2NH*	*Gly3NH*	*PheNH*	*Gly1NH*
**298**	8.11	8.04	7.63	8.48	8.44	7.56
**323**	8.02	7.93	7.59	8.38	8.32	7.54
Δppb/k						
**298-323**	**4.2**	**5.5**	**2**	**5**	**6**	**1**

Characterization of compound **9**

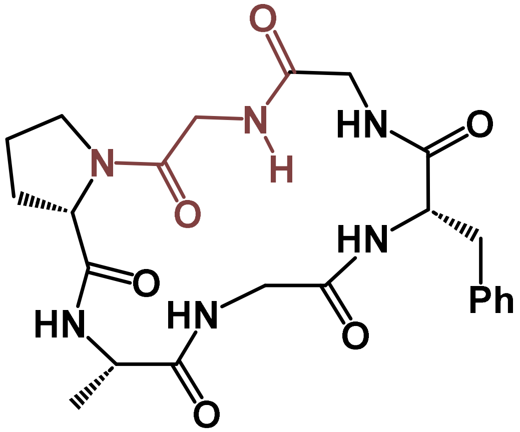

18.5 mg of a white solid obtained in a 38% overall yield (based on 100 % resin loading). ^{1}H NMR (500 MHz, dmso) δ 8.70 (dd, *J* = 7.2, 5.2 Hz, 1H), 8.65 (d, *J* = 6.4 Hz, 1H), 8.11 (d, *J* = 9.0 Hz, 1H), 7.48 (d, *J* = 5.8 Hz, 1H), 7.31 – 7.14 (m, 6H), 4.39 (p, *J* = 7.3 Hz, 1H), 4.29 (dd, *J* = 17.6, 7.5 Hz, 1H), 4.14 – 3.93 (m, 4H), 3.82 – 3.75 (m, 1H), 3.70 – 3.62 (m, 1H), 3.55 – 3.45 (m, 2H), 3.36 (d, *J* = 5.0 Hz, 1H), 3.09 (dd, *J* = 13.8, 5.5 Hz, 1H), 2.90 (dd, *J* = 13.8, 9.6 Hz, 1H), 2.24 – 2.12 (m, 1H), 1.87 (p, *J* = 6.8 Hz, 2H), 1.73 (h, *J* = 6.7 Hz, 1H), 1.25 (d, *J* = 7.3 Hz, 3H). HRMS (ESI+) m/z calculated for C23 H31 N6 O6+ $[M+H]^{+}$ = 487.2299, found = 487.2300.

Figure S24:1H-NMR **9** (500 MHz, DMSO-*d*6)

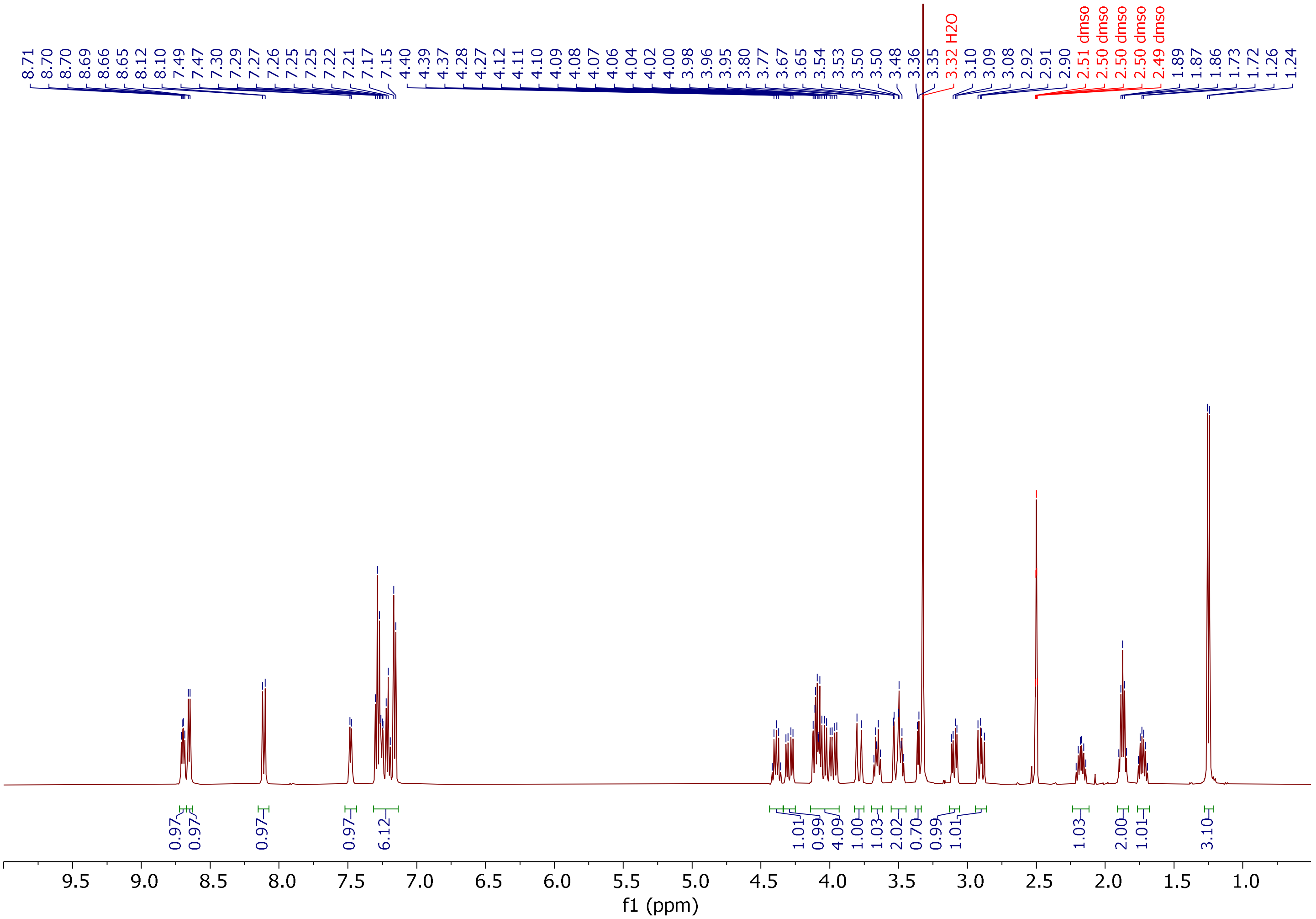

Figure S25: 2D-COSY Compound **9** (500 MHz, DMSO-*d*6)

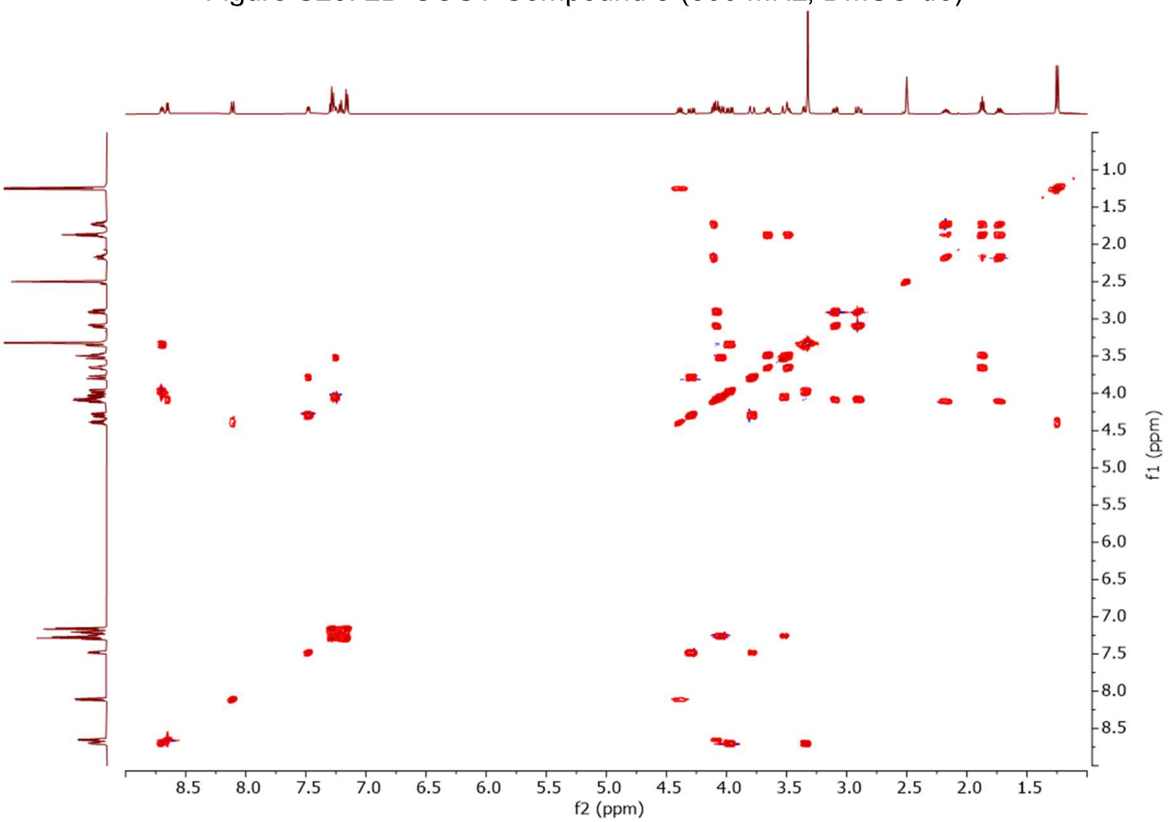

Figure S26: 2D-TOCSY Compound **9** (500 MHz, DMSO-*d*6)

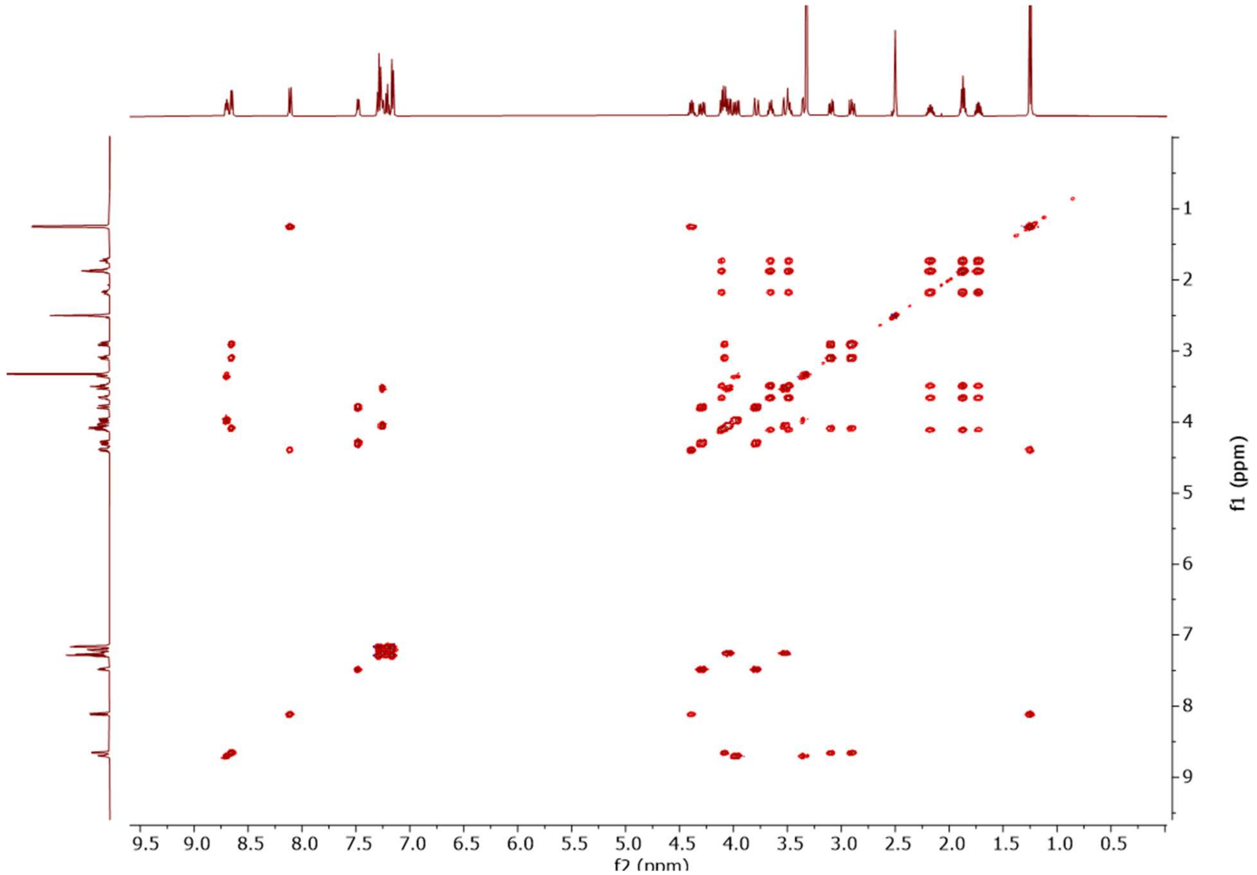

Figure S27: 2D-ROESY Compound **9** (500 MHz, DMSO-*d*6)

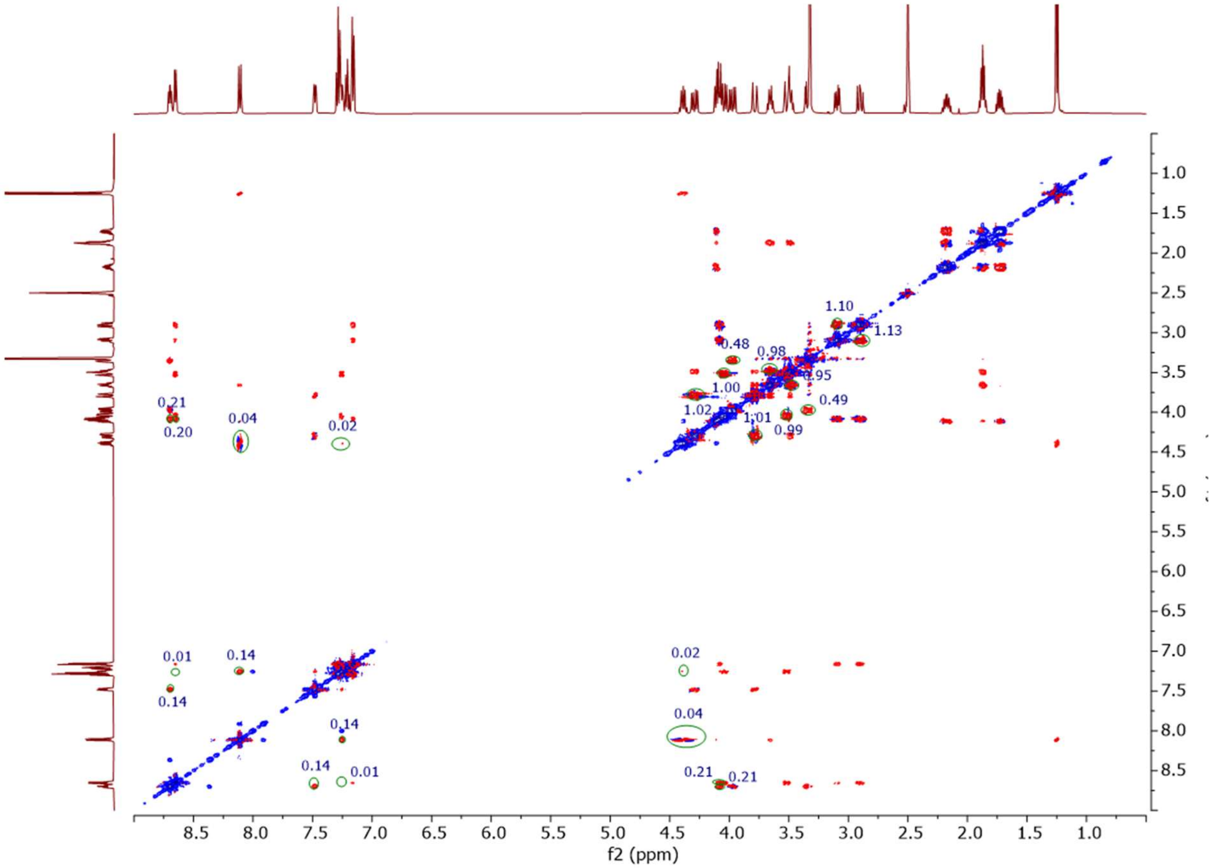

Figure S28: 2D-HSQC Compound **9** (500 MHz, DMSO-*d*6)

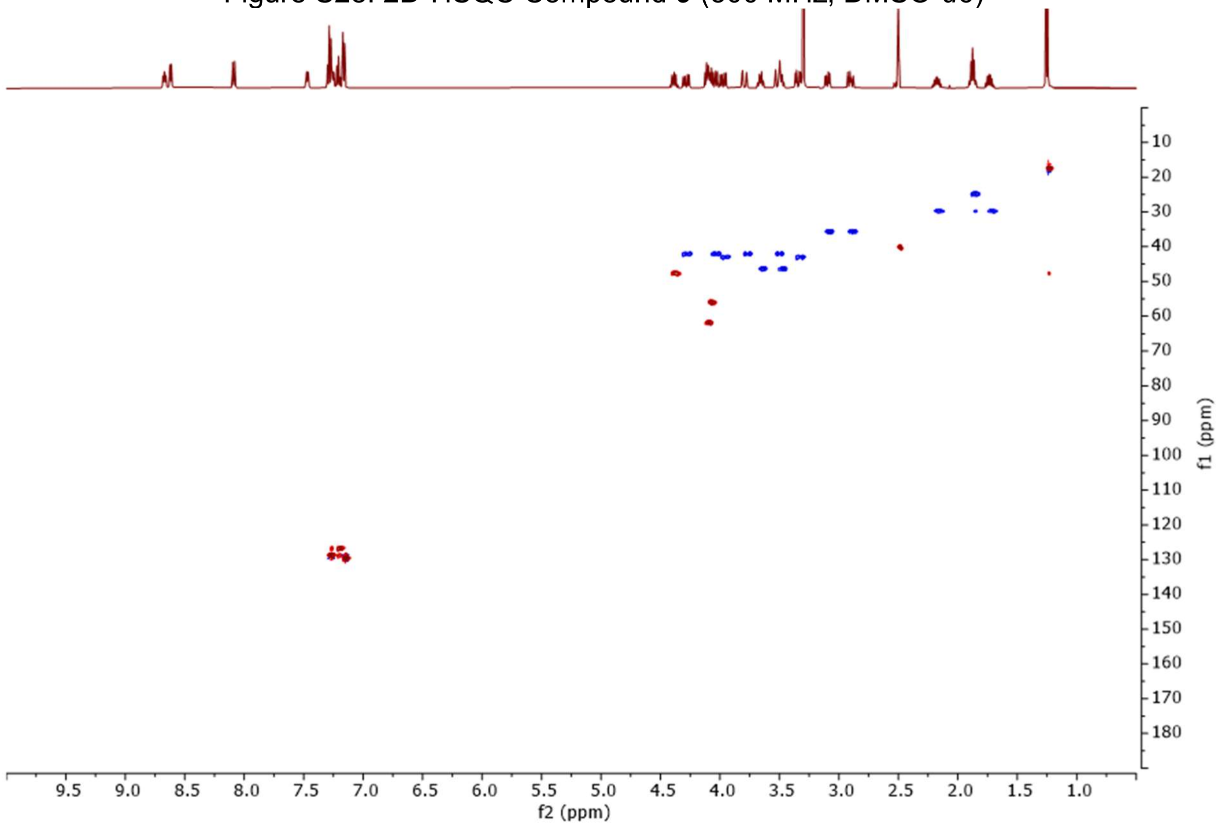

Figure S29: 2D-HMBC Compound **9** (500 MHz, DMSO-*d*6)

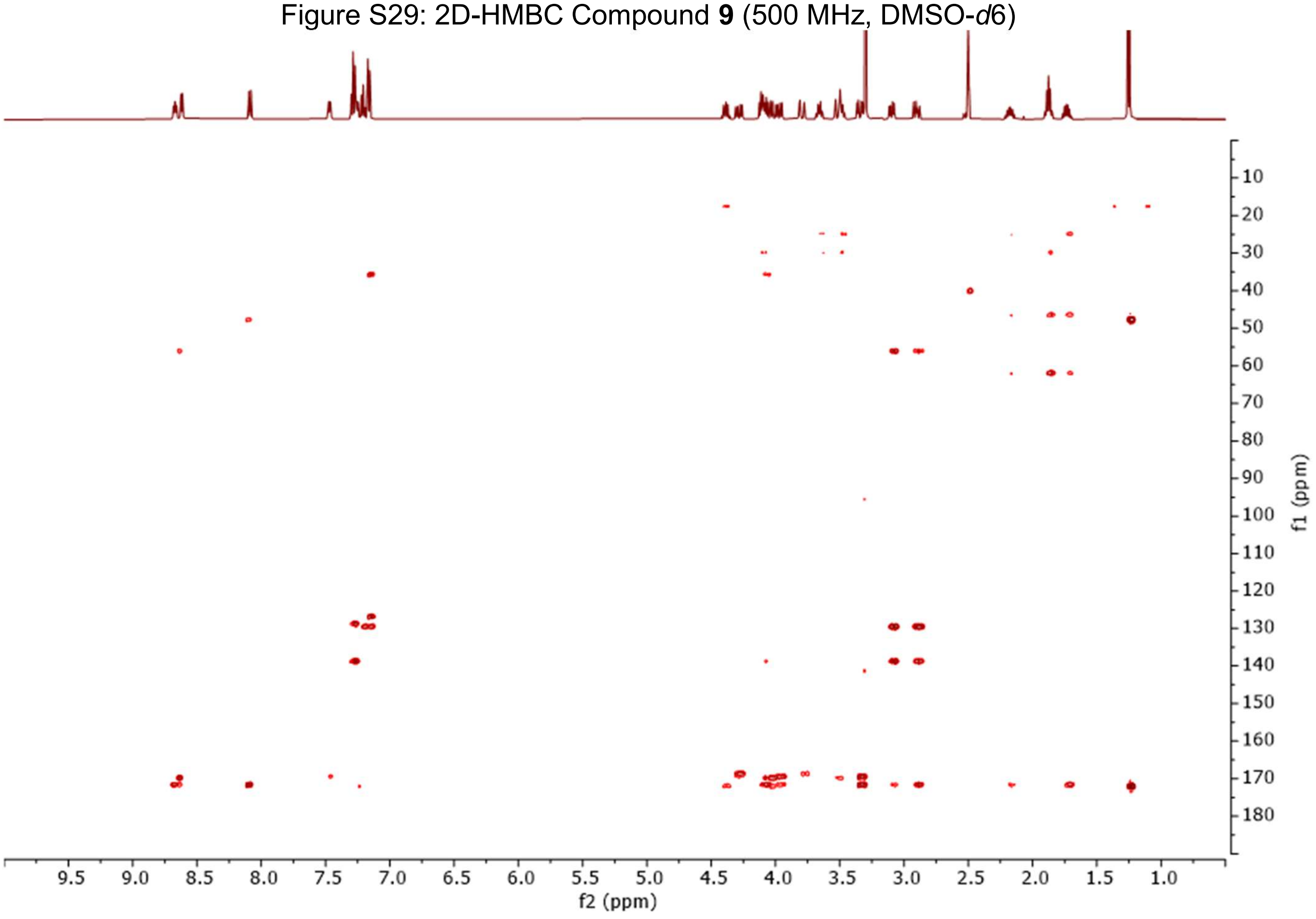

Figure S30: VT 1H-NMR Compound **9** (500 MHz, DMSO-*d*6)

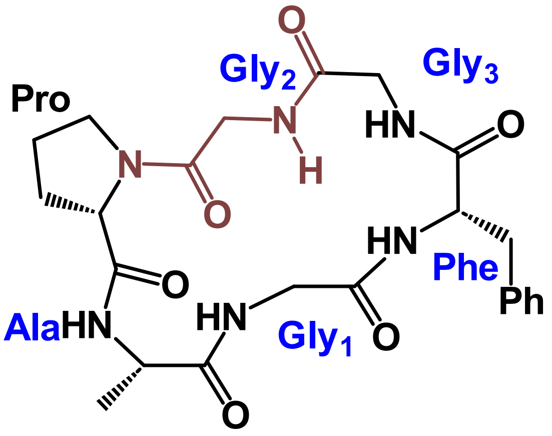

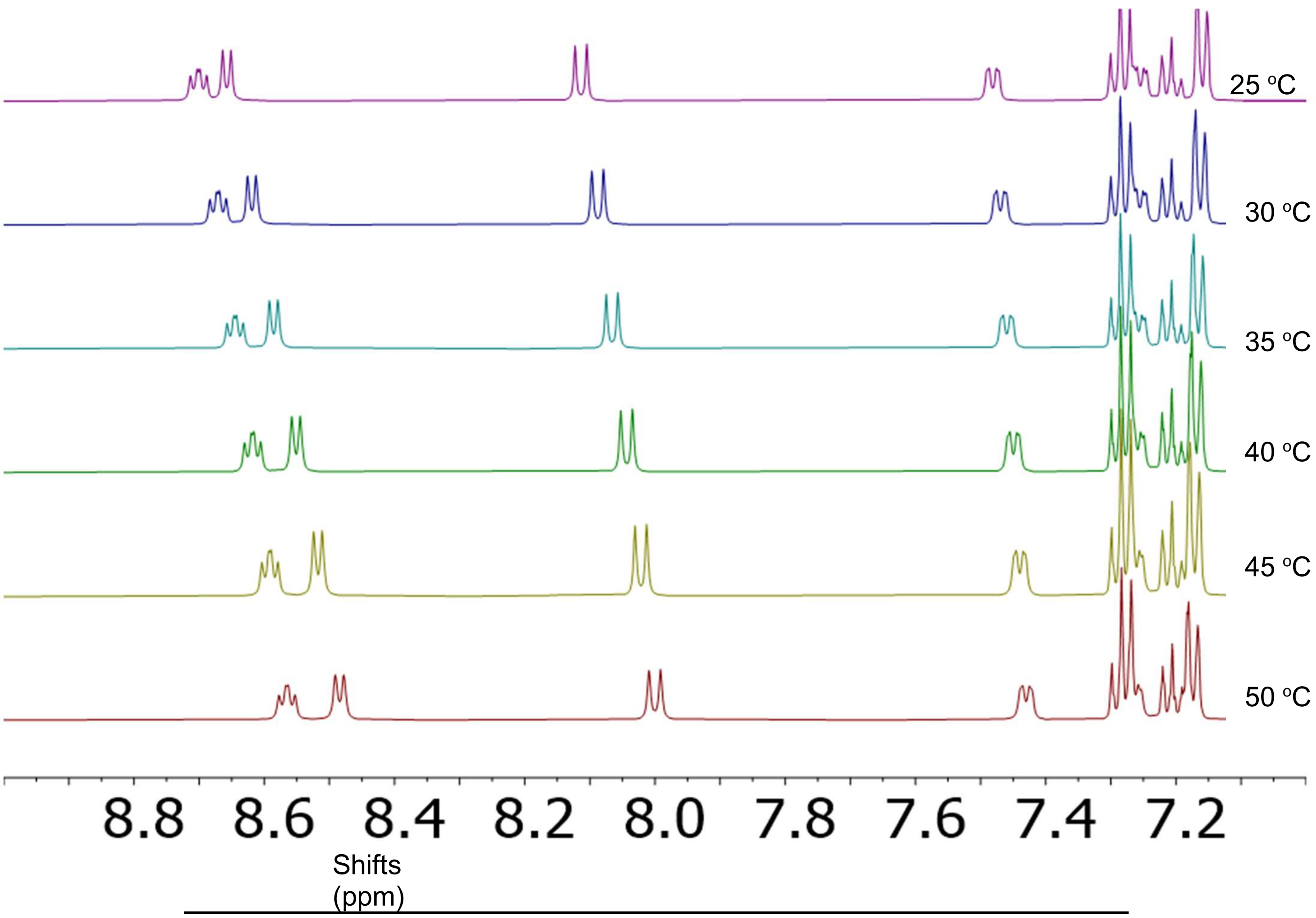

K	AlaNH	Pro	Gly2NH	Gly3NH	PheNH	Gly1NH
**298**	8.11		7.48	8..7	8.65	7.25
**323**	8.01		7.42	8.56	8.48	7.26
Δppb/k						
**298-323**	5.5		**3**	**7**	**8.5**	**-0.5**

Characterization of compound **10**

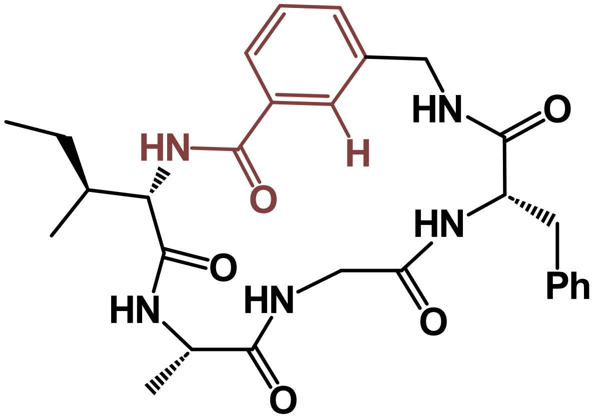

16.7 mg of a white solid obtained in a 32% overall yield (based on 100 % resin loading). 1H NMR (500 MHz, dmso) δ 8.52 (t, *J* = 6.2 Hz, 1H), 8.36 (d, *J* = 7.3 Hz, 1H), 8.24 (d, *J* = 8.0 Hz, 1H), 8.05 (d, *J* = 8.3 Hz, 1H), 7.75 (d, *J* = 5.7 Hz, 1H), 7.69 (s, 1H), 7.58 (d, *J* = 6.8 Hz, 1H), 7.39 – 7.36 (m, 2H), 7.30 – 7.18 (m, 6H), 4.57 – 4.49 (m, 2H), 4.35 – 4.29 (m, 1H), 4.09 – 3.99 (m, 2H), 3.89 (dd, *J* = 16.8, 6.0 Hz, 1H), 3.42 (dd, *J* = 16.4, 4.6 Hz, 1H), 3.00 (dd, *J* = 14.0, 5.9 Hz, 1H), 2.80 – 2.71 (m, 1H), 1.91 (s, 1H), 1.54 – 1.50 (m, 1H), 1.20 - 1.29 (m), 0.94 – 0.81 (m, 7H). HRMS (ESI+) m/z calculated for C28 H36 N5 O5+ [M+H]$^+$ = 522.2719, found = 522.2711.

Figure S31:1H-NMR **10** (500 MHz, DMSO-*d*6)

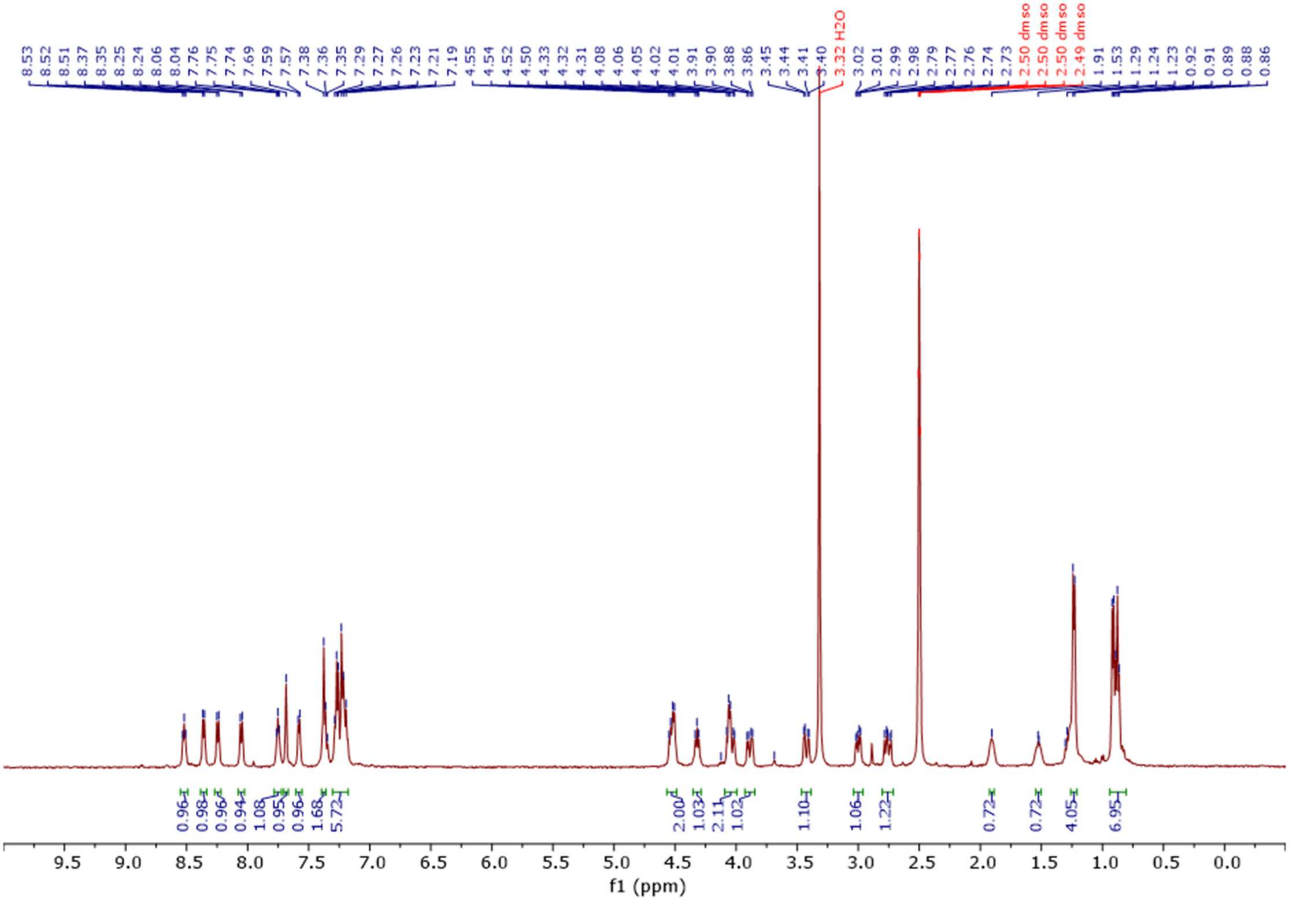

Figure S32: 2D-COSY Compound **10** (500 MHz, DMSO-*d*6)

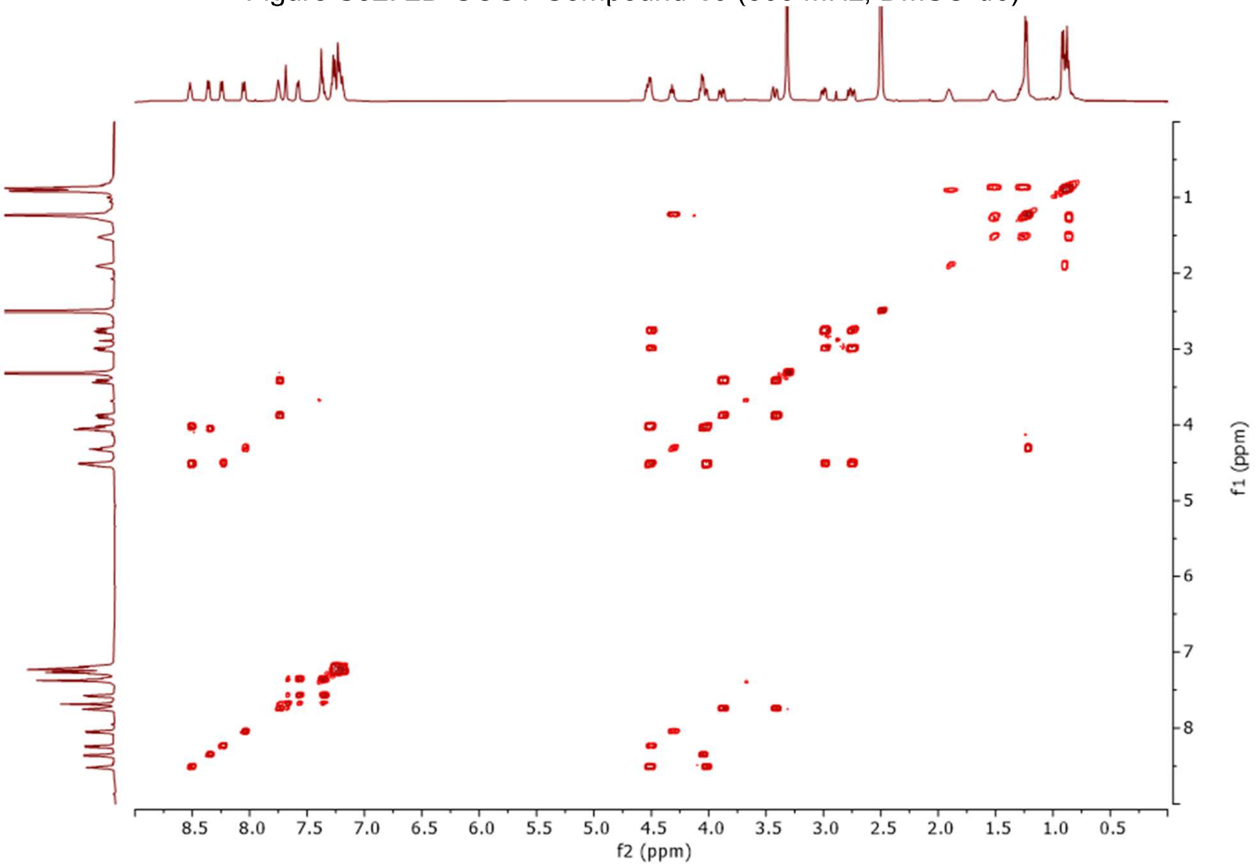

Figure S33: 2D-TOCSY Compound **10** (500 MHz, DMSO-*d*6)

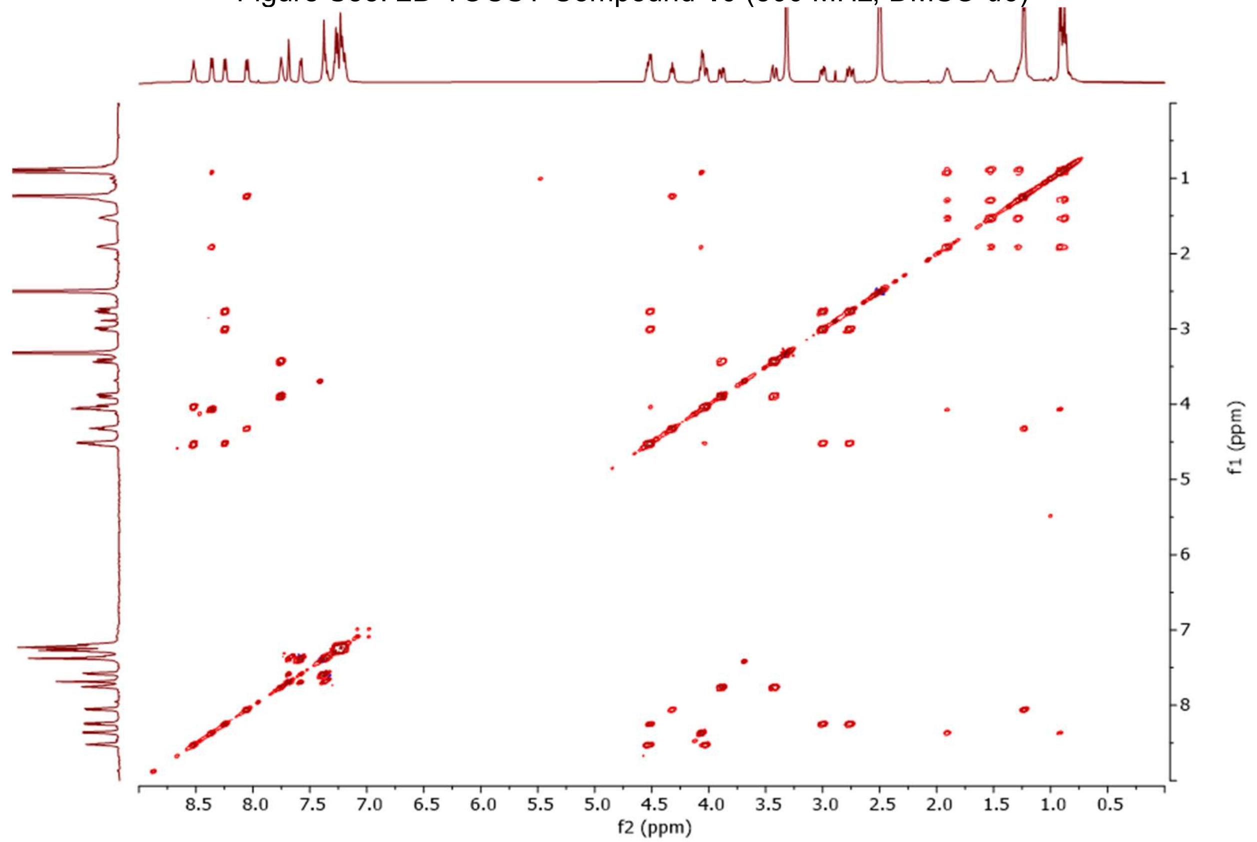

Figure S34: 2D-ROESY Compound **10** (500 MHz, DMSO-*d*6)

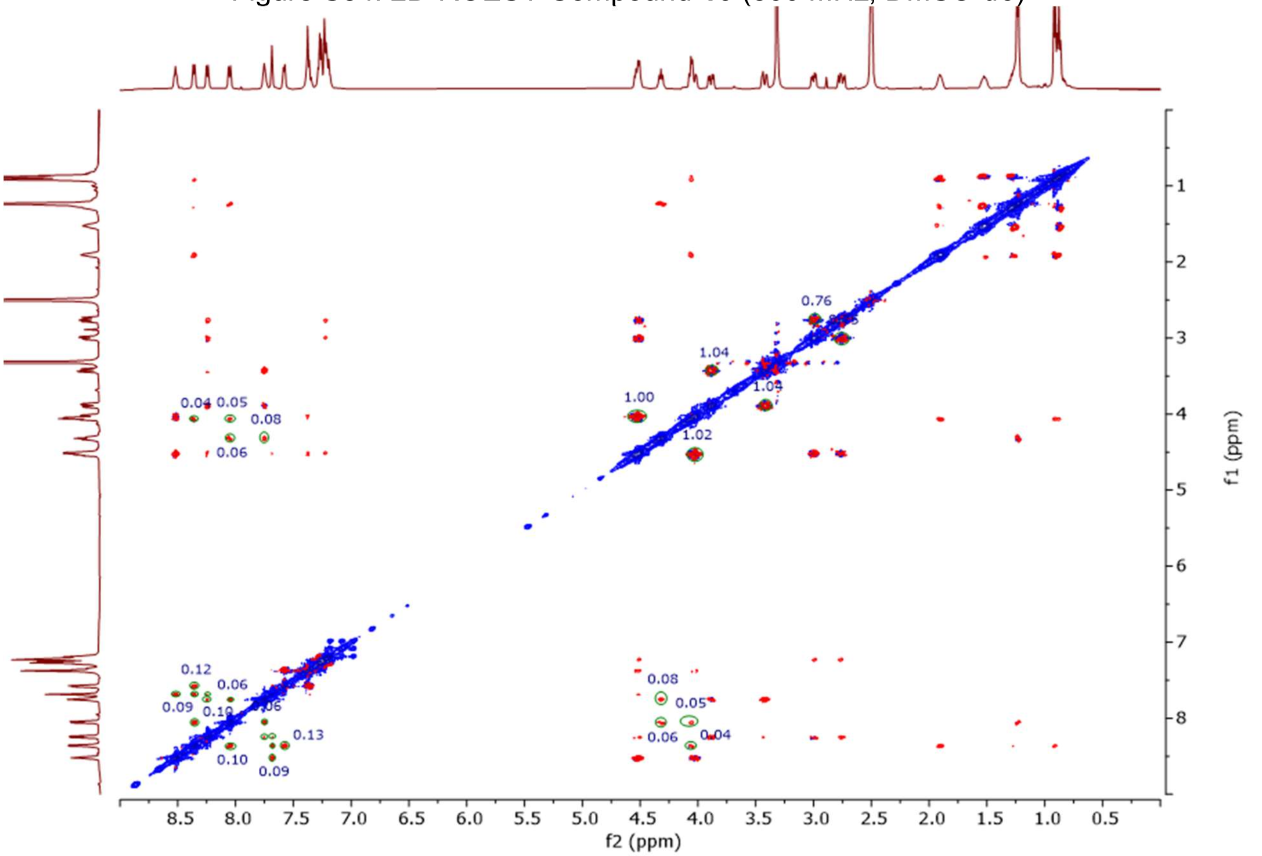

Figure S35: 2D-HSQC Compound **10** (500 MHz, DMSO-*d*6)

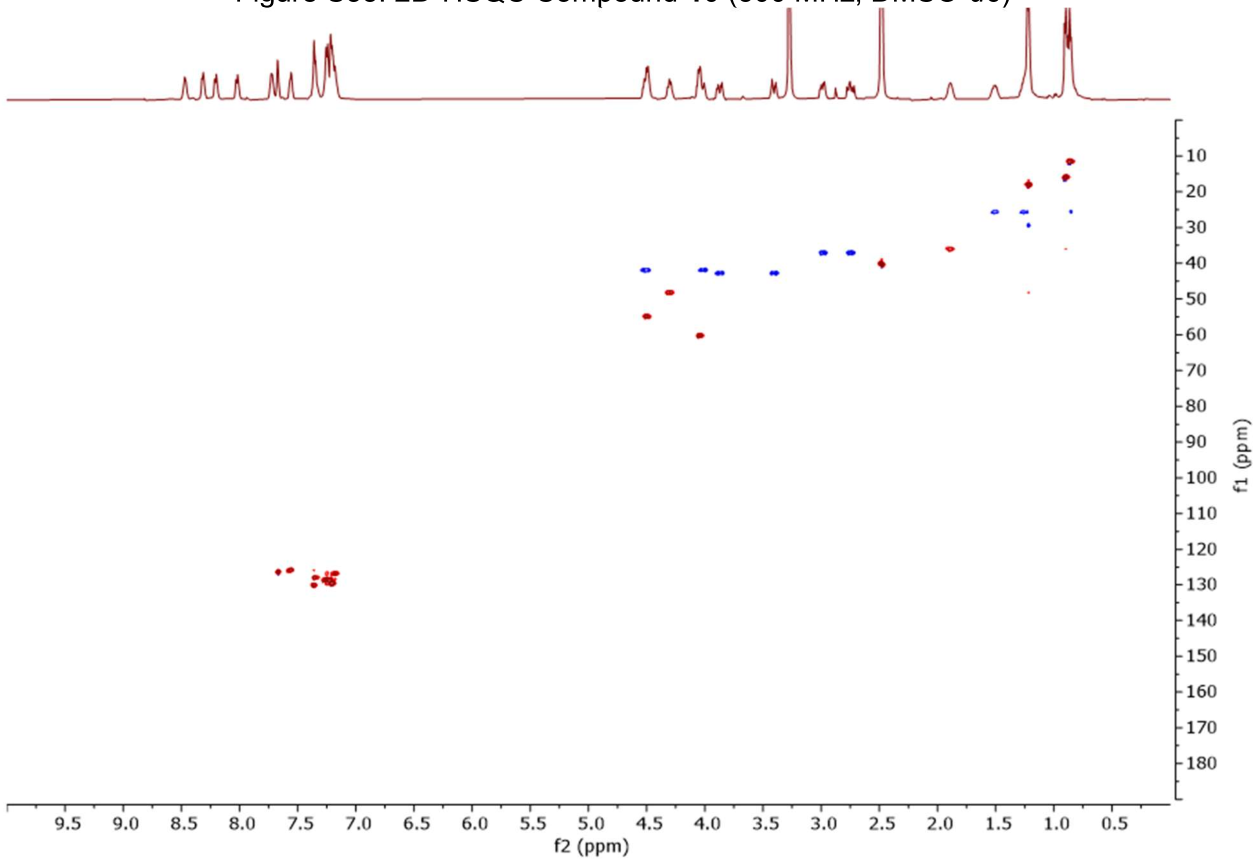

Figure S36: 2D-HMBC Compound **10** (500 MHz, DMSO-*d*6)

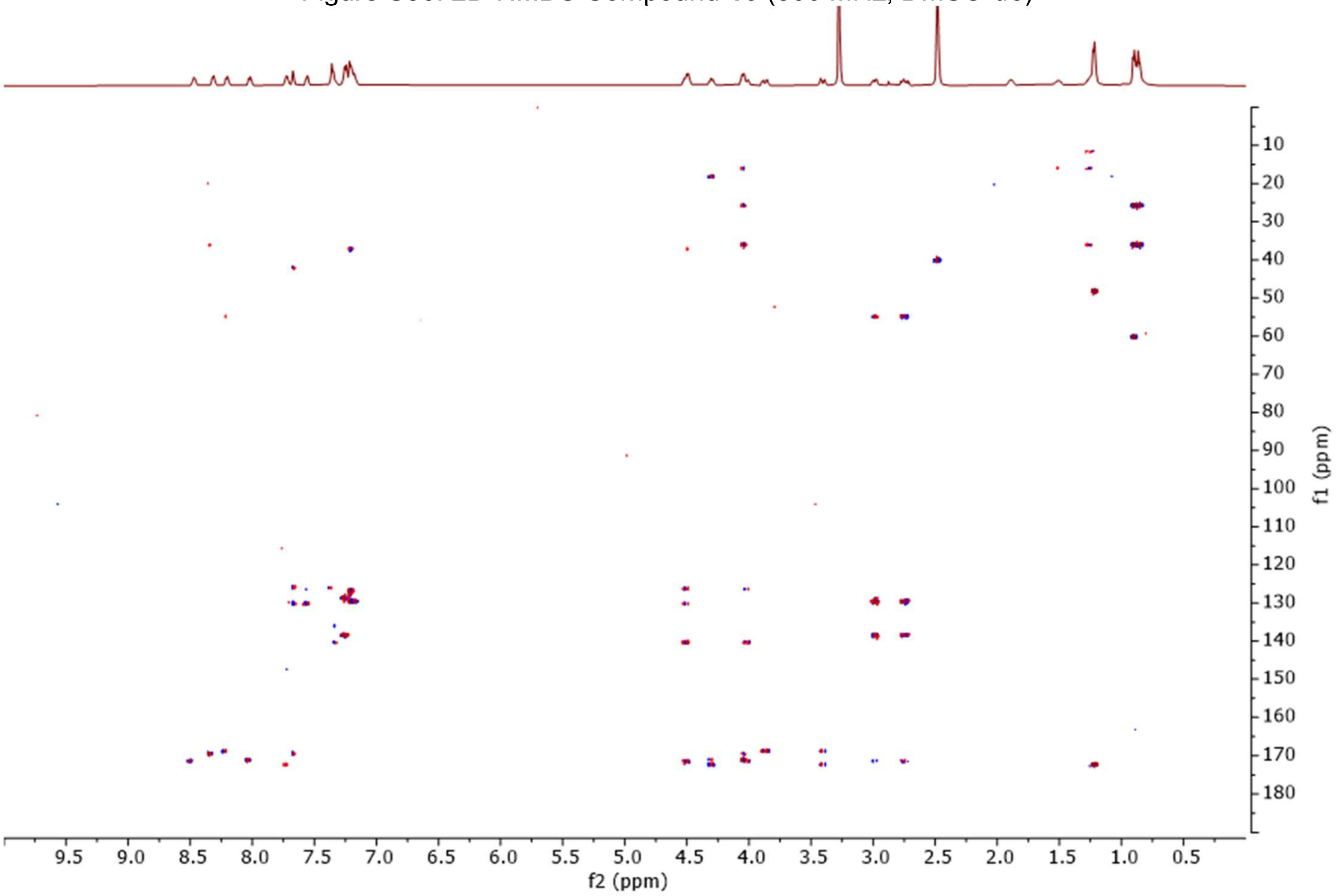

Figure S37: VT 1H-NMR Compound **10** (500 MHz, DMSO-*d*6)

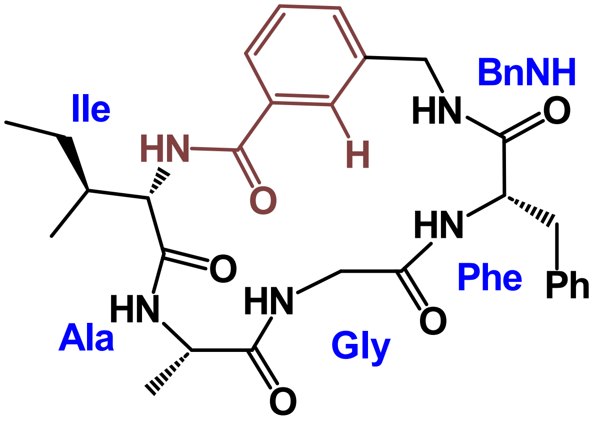

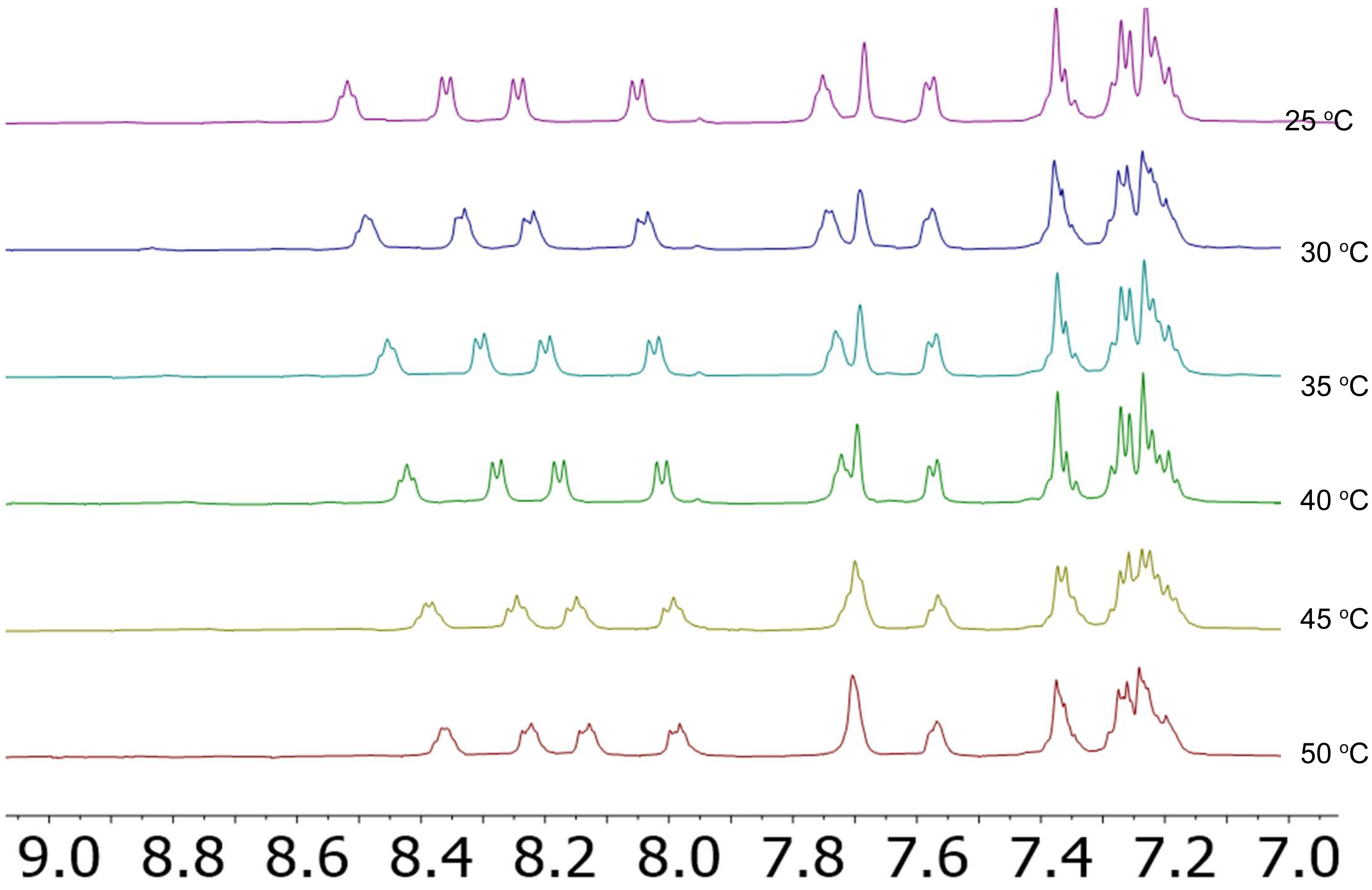

	Shifts (ppm)				
K	*AlaNH*	*Ile*	*BnNH*	*Phe*	*GlyNH*
**298**	8.05	8.36	8.52	8.24	7.75
**323**	7.98	8.23	8.36	8.13	7.69
Δppb/k					
**298-323**	2.8	5.2	6.4	4.4	2.4

Characterization of compound **11**

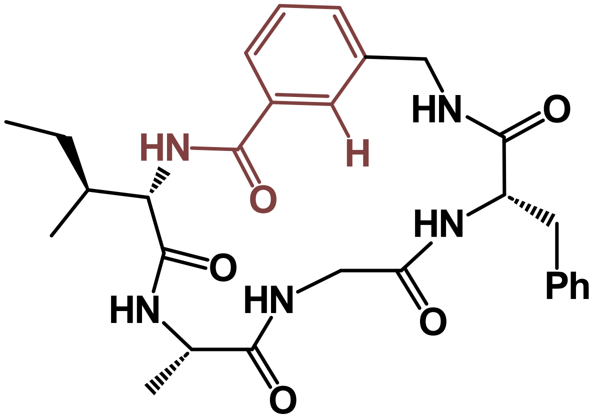

20.2 mg of a white solid obtained in a 40% overall yield (based on 100 % resin loading). ^{1}H NMR (500 MHz, dmso) δ 8.49 (s, 1H), 8.05 (d, *J* = 7.8 Hz, 1H), 7.96 (d, *J* = 8.0 Hz, 1H), 7.53 (s, 1H), 7.38 – 7.32 (m, 3H), 7.31 – 7.24 (m, 3H), 7.20 (dd, *J* = 13.7, 6.9 Hz, 5H), 4.72 (dd, *J* = 15.9, 7.7 Hz, 1H), 4.55 (s, 1H), 4.43 – 4.27 (m, 2H), 4.17 – 3.80 (m, 3H), 3.75 (s, 2H), 3.58 (s, 1H), 3.43 (d, *J* = 16.1 Hz, 1H), 3.34 (s, 1H), 3.05 (s, 1H), 2.79 (s, 1H), 2.23 (s, 1H), 1.99 – 1.82 (m, 4H), 1.28 (d, *J* = 6.9 Hz, 3H). HRMS (ESI+) m/z calculated for C27 H32 N5 O5+ $[M+H]^{+}$ = 506.2415, found = 506.2398.

Figure S38:1H-NMR **11** (500 MHz, DMSO-*d*6)

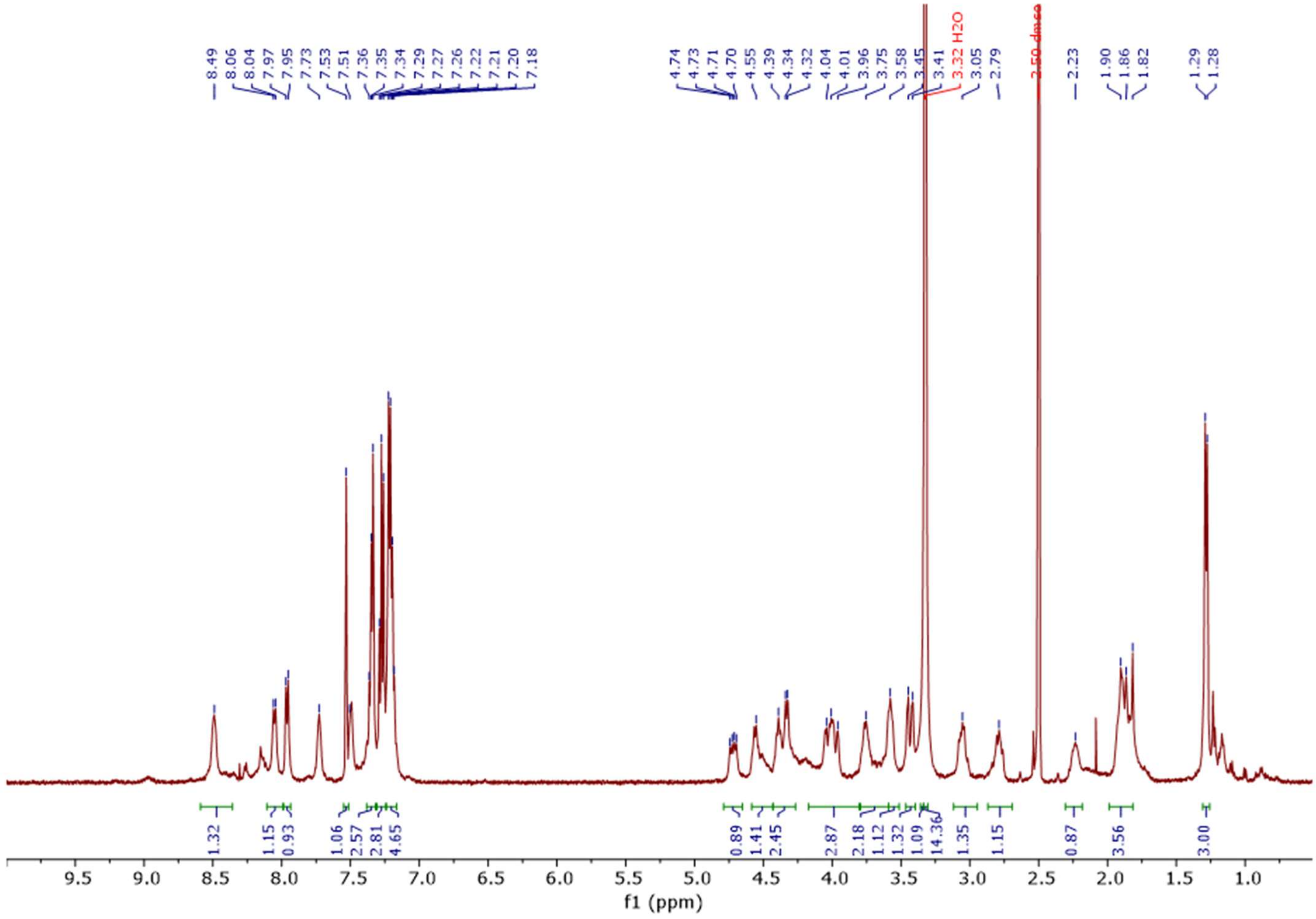

Figure S39: 2D-COSY Compound **11** (500 MHz, DMSO-*d*6)

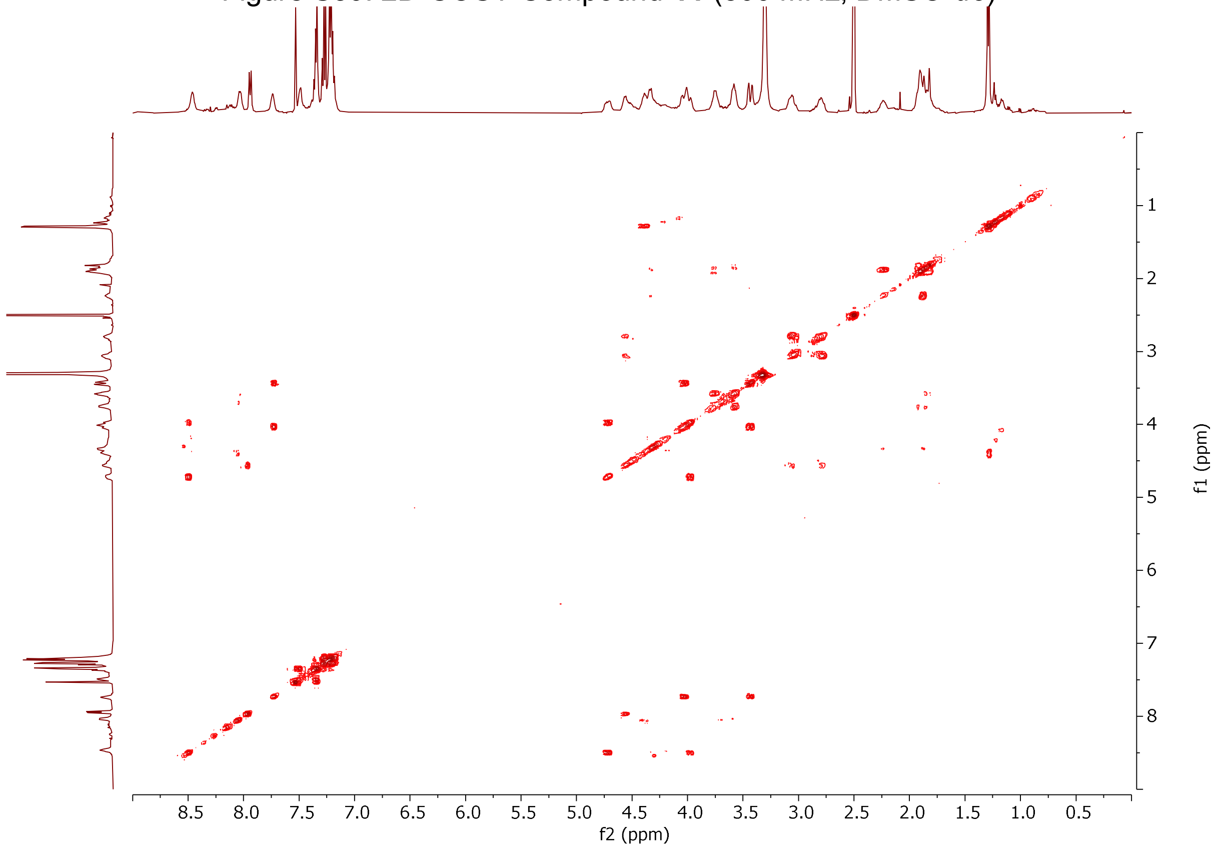

Figure S40: 2D-TOCSY Compound **11** (500 MHz, DMSO-*d*6)

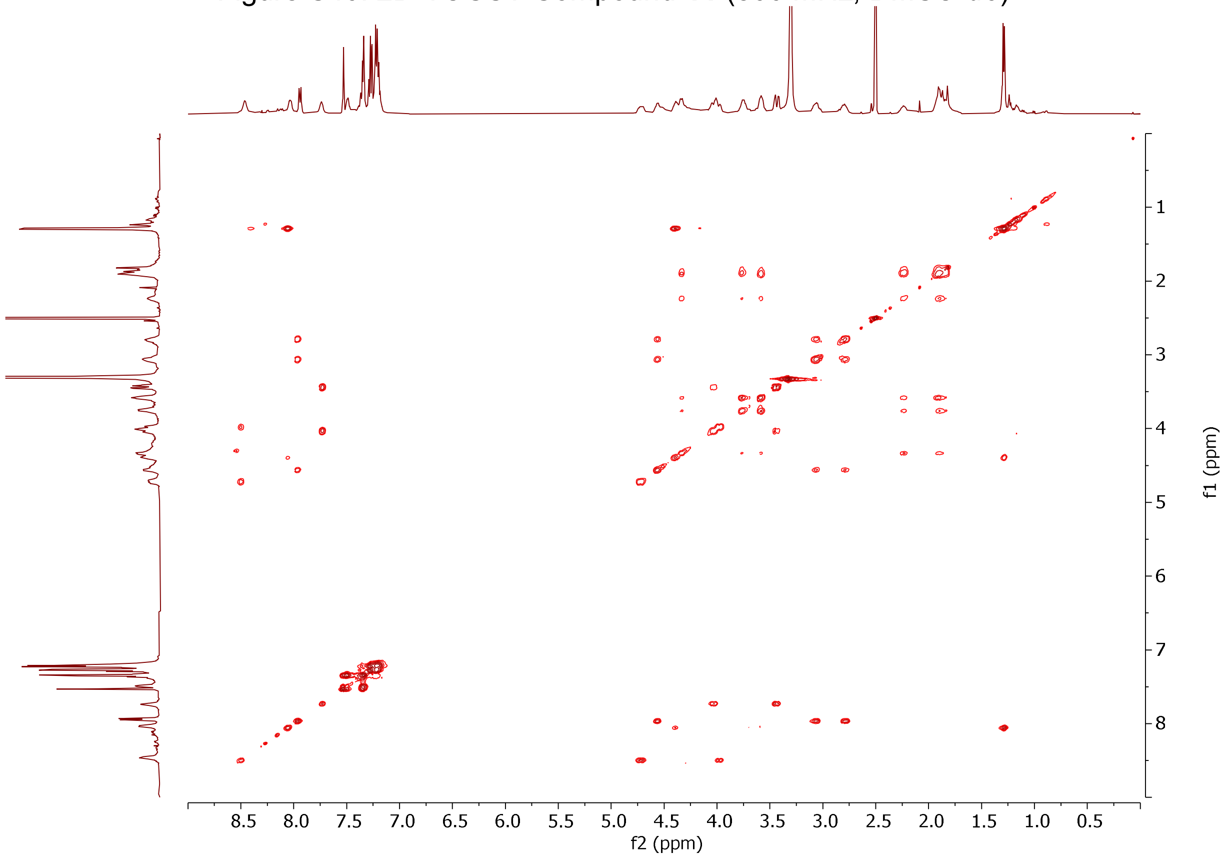

Figure S41: 2D-ROESY Compound **11** (500 MHz, DMSO-*d*6)

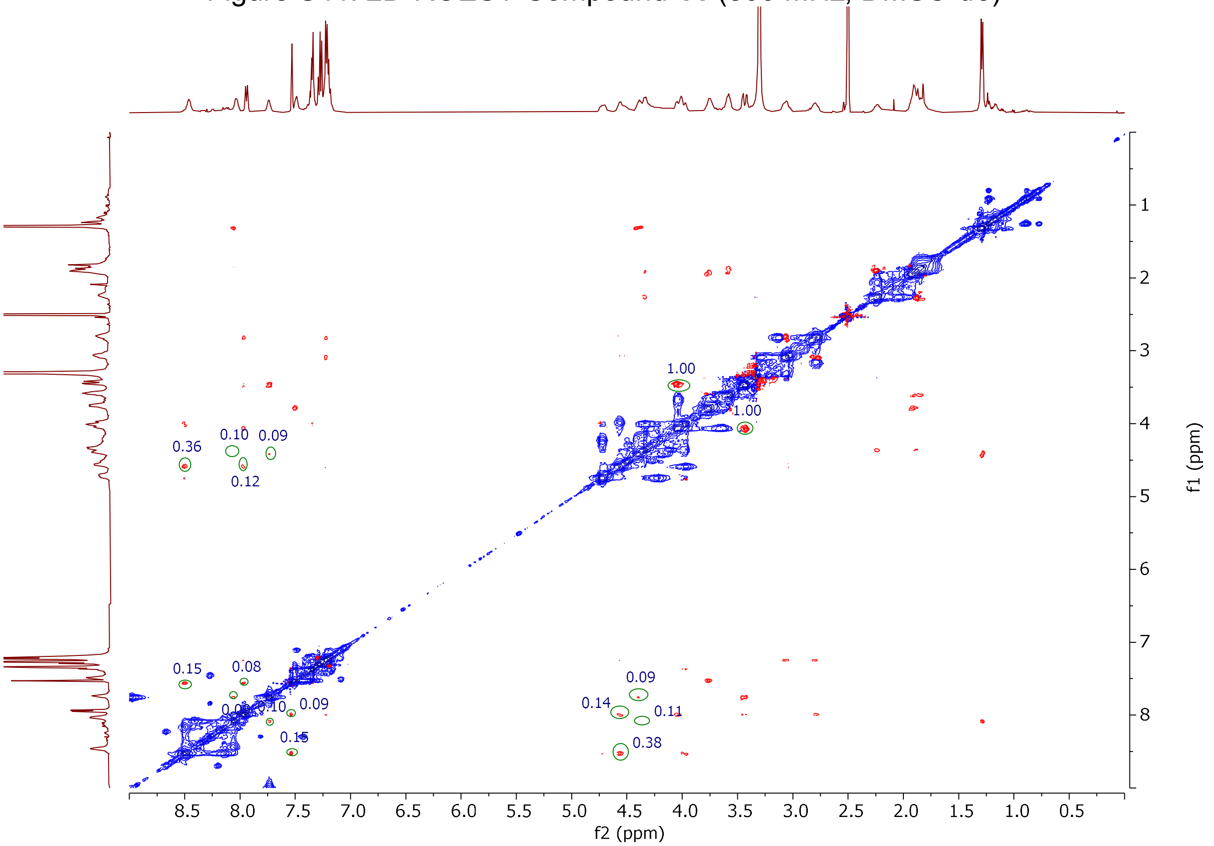

Figure S42: 2D-HSQC Compound **11** (500 MHz, DMSO-*d*6)

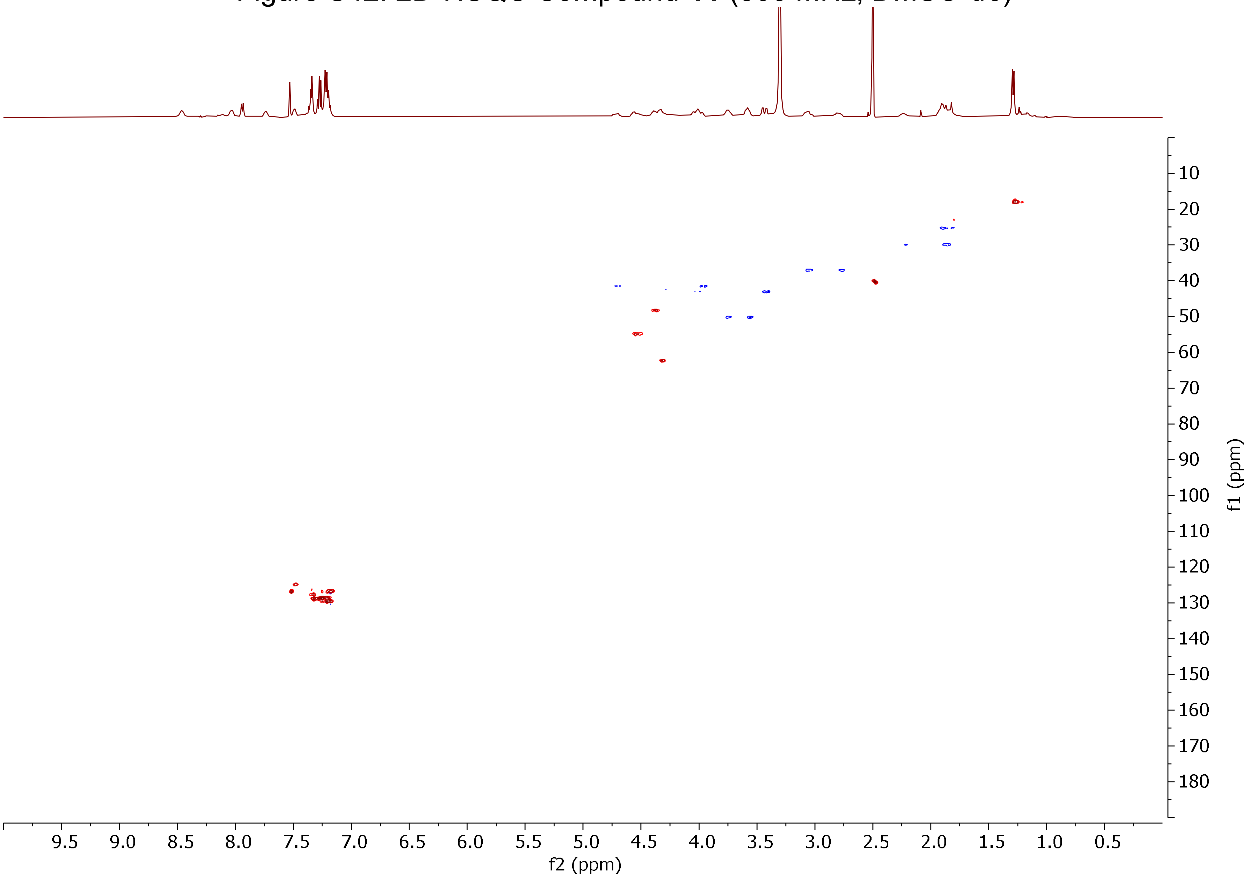

Figure S43: 2D-HMBC Compound **11** (500 MHz, DMSO-*d6*)

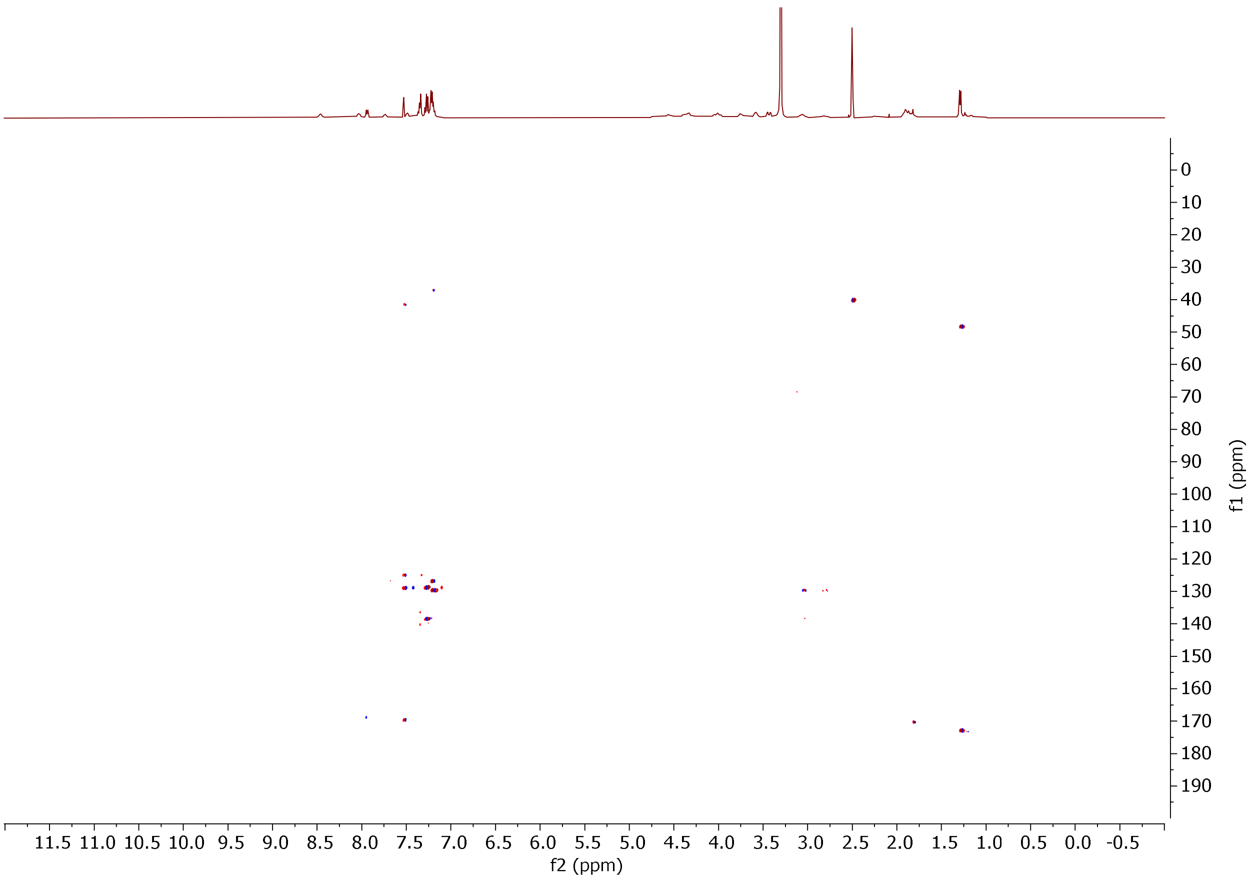

Figure S44: VT 1H-NMR Compound **11** (500 MHz, DMSO-*d6*)

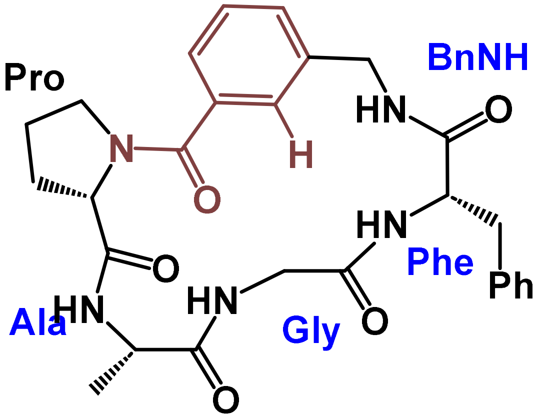

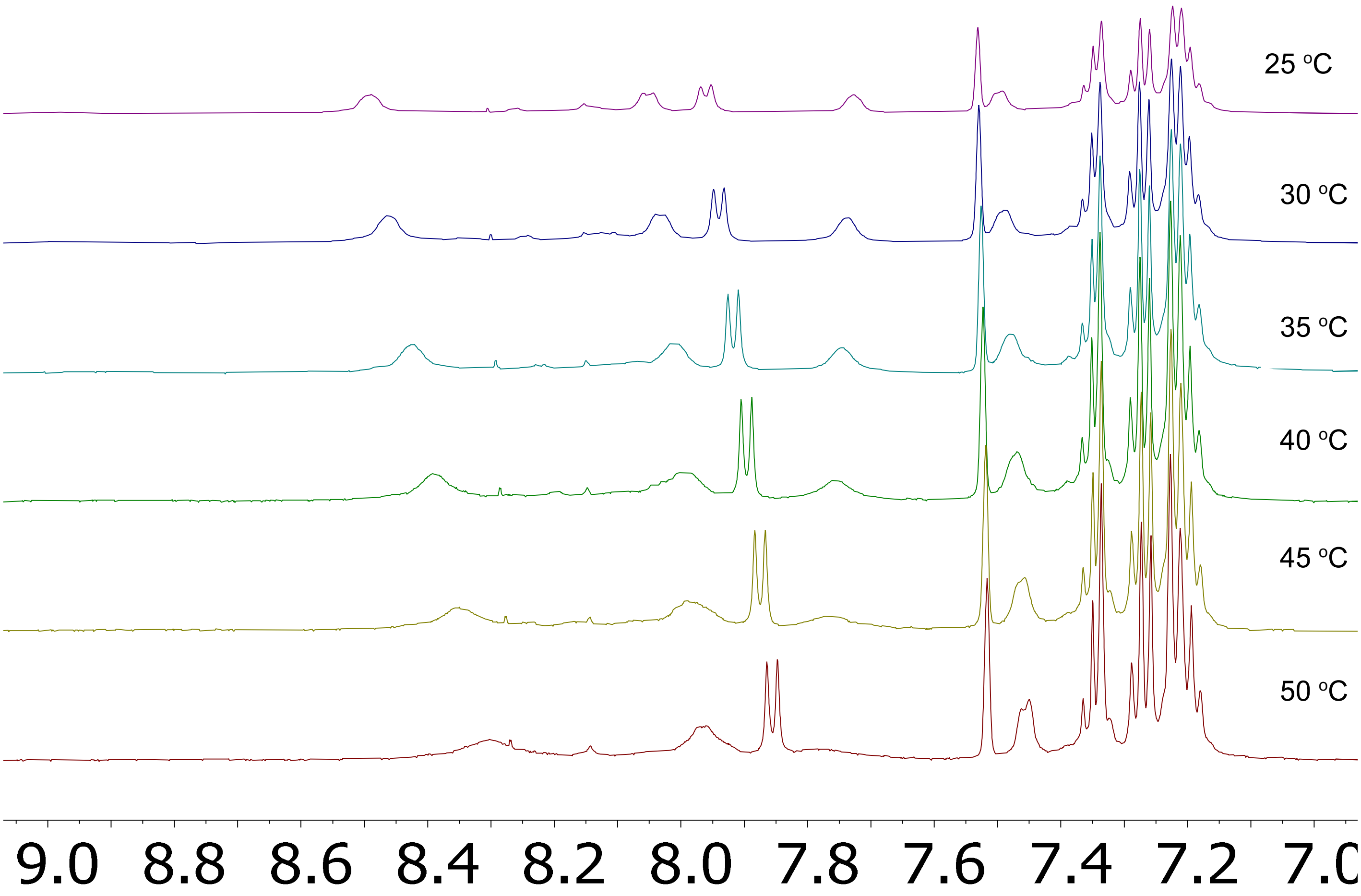

	Shifts (ppm)				
K	*AlaNH*	*Pro*	*BnNH*	*Phe*	*GlyNH*
**298**	8.05		8.48	7.96	7.73
**323**	7.96		8.3	7.85	7.78
Δppb/k					
**298-323**	3.6		7.2	4.4	-2

Characterization of compound **4**

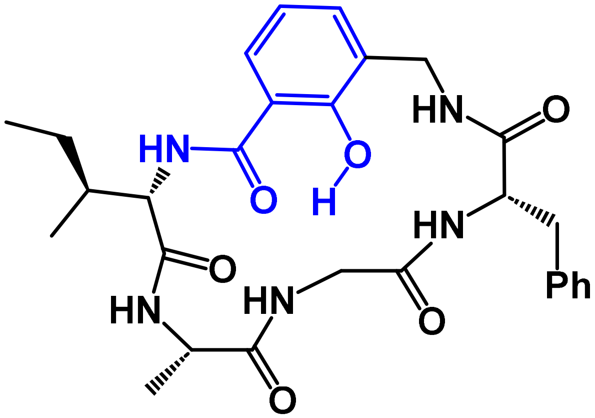

13.4 mg of a white solid obtained in a 25% overall yield (based on 100 % resin loading). ^{1}H NMR (600 MHz, dmso) δ 12.34 (s, 1H), 8.78 (d, *J* = 6.8 Hz, 1H), 8.30 (d, *J* = 8.0 Hz, 1H), 7.79 (d, *J* = 7.9 Hz, 1H), 7.76 – 7.73 (m, 2H), 7.69 (d, *J* = 7.6 Hz, 1H), 7.37 (d, *J* = 7.0 Hz, 1H), 7.25 (t, *J* = 7.3 Hz, 2H), 7.22 – 7.16 (m, 3H), 6.82 (t, *J* = 7.5 Hz, 1H), 4.38 (dd, *J* = 13.8, 8.2 Hz, 1H), 4.33 – 4.28 (m, 2H), 4.05 (d, *J* = 12.9 Hz, 1H), 3.93 (t, *J* = 7.6 Hz, 1H), 3.81 (dd, *J* = 16.6, 6.9 Hz, 1H), 3.24 (d, *J* = 12.9 Hz, 1H), 3.02 (dd, *J* = 13.8, 5.3 Hz, 1H), 2.73 (dd, *J* = 13.5, 9.7 Hz, 1H), 2.03 – 1.90 (m, 1H), 1.63 – 1.56 (m, 1H), 1.30 – 1.21 (m, 2H), 1.15 (d, *J* = 6.7 Hz, 3H), 0.93 – 0.84 (m, 7H). HRMS (ESI+) m/z calculated for C28 H36 N5 O6+ $[M+H]^{+}$ = 538.2676, found = 538.2660.

Figure S45:1H-NMR **4** (600 MHz, DMSO-*d*6)

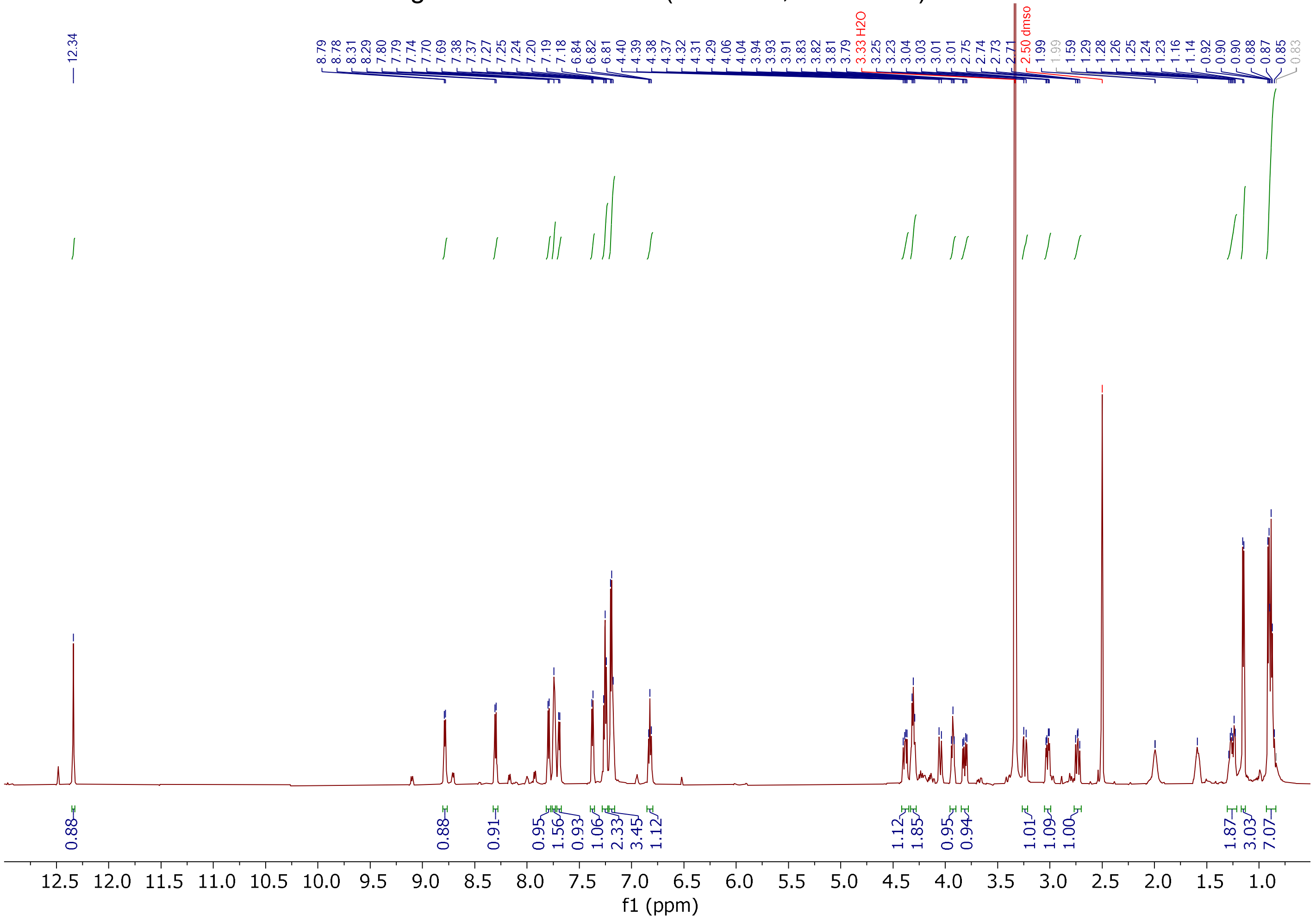

Figure S46: 2D-COSY Compound **4** (600 MHz, DMSO-*d*6)

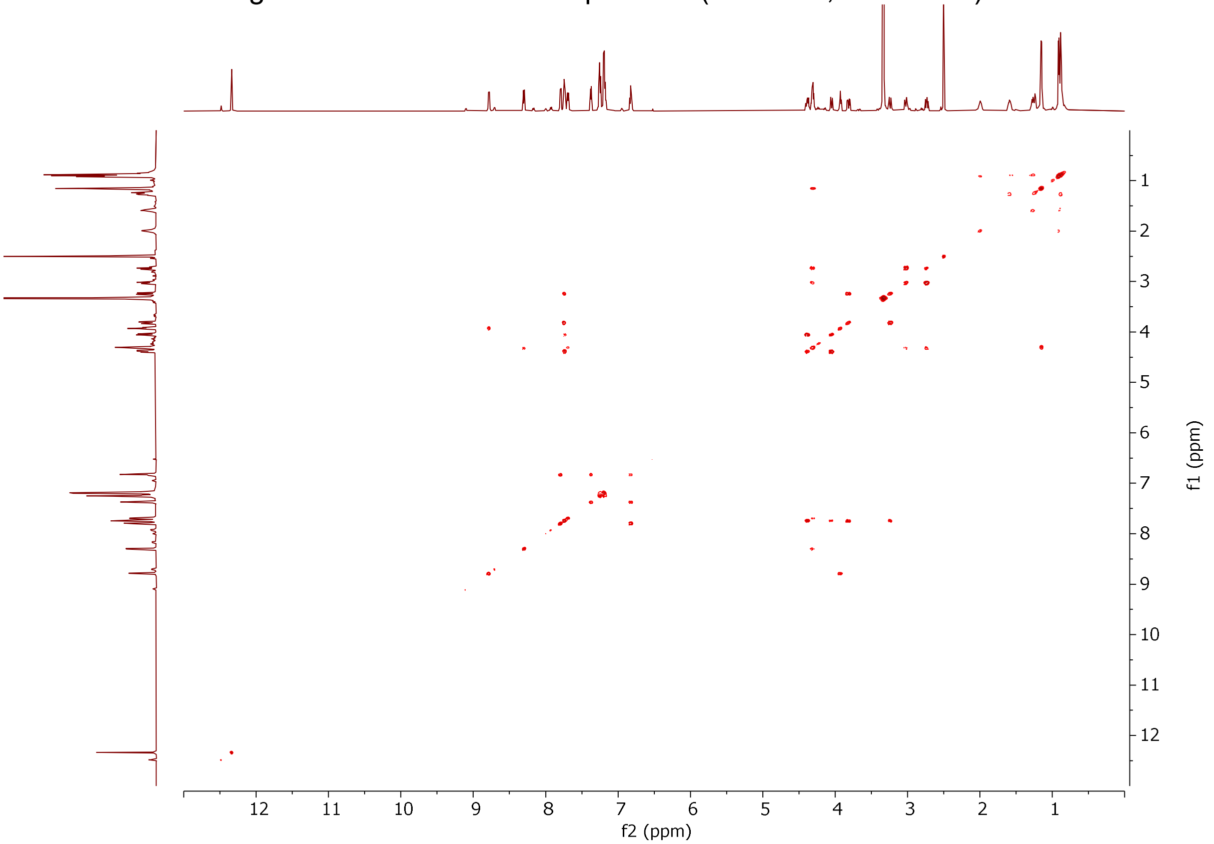

Figure S47: 2D-TOCSY Compound **4** (600 MHz, DMSO-*d*6)

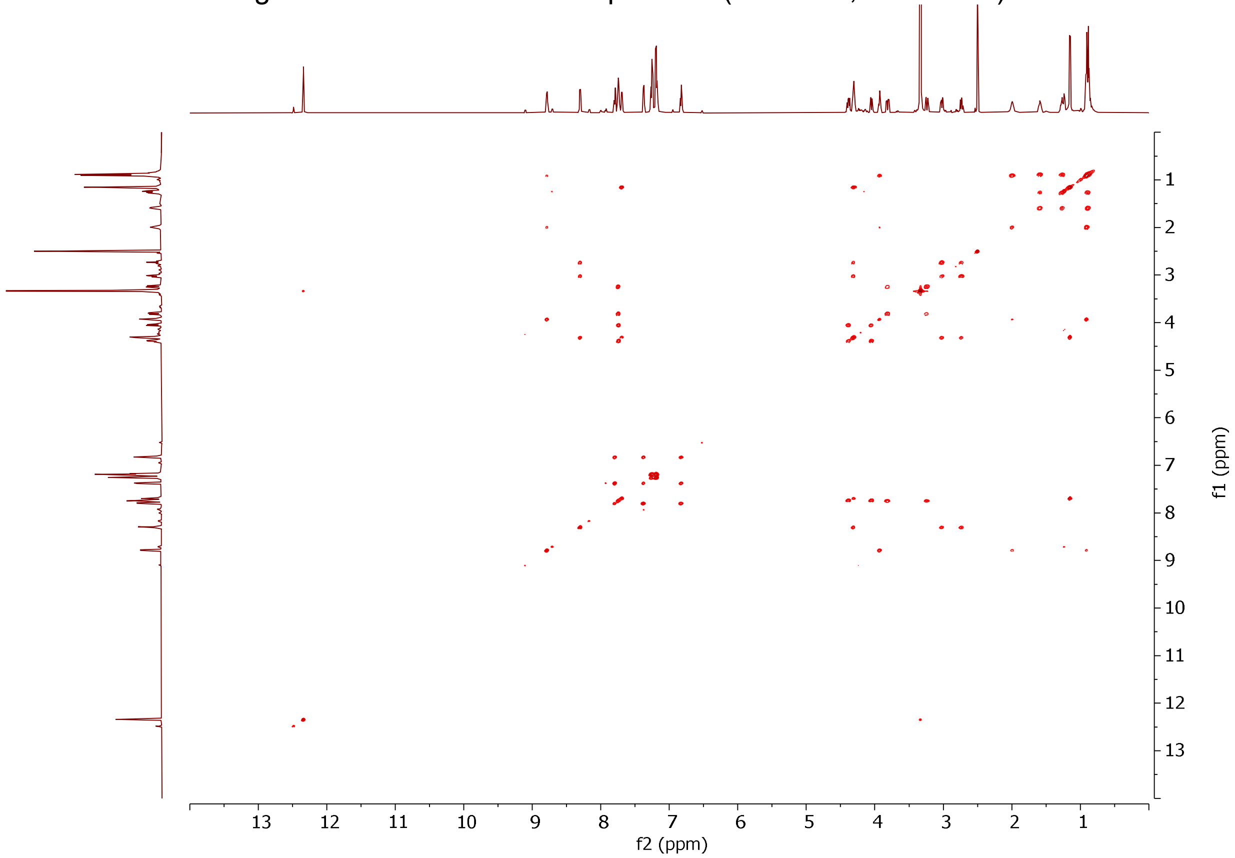

Figure S48: 2D-ROESY Compound **4** (600 MHz, DMSO-*d*6)

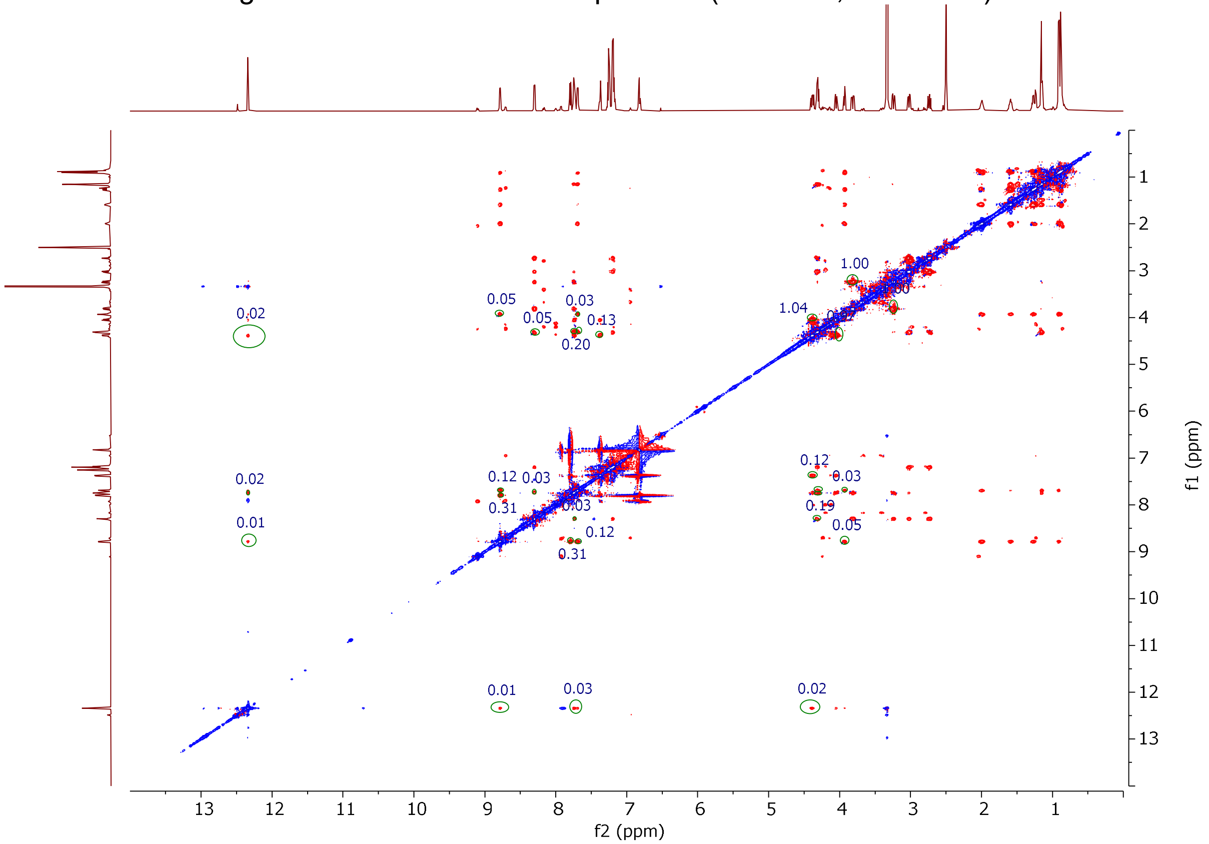

Figure S49: 2D-HSQC Compound **4** (600 MHz, DMSO-*d*6)

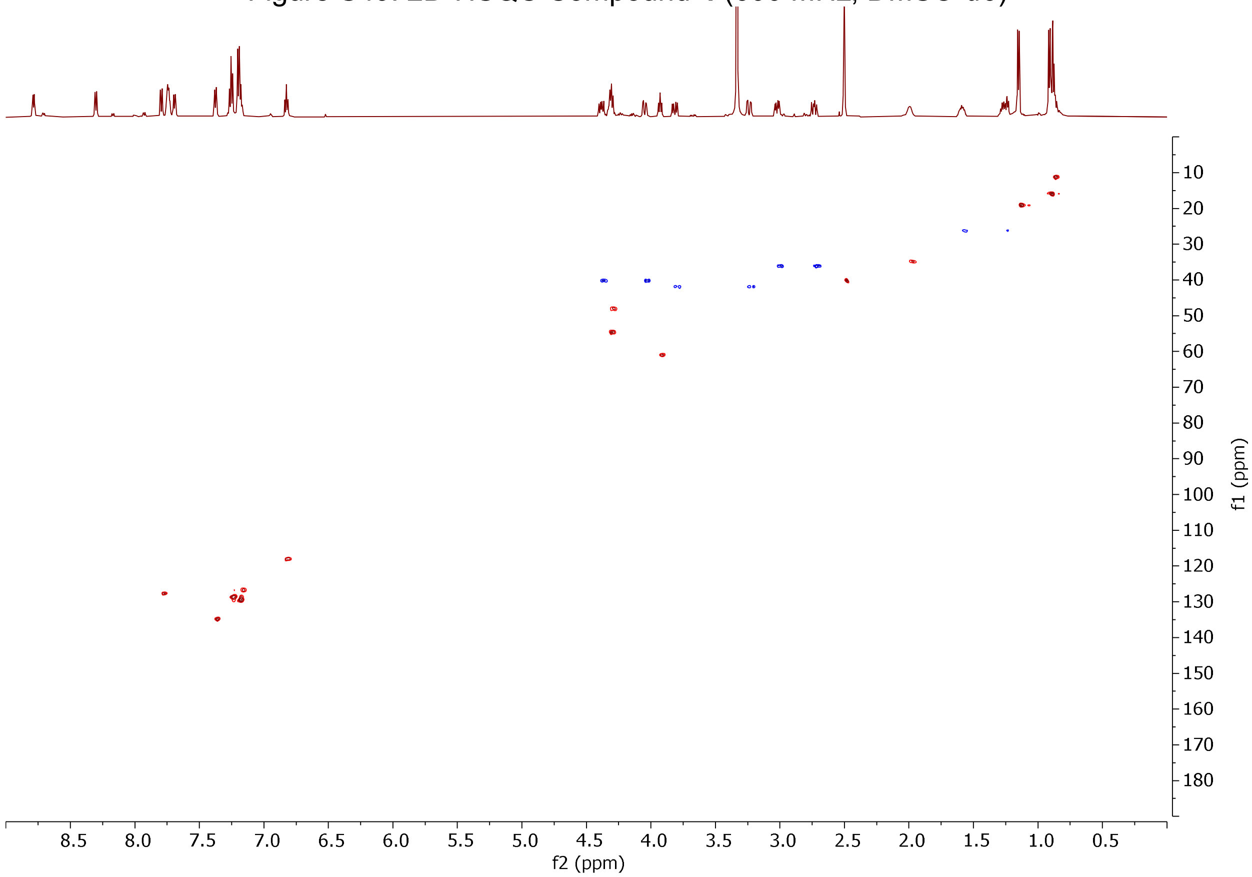

Figure S50: 2D-HMBC Compound **4** (600 MHz, DMSO-*d*6)

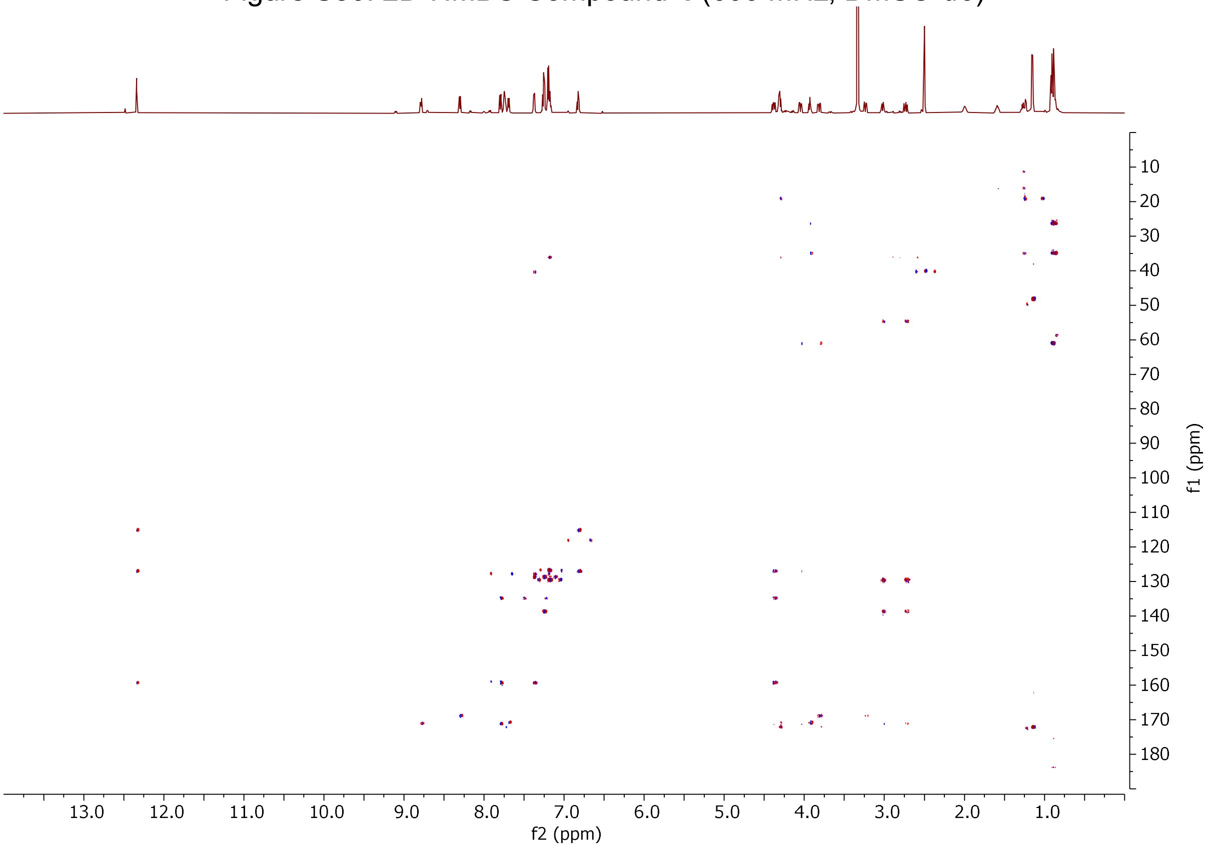

Figure S51: VT 1H-NMR Compound **4** (600 MHz, DMSO-*d*6)

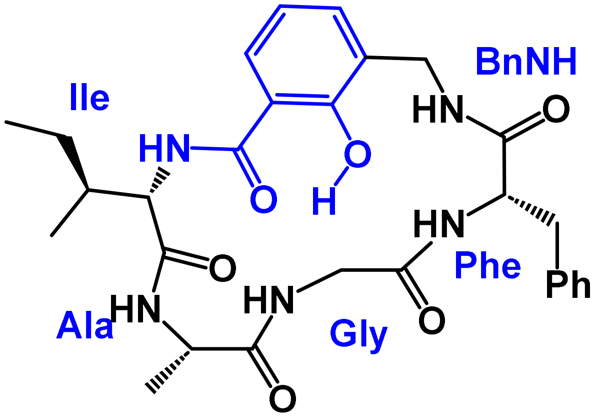

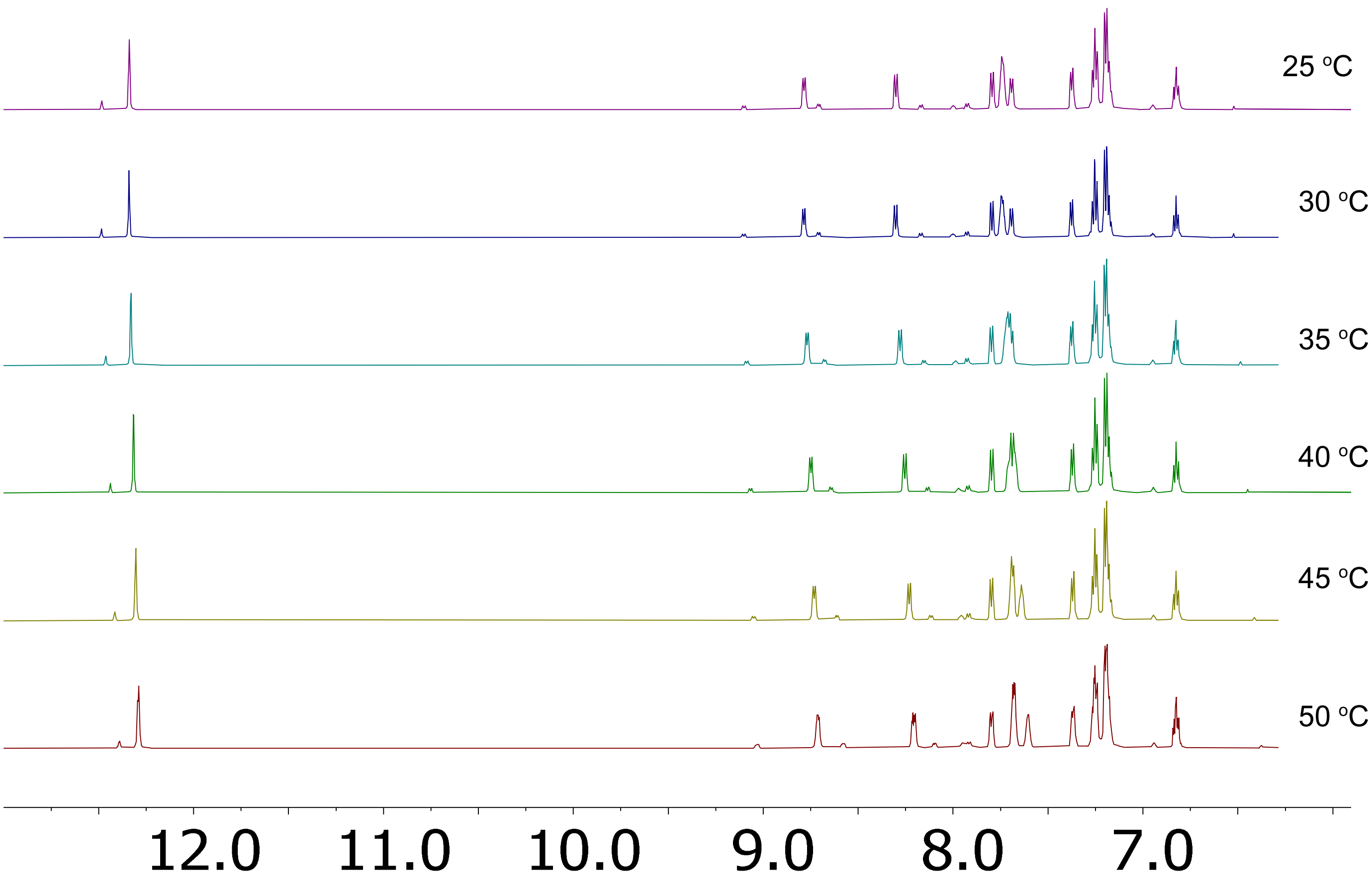

	Shifts (ppm)					
K	*AlaNH*	*Ile*	*BnNH*	*Phe*	*GlyNH*	ArOH
**298**	7.69	8.78	7.74	8.3	7.75	12.34
**323**	7.68	8.71	7.61	8.21	7.68	12.29
Δppb/k						
**298-323**	0.4	2.8	5.2	3.6	2.8	2

Characterization of compound **5**

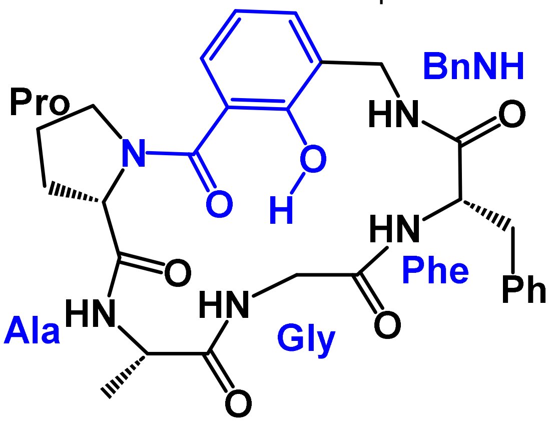

14.6 mg of a white solid obtained in a 28% overall yield (based on 100 % resin loading). 1H NMR (600 MHz, dmso) δ 8.71 (s, 1H), 8.15 – 8.02 (m, 2H), 7.43 (s, 1H), 7.37 – 7.12 (m, 7H), 6.85 (t, *J* = 7.6 Hz, 1H), 4.78 – 4.66 (m, 1H), 4.37 – 4.30 (m, 2H), 4.26 (s, 1H), 3.89 (d, *J* = 14.0 Hz, 1H), 3.79 (d, *J* = 11.0 Hz, 1H), 3.72 (s, 1H), 3.66 – 3.58 (m, 1H), 3.39 – 3.36 (m, 1H), 3.28 – 3.22 (m, 1H), 2.95 (s, 1H), 2.87 – 2.80 (m, 2H), 1.89 – 1.86 (m, 2H), 1.27 – 1.24 (m, 4H). HRMS (ESI+) m/z calculated for C27 H32 N5 O6+ $[M+H]^+$ = 522.2359, found = 522.2347.

Figure S52:1H-NMR **4** (600 MHz, DMSO-*d6*)

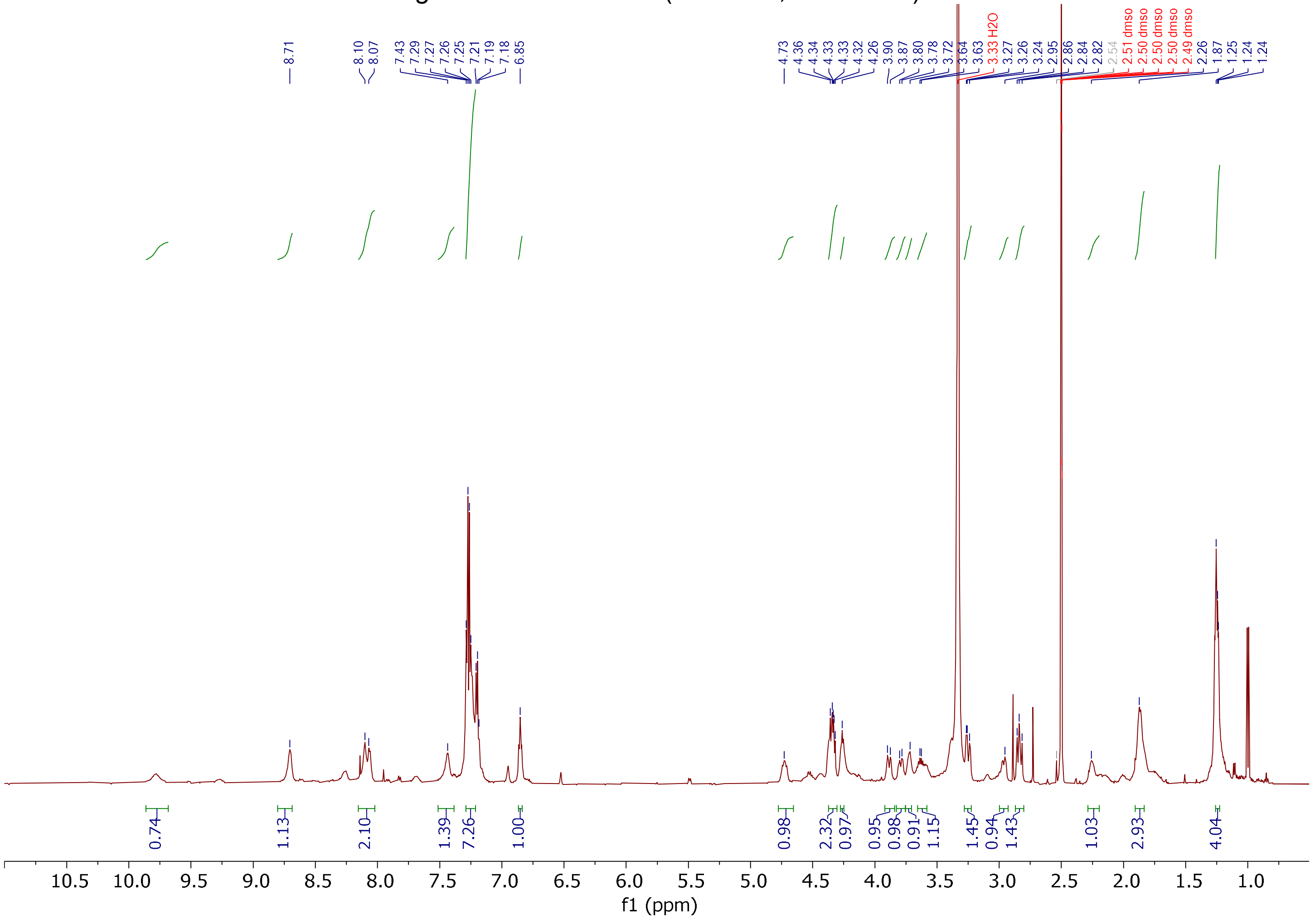

Figure S53: 2D-COSY Compound **5** (600 MHz, DMSO-*d*6)

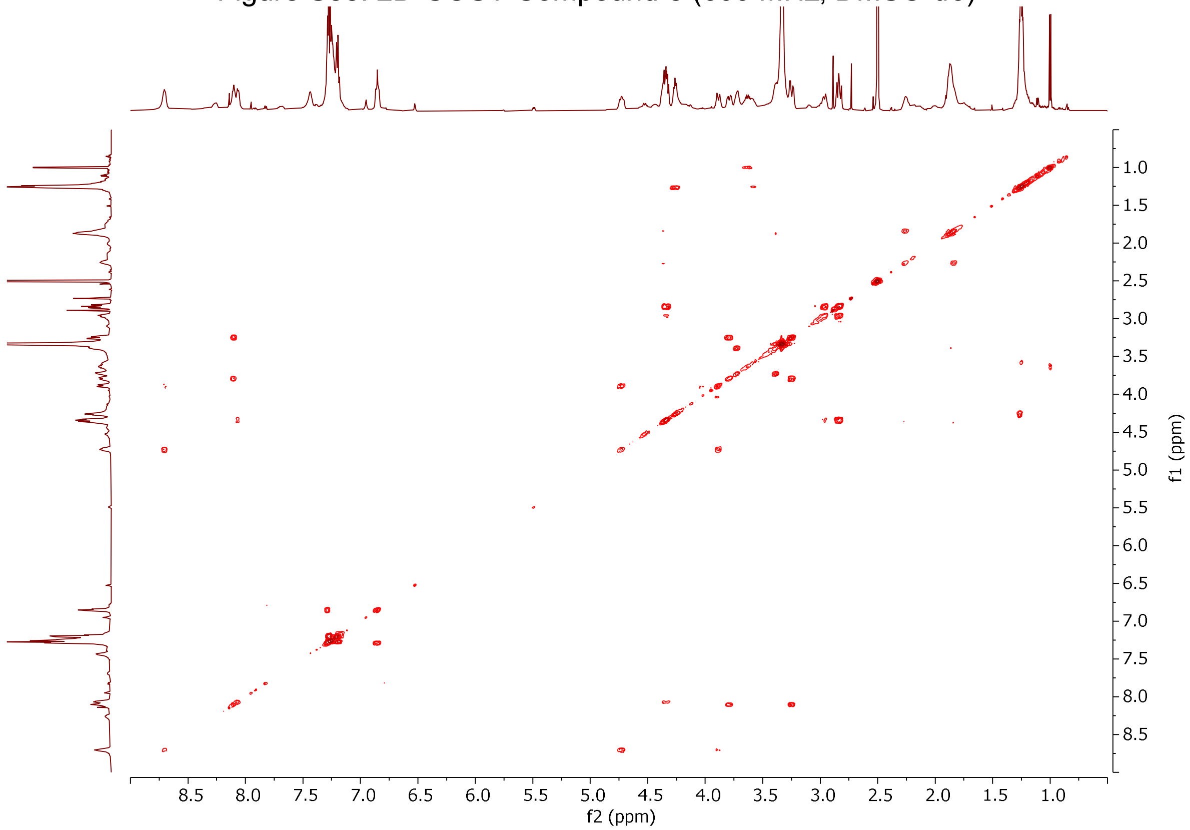

Figure S54: 2D-TOCSY Compound **5** (600 MHz, DMSO-*d*6)

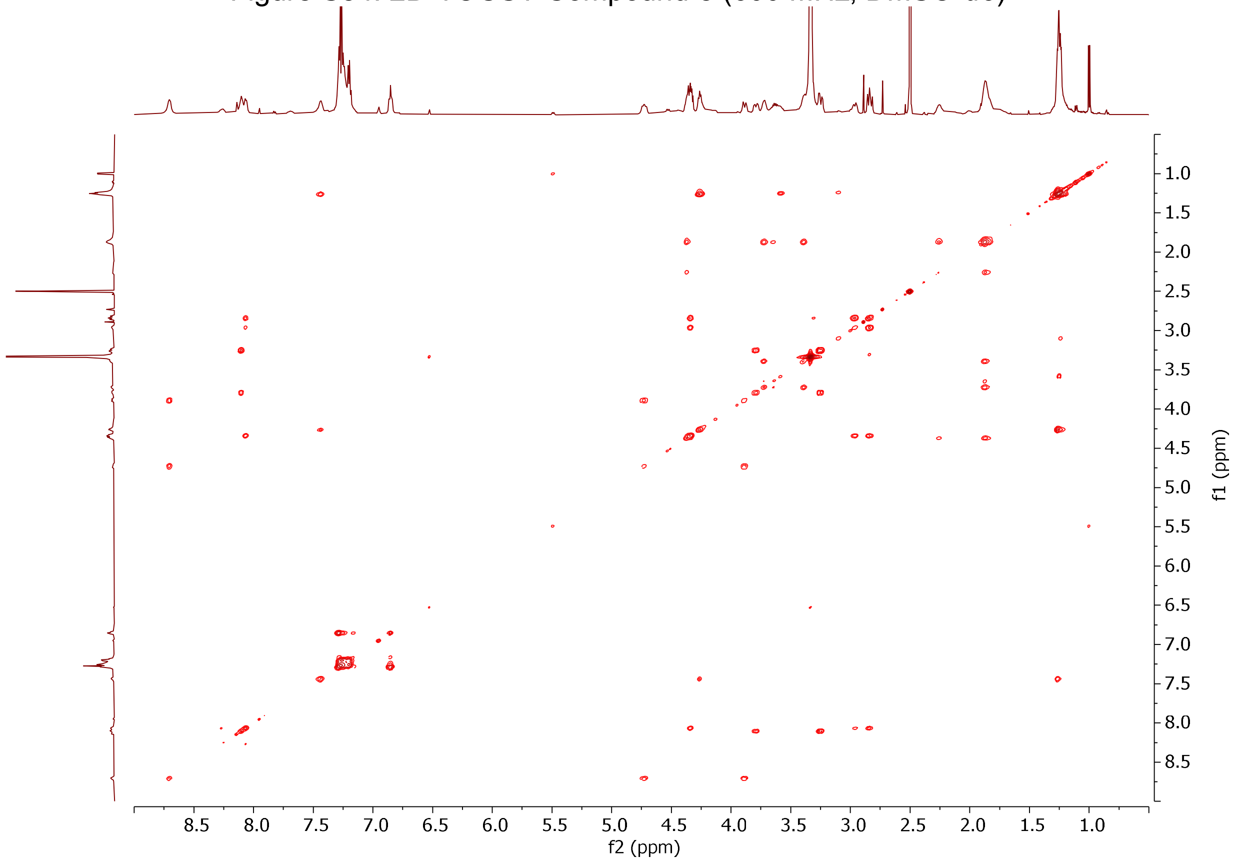

Figure S55: 2D-ROESY Compound **5** (600 MHz, DMSO-*d*6)

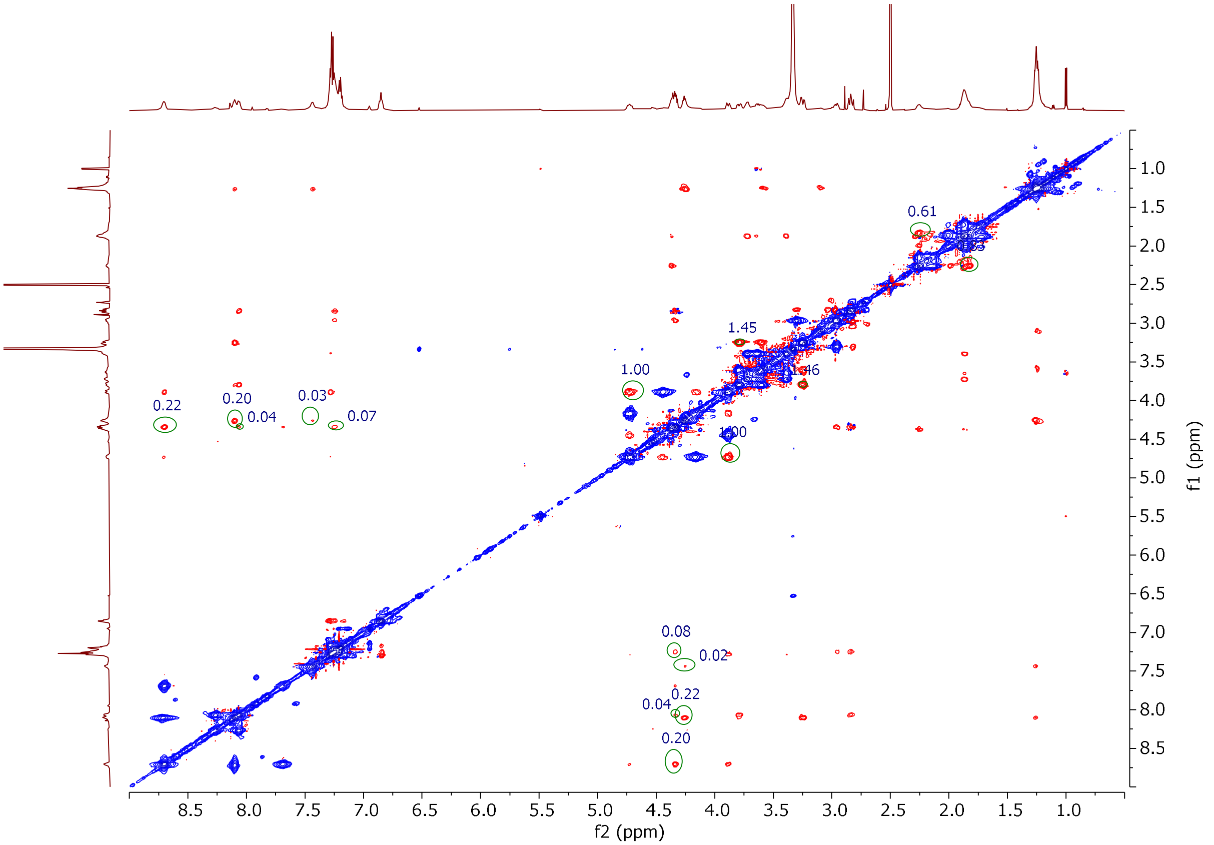

Figure S56: 2D-HSQC Compound **5** (600 MHz, DMSO-*d*6)

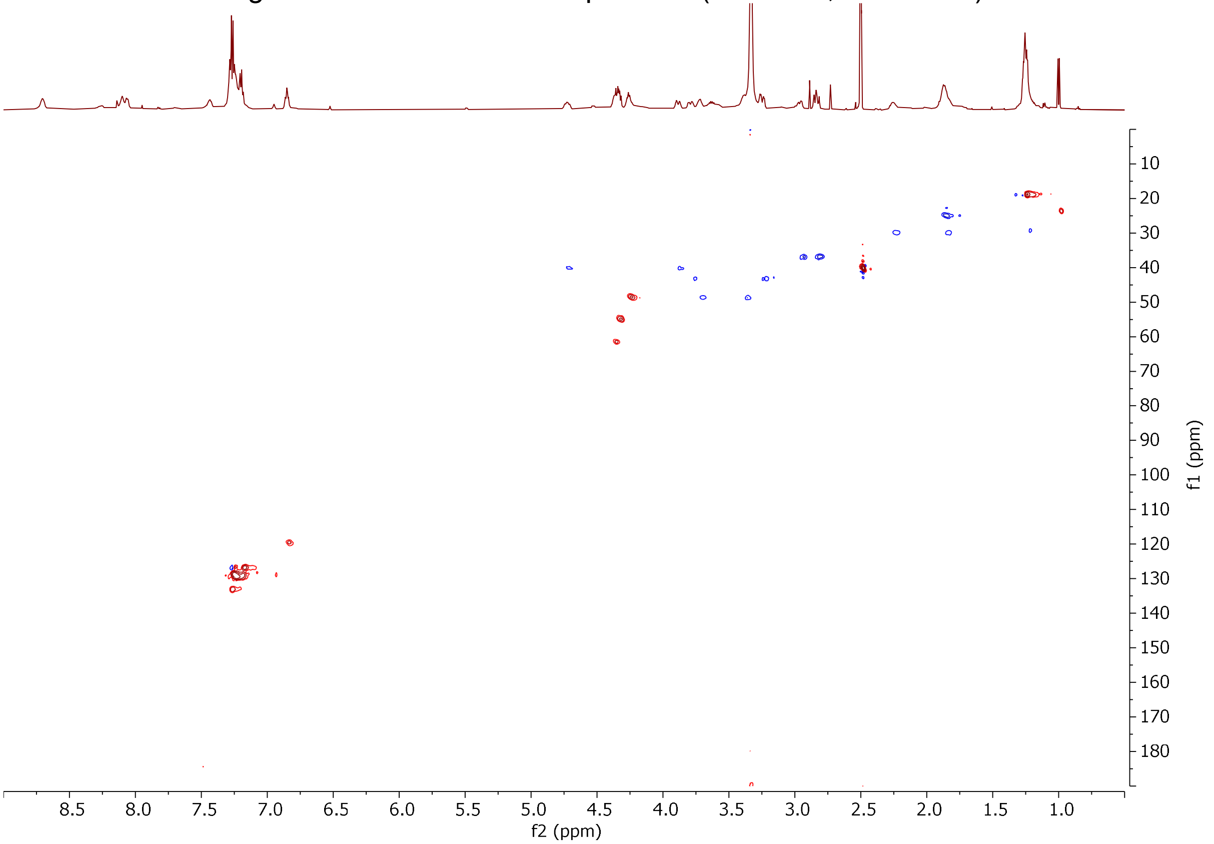

Figure S57: VT 1H-NMR Compound **5** (600 MHz, DMSO-*d*6)

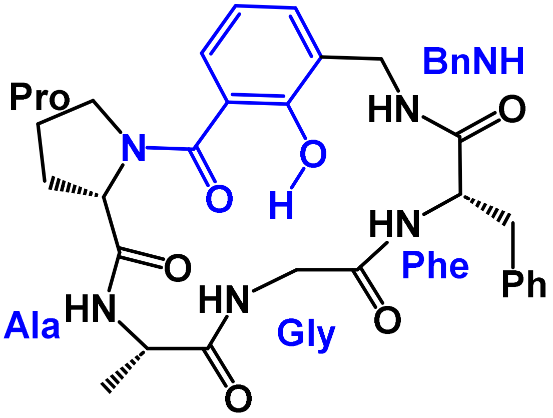

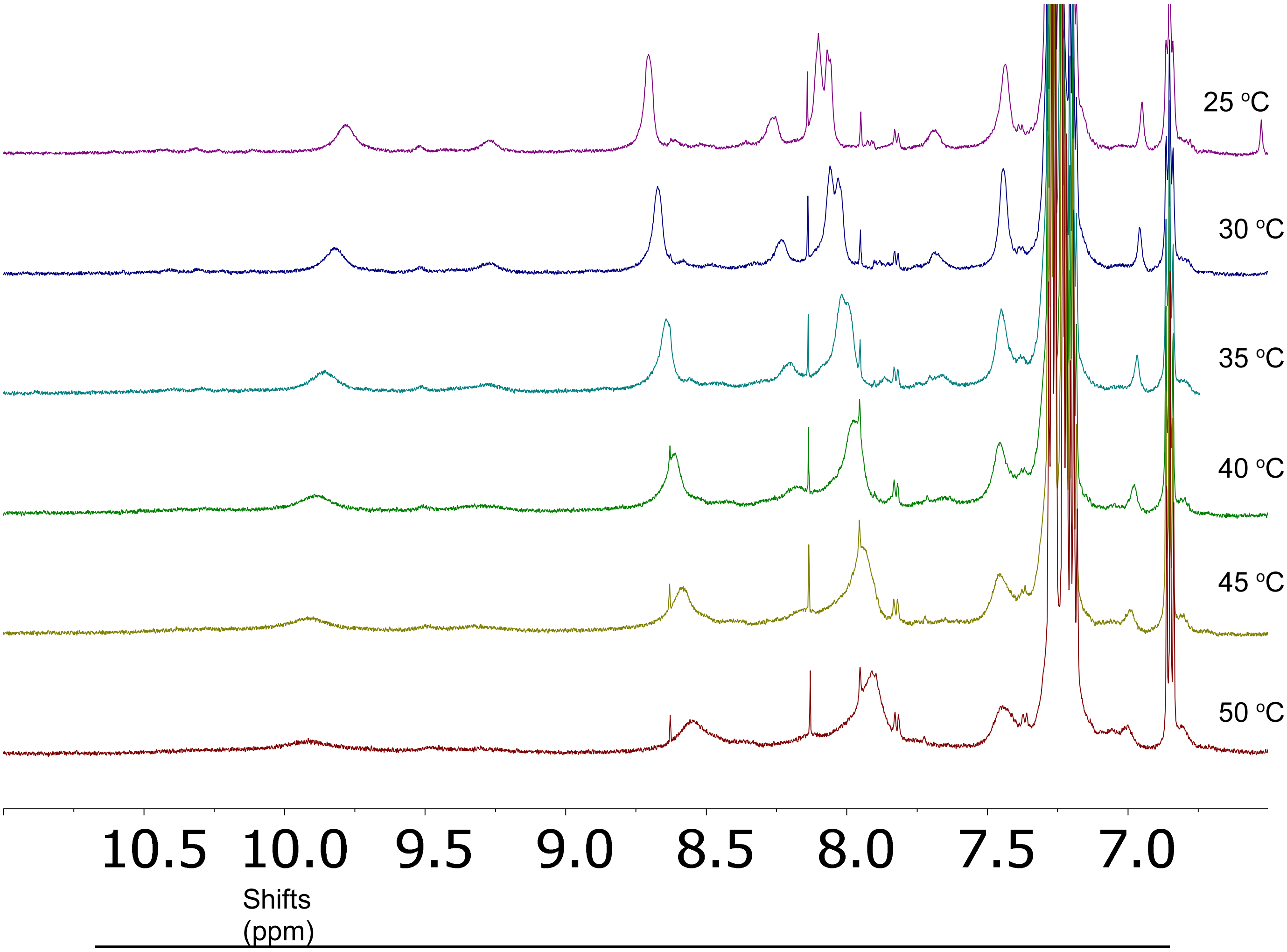

K	*AlaNH*	*Pro*	*BnNH*	*Phe*	*GlyNH*	ArOH
**298**	7.44		8.7	8.06	8.1	9.78
**323**	7.44		8.55	7.92	7.92	9.92
Δppb/k						
**298-323**	0		6	5.6	7.2	-5.6

Characterization of compound **6**

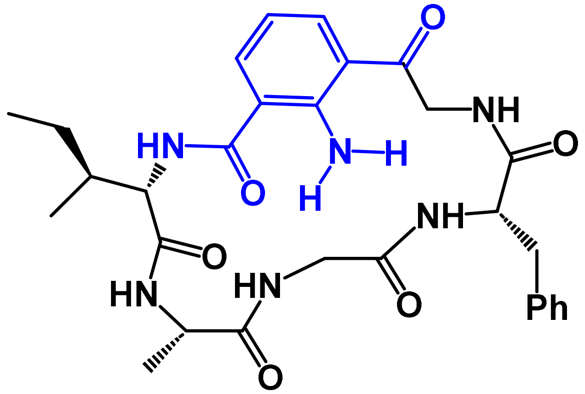

9.6 mg of an off-white/yellowish solid obtained in a 17% overall yield (based on 100 % resin loading). ^{1}H NMR (500 MHz, dmso) δ 8.60 (s, 1H), 8.48 (s, 1H), 8.19 (d, *J* = 13.0 Hz, 1H), 7.79 (d, *J* = 7.5 Hz, 1H), 7.57 (d, *J* = 7.1 Hz, 1H), 7.26 – 7.11 (m, 7H), 7.09 (s, 2H), 6.58 (t, *J* = 7.7 Hz, 1H), 4.76 – 4.69 (m, 1H), 4.33 – 4.26 (m, 1H), 4.04 (t, *J* = 8.3 Hz, 1H), 3.70 (td, *J* = 14.3, 4.8 Hz, 2H), 3.46 – 3.38 (m, 1H), 2.88 (dd, *J* = 13.8, 4.3 Hz, 1H), 2.64 (dd, *J* = 13.7, 10.6 Hz, 1H), 1.99 (d, *J* = 4.8 Hz, 1H), 1.60 – 1.53 (m, 1H), 1.29 – 1.18 (m, 5H), 0.93 – 0.86 (m, 6H). HRMS (ESI+) m/z calculated for C29 H37 N6 O6+ $[M+H]^+$ = 565.2776, found = 565.2769.

Figure S58:1H-NMR **6** (500 MHz, DMSO-*d*6)

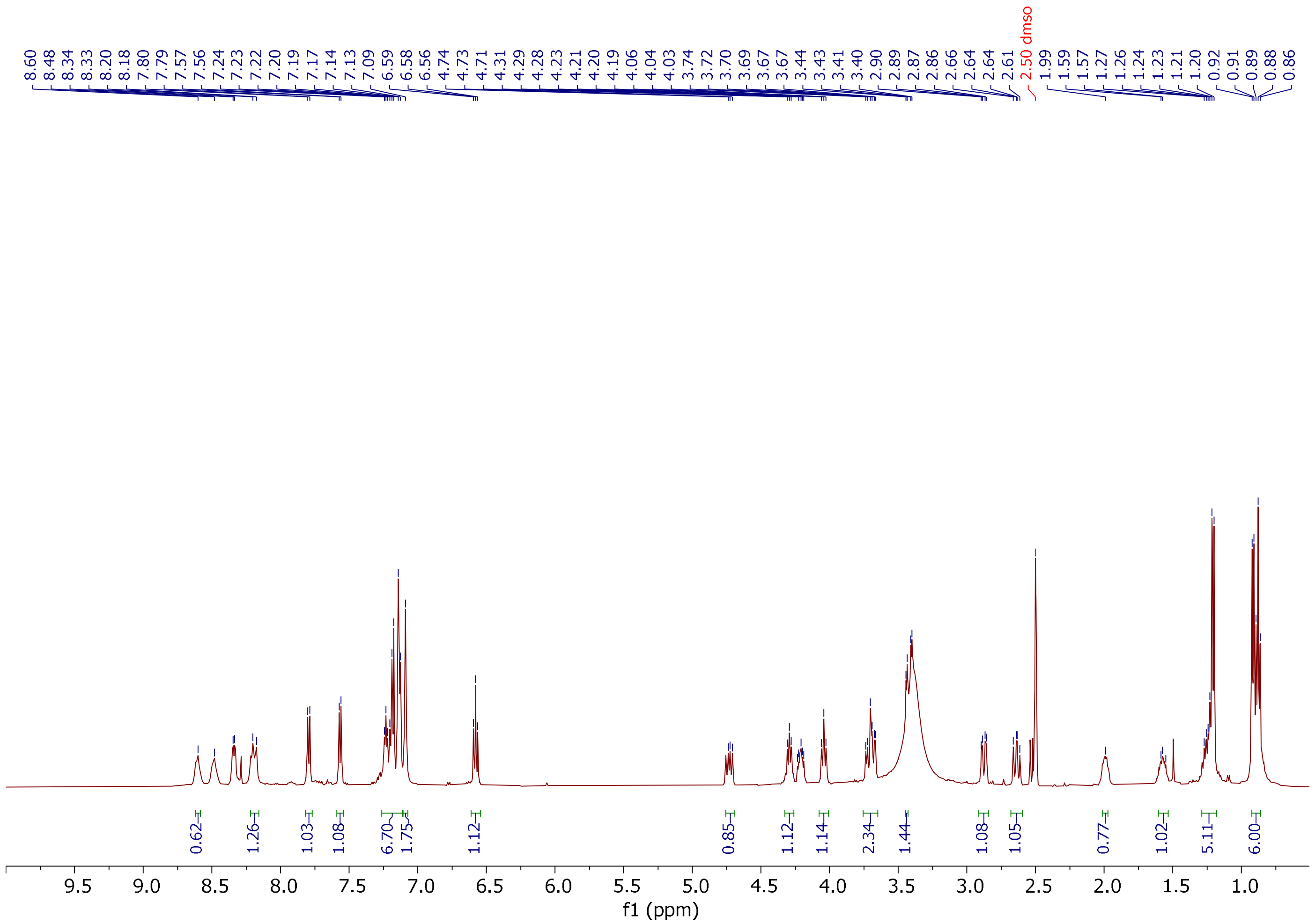

Figure S59: 2D-COSY Compound **6** (500 MHz, DMSO-*d*6)

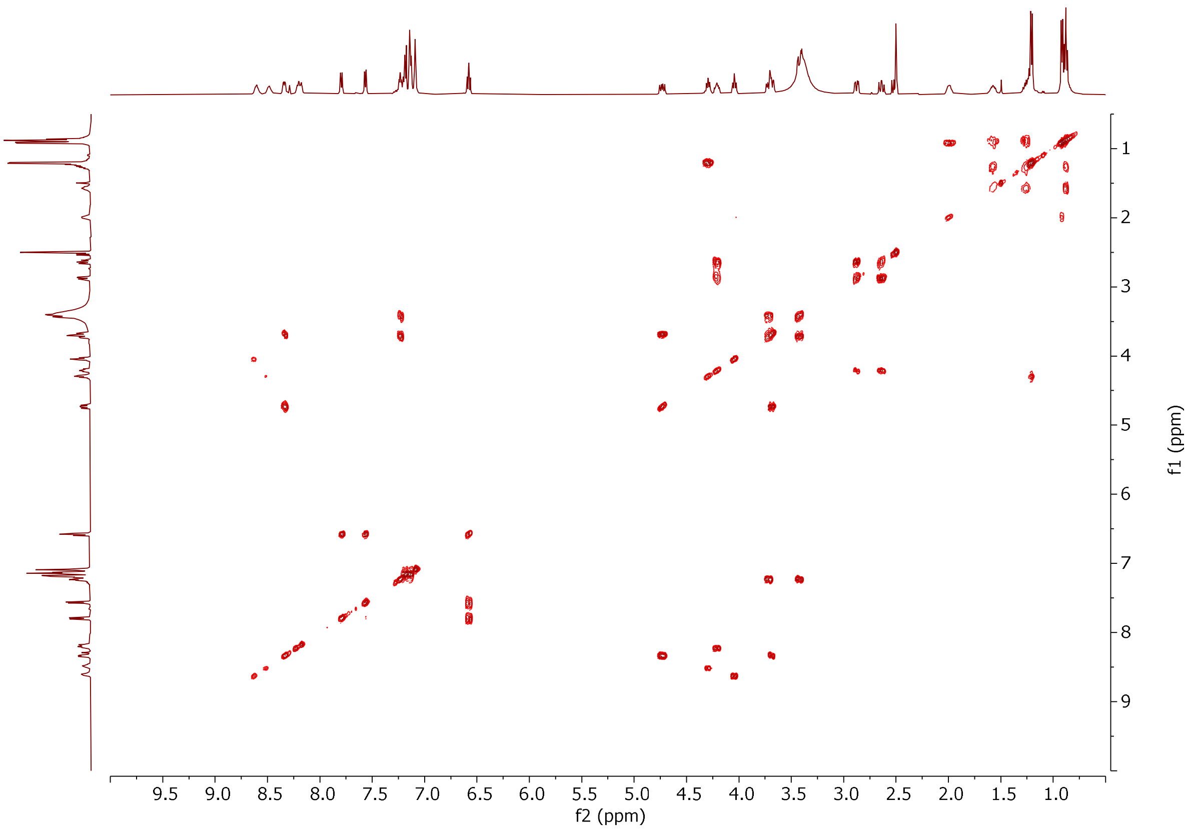

Figure S60: 2D-TOCSY Compound **6** (500 MHz, DMSO-*d*6)

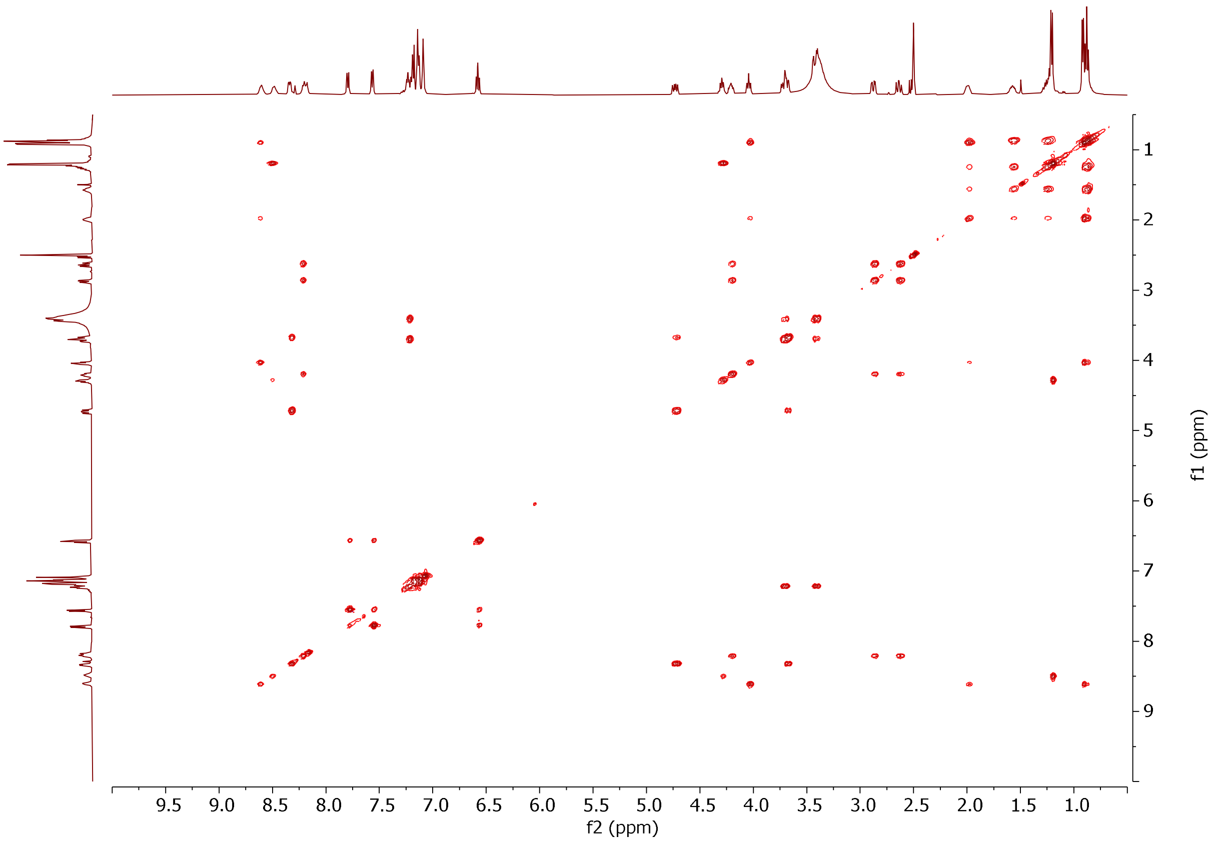

Figure S61: 2D-ROESY Compound **6** (500 MHz, DMSO-*d*6)

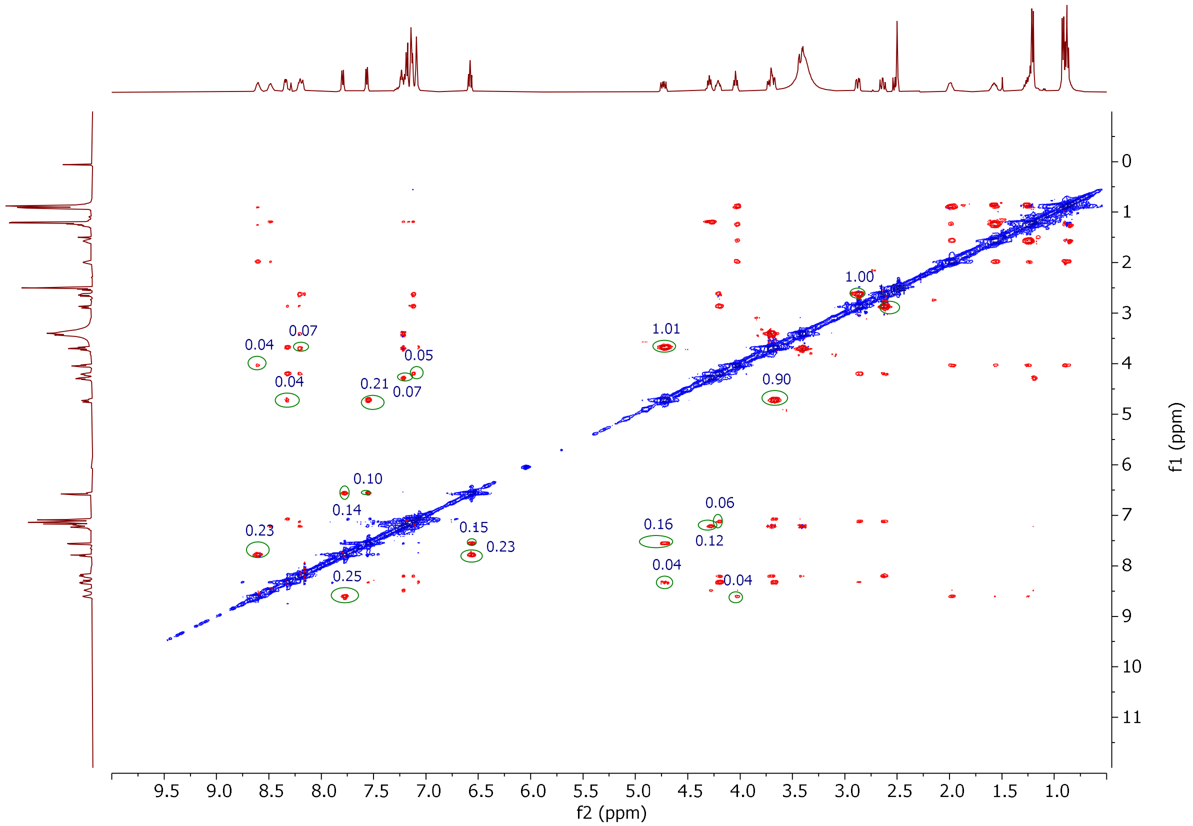

Figure S62: 2D-HSQC Compound **6** (500 MHz, DMSO-*d*6)

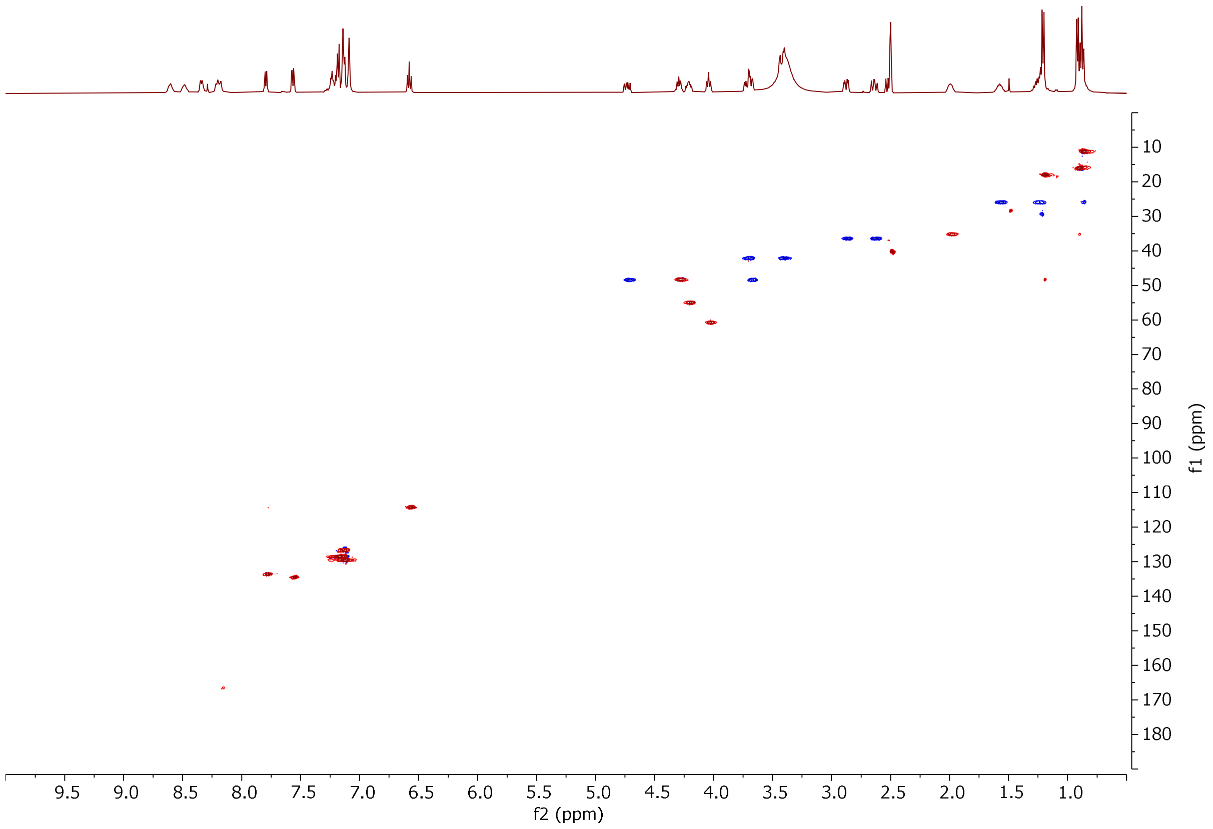

Figure S63: 2D-HMBC Compound **5** (500 MHz, DMSO-*d*6)

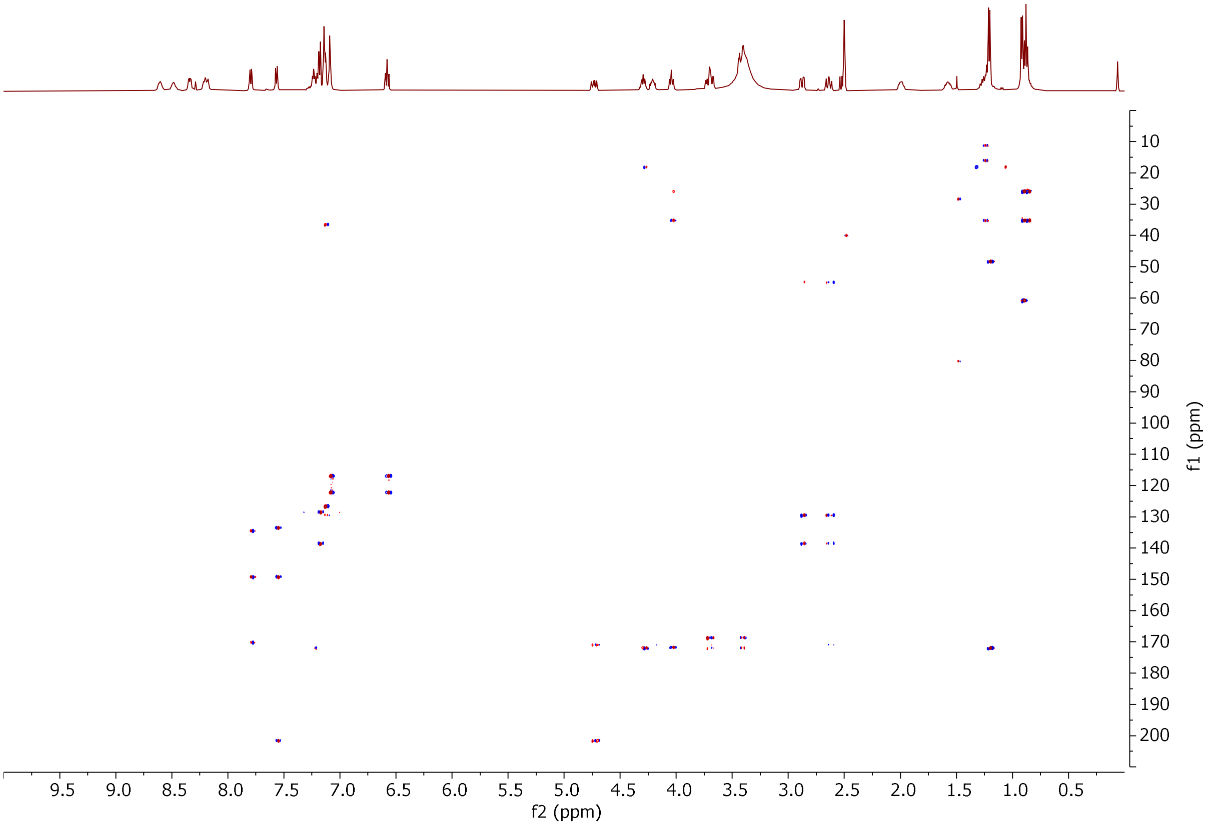

Figure S64: VT 1H-NMR Compound **6** (500 MHz, DMSO-*d*6)

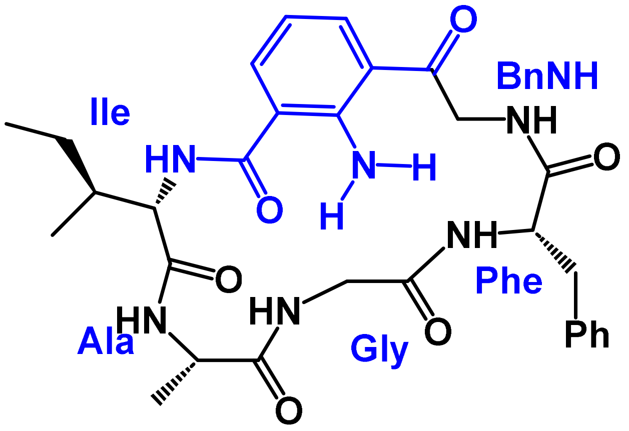

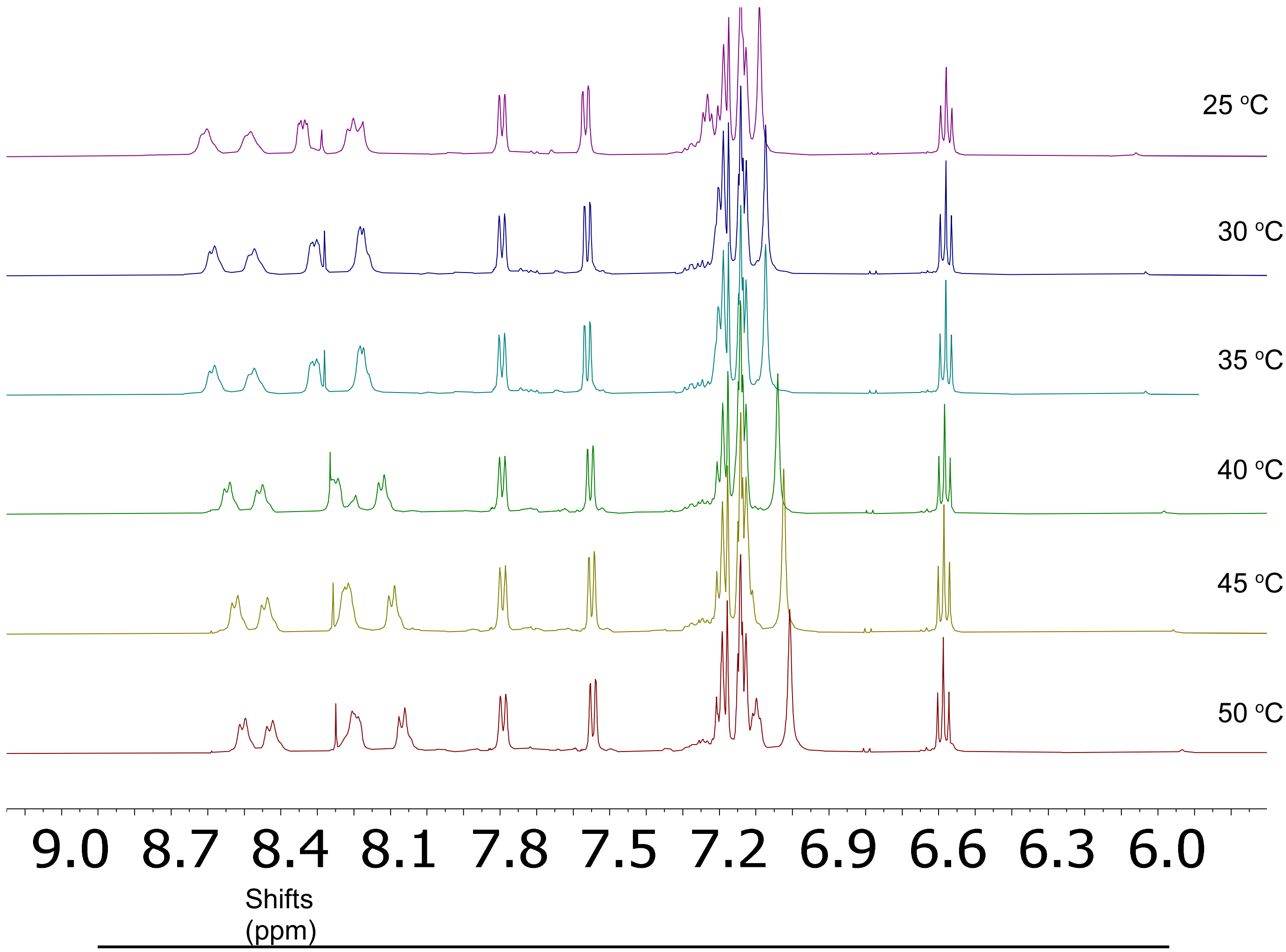

K	*AlaNH*	*Ile*	*BnNH*	*Phe*	*GlyNH*	$ArNH_2$
**298**	8.47	8.59	8.32	8.18	7.21	7.07
**323**	8.41	8.48	8.18	8.04	7.08	6.99
Δppb/k						
**298-323**	2.4	4.4	5.6	5.6	5.2	3.2

Characterization of compound **7**

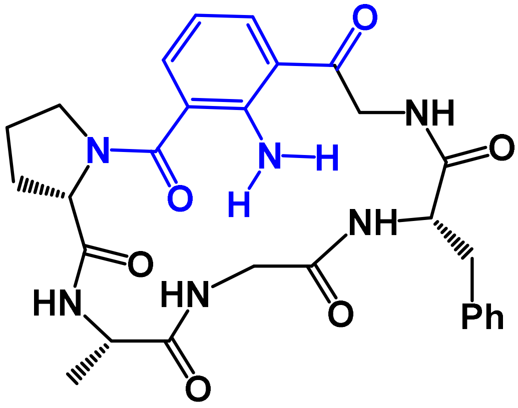

11.5 mg of an off-white/yellowish solid obtained in a 21% overall yield (based on 100 % resin loading). ^{1}H NMR (500 MHz, dmso) δ 8.96 (d, *J* = 9.9 Hz, 1H), 8.58 (d, *J* = 8.8 Hz, 1H), 8.34 (d, *J* = 7.7 Hz, 1H), 7.39 (d, *J* = 8.0 Hz, 1H), 7.26 (t, *J* = 7.6 Hz, 3H), 7.23 – 7.15 (m, 3H), 6.95 (s, 1H), 6.71 (s, 1H), 6.37 (t, *J* = 7.7 Hz, 1H), 5.24 (t, *J* = 12.4 Hz, 1H), 4.42 (q, *J* = 8.2 Hz, 1H), 4.21 (t, *J* = 7.3 Hz, 1H), 4.02 (d, *J* = 8.4 Hz, 1H), 3.72 – 3.61 (m, 2H), 3.55 (t, *J* = 10.0 Hz, 1H), 3.42 (dd, *J* = 17.4, 4.0 Hz, 2H), 3.00 (dd, *J* = 13.8, 7.3 Hz, 1H), 2.87 – 2.79 (m, 2H), 2.16 – 2.12 (m, 1H), 1.99 – 1.93 (m, 1H), 1.90 – 1.87 (m, 1H), 1.65 (s, 1H), 1.19 (d, *J* = 7.2 Hz, 3H). HRMS (ESI+) m/z calculated for C28 H33 N6 O6+ $[M+H]^+$ = 549.247, found = 549.2456.

Figure S65:1H-NMR **7** (500 MHz, DMSO-*d*6)

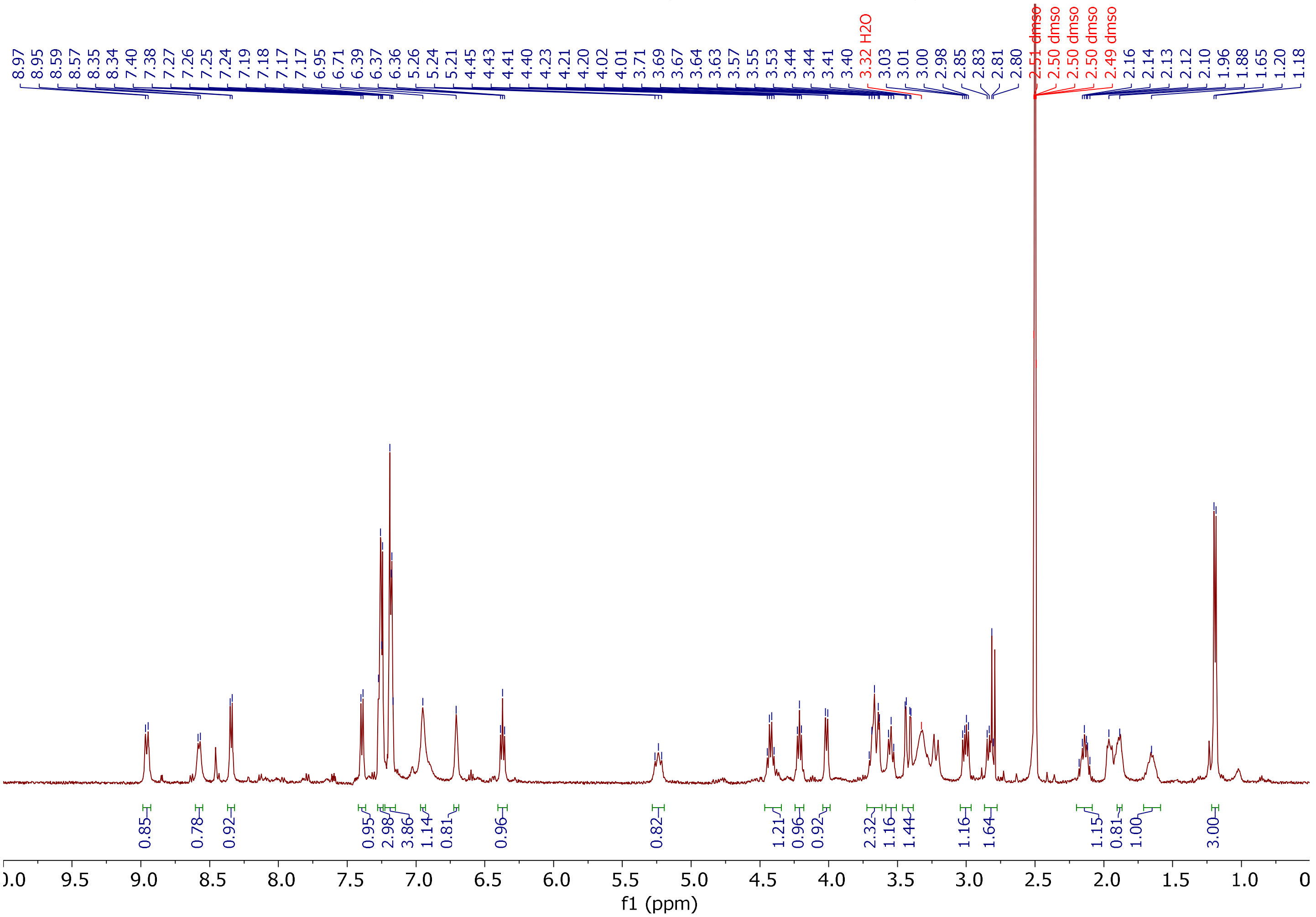

Figure S66: 2D-COSY Compound **7** (500 MHz, DMSO-*d*6)

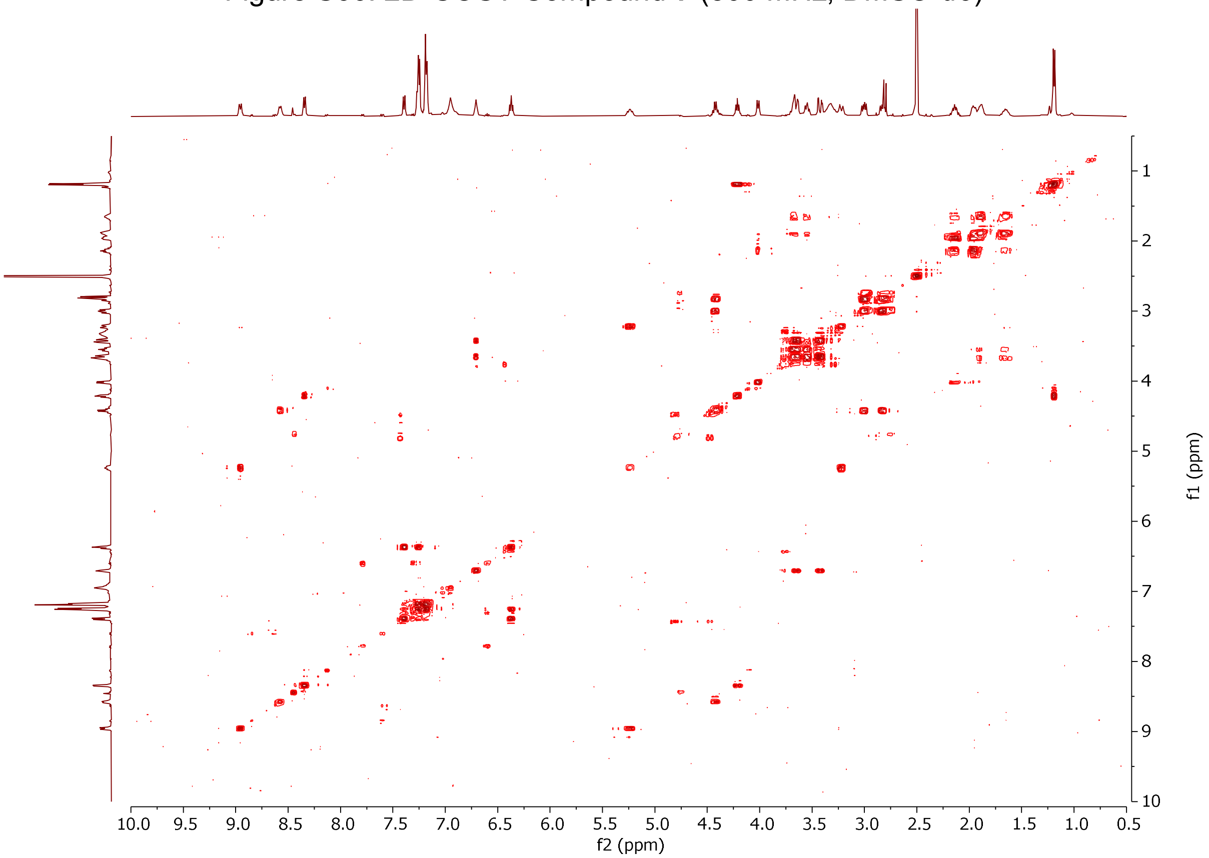

Figure S67: 2D-TOCSY Compound **7** (500 MHz, DMSO-*d*6)

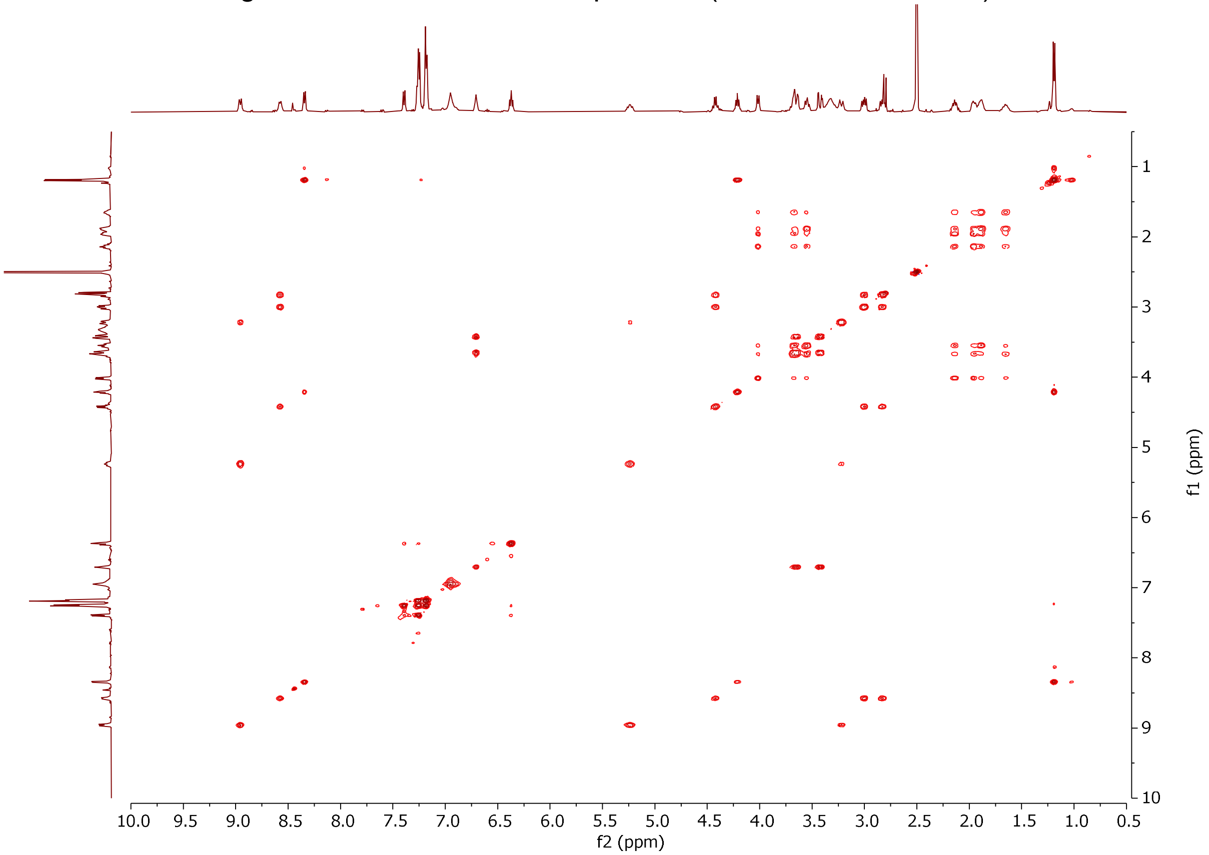

Figure S68: 2D-ROESY Compound **7** (500 MHz, DMSO-*d*6)

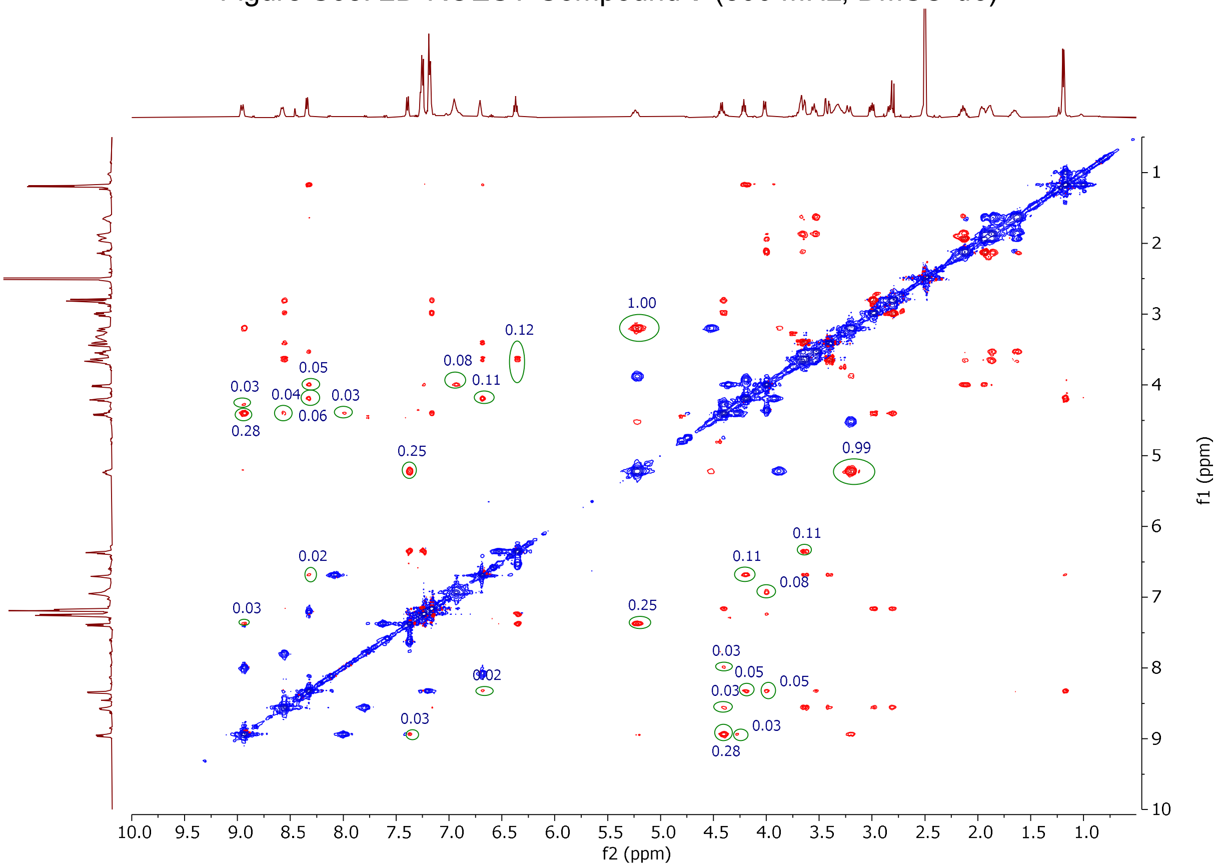

Figure S69: 2D-HSQC Compound **7** (500 MHz, DMSO-*d*6)

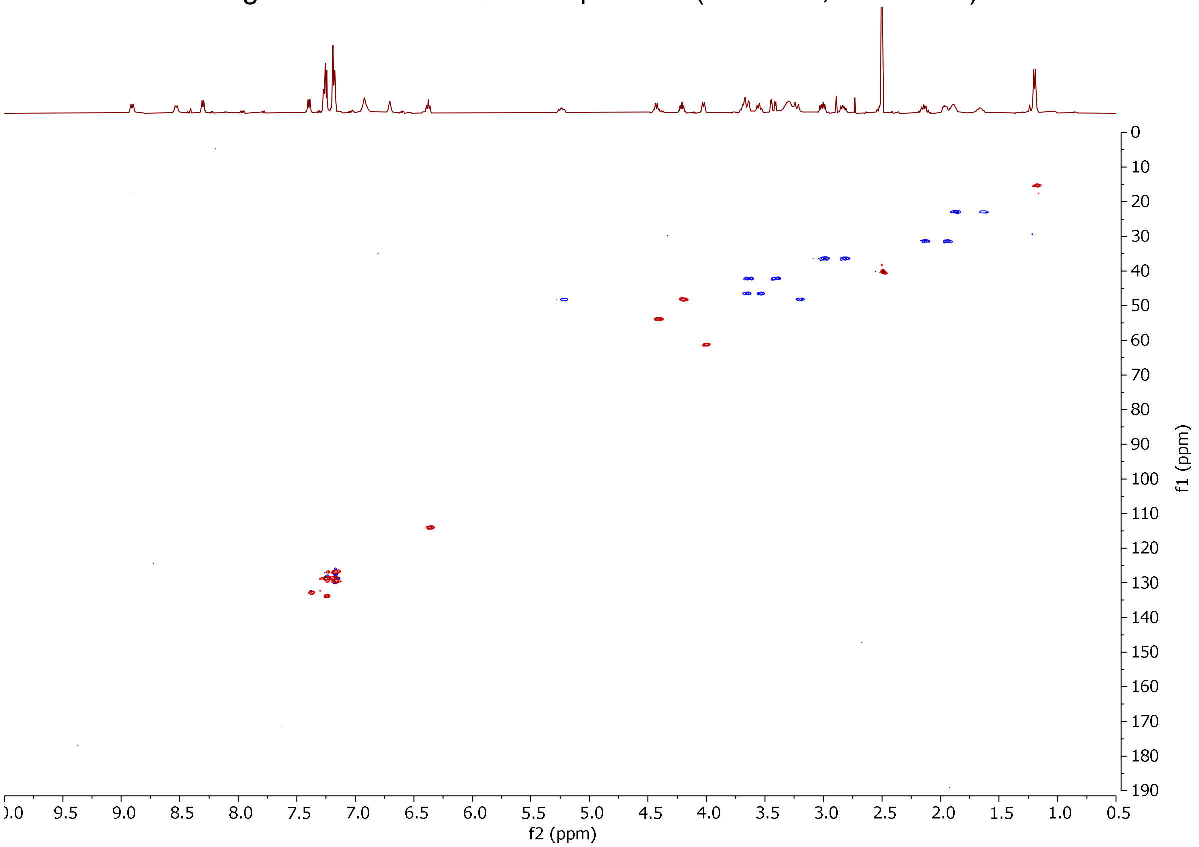

Figure S70: 2D-HMBC Compound **7** (500 MHz, DMSO-*d*6)

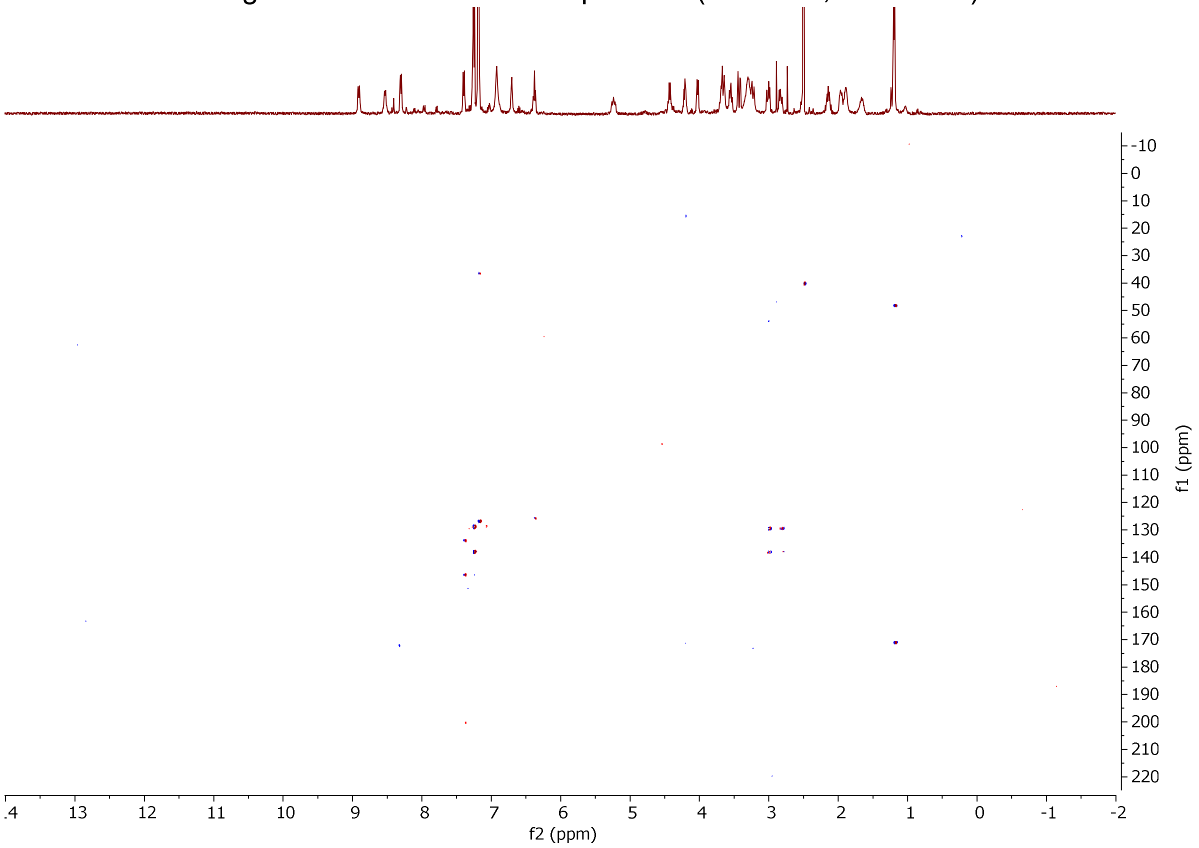

Figure S71: VT 1H-NMR Compound **7** (500 MHz, DMSO-*d*6)

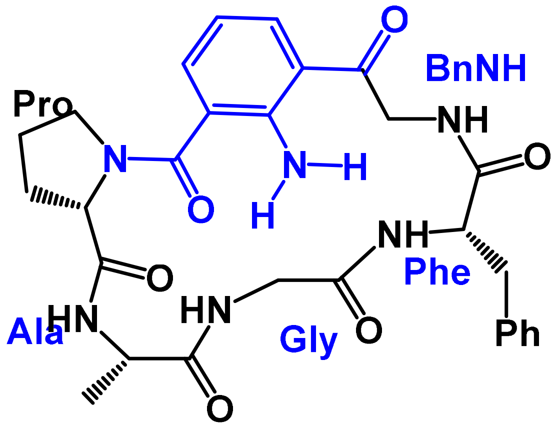

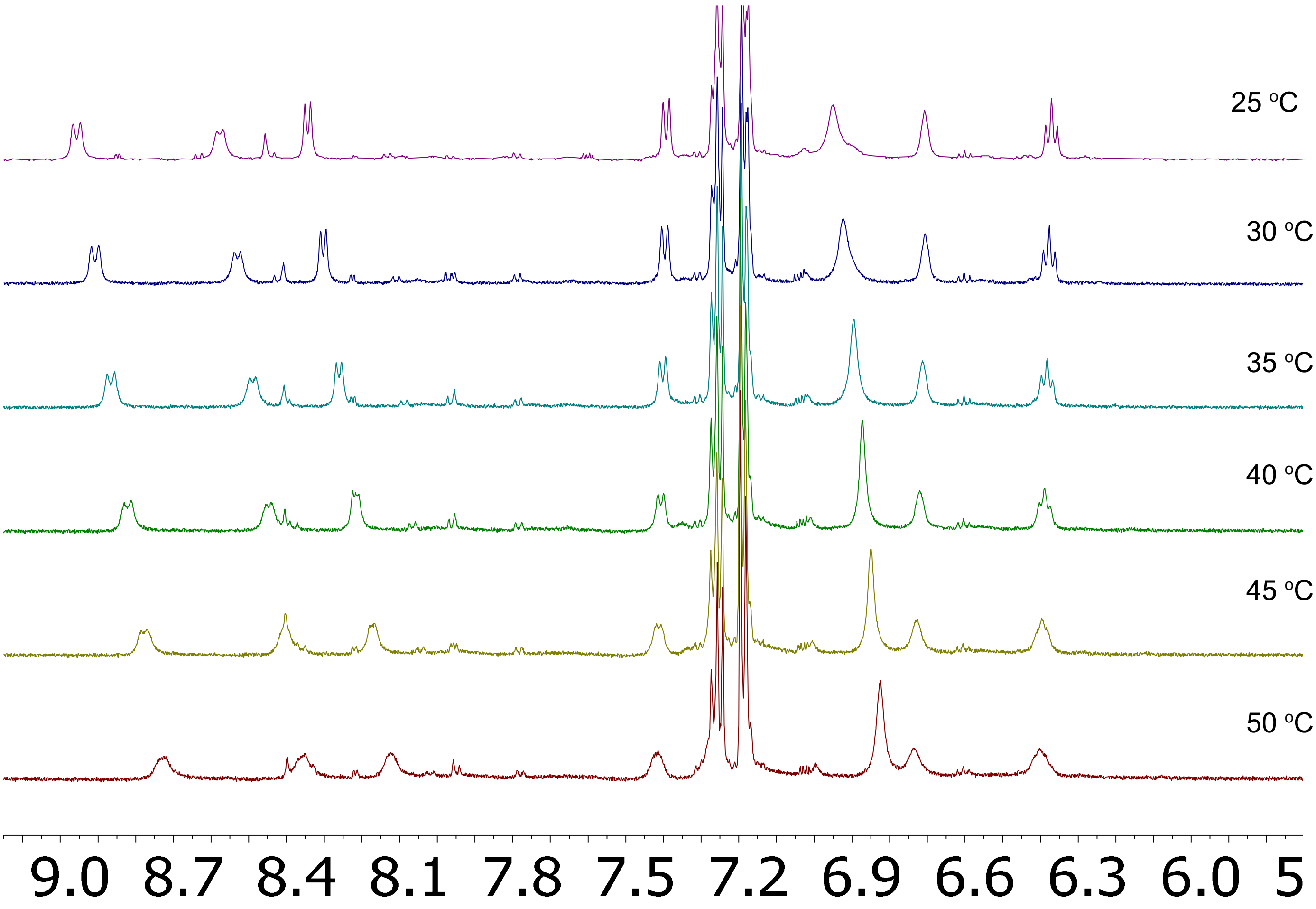

	Shifts (ppm)					
K	*AlaNH*	*Pro*	*BnNH*	*Phe*	*GlyNH*	$ArNH_2$
**298**	8.34		8.96	8.58	6.71	6.94
**323**	8.13		8.72	8.36	6.73	6.80
Δppb/k						
**298-323**	7		8	7.33	-0.667	4.67

LC Traces

Figure S72: **Crude overnight cyclization of PFP ester – S22a:** (5-95 %, MeCN/H_2O with 0.1 % formic acid over 15 min)

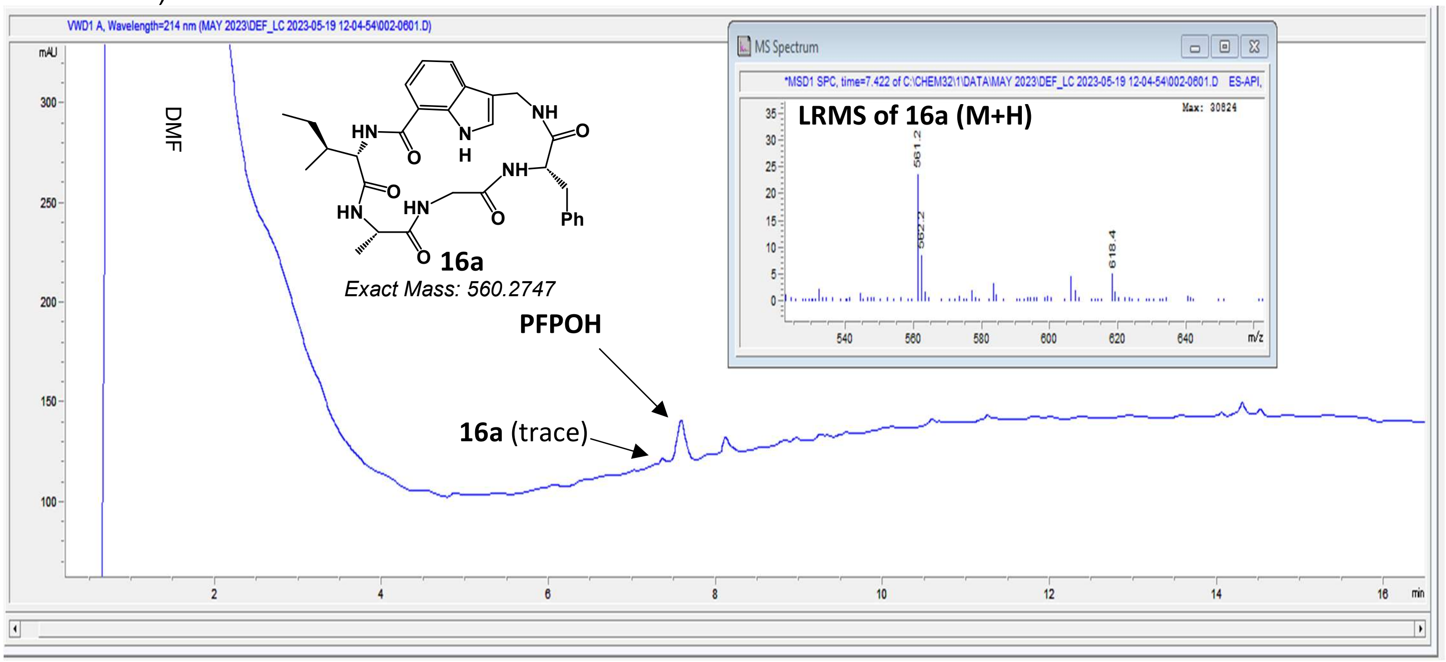

Figure S73: **Crude overnight cyclization of PFP ester – S22b:** (5-95 %, MeCN/H_2O with 0.1 % formic acid over 15 min)

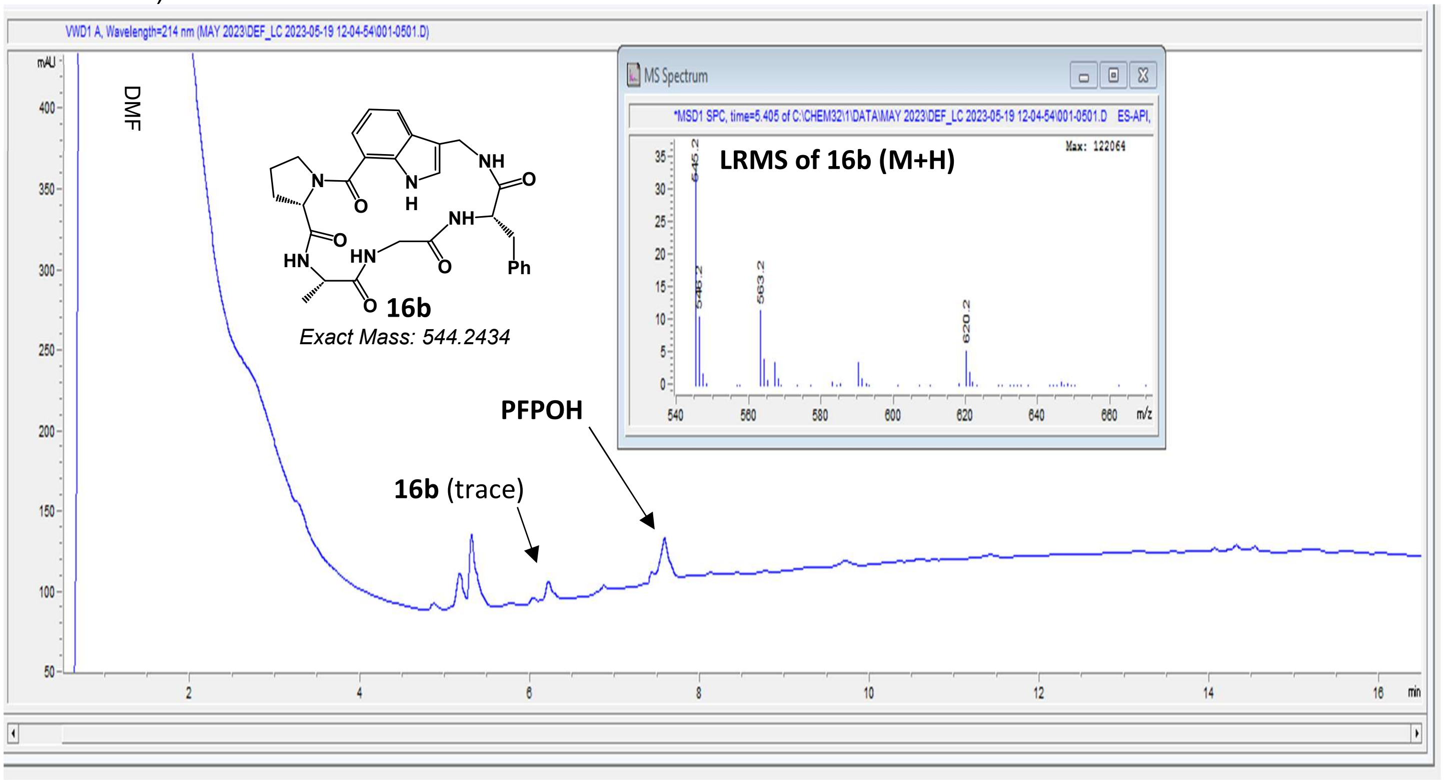

Figure S74: **Crude overnight cyclization of PFP ester - 4:** (5-95 %, MeCN/H_2O with 0.1 % formic acid over 6 min)

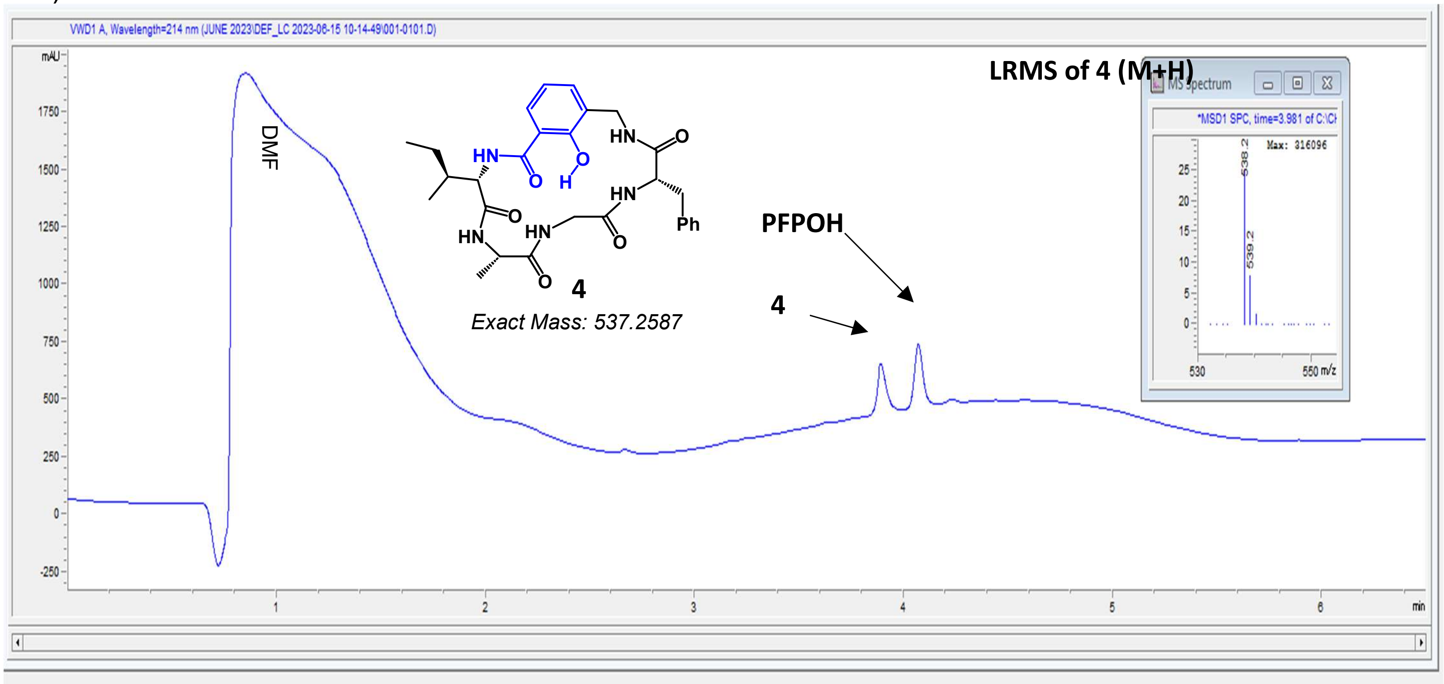

Figure S75: **Crude overnight cyclization of PFP ester - 5:** (5-95 %, MeCN/H_2O with 0.1 % formic acid over 6 min)

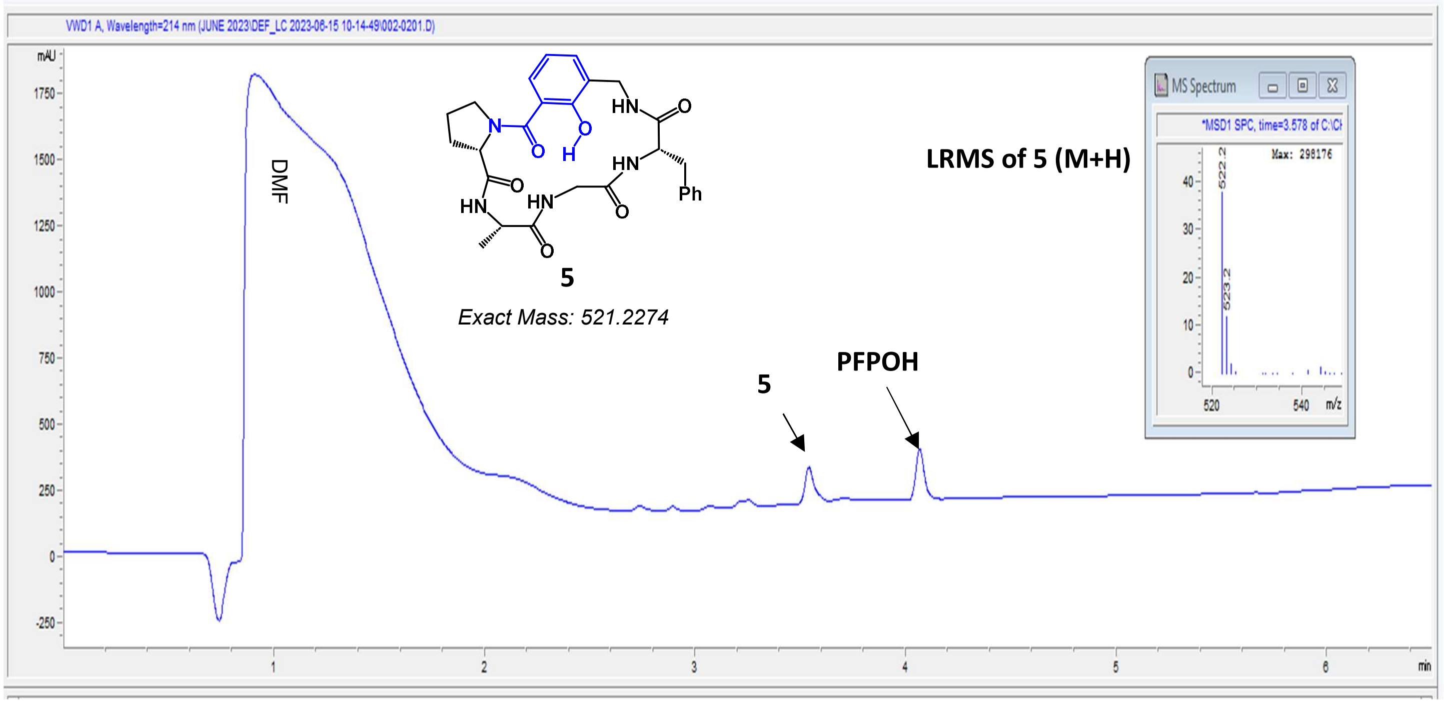

Conformational Analysis

ROE-based restraint: The NMR structures were determined by NMR derived distance information. ROESY spectra were integrated by using MestreNova (v. 10.0.2, Mestrelab Research S.L.) software. Integrated volumes of ROE crosspeaks were converted to proton interatomic distances using an inverse sixth power relationship. A reference integral was calculated as the average integral between sets of geminal protons which was then set to the calculated geminal interproton distance of 1.78 Å. The calculated distances were adjusted upwards and downwards by 10% to give upper and lower bounds to account for uncertainty in interproton distances. 3 J coupling constants were recorded from the 1H spectrum. NH-CαH 3 J coupling constants of < 6 Hz were assigned phi dihedral values of -60° +/- 25°. NHCαH 3 J coupling constants of > 8 Hz were assigned phi values of -120° +/- 25°. Crude structures of macrocycles were generated by a restrained Monte Carlo low mode molecular mechanics conformational search with an implicit solvent model (DMSO) in Macromodel (Schrodinger LLC, v11.0). The structures were then checked for violations of the experimental distances and dihedral restraints. The lowest energy structure that satisfied these tests were passed for molecular dynamics study. Molecular dynamics: Solvent explicit molecular dynamics simulations were carried out with the Desmond Molecular Dynamics software module (D.E. Shaw, v4.4) running inside Maestro (Schodinger LLC, v2015-2). The OPLS3e force field was used for parameterization of the peptidomimetic macrocycle. The macrocycle representative structure was placed in an orthorhombic box solvent box (DMSO) with a minimum distance of 12 A between solute atoms and the box boundary. The solvated box was minimized then brought to 300 K from 10 K using a restrained dynamics regime. Coulombic interactions were grouped into near- and far interactions with a near interaction cutoff of 9 A. Bonds were constrained with the SHAKE algorithm and an integration time step of 2 fs was used. The final MD production run was 100 ns in length with energy value recording every 1.2 ps and trajectory recording every 4.8 ps. The trajectory run was clustered using the Trajectory Clustering script within Maestro with a 0.4 A RMSD cutoff for variation between backbone heavy atoms and a sampling frequency of 10%. The most populated cluster (with a hydrogen bond pattern agreeing with that determined by VT-NMR) was taken as the “preferred” structure. The interproton distances were measured, compared to the experimental NMR derived distances and the violations were tabulated below:

Compound 8

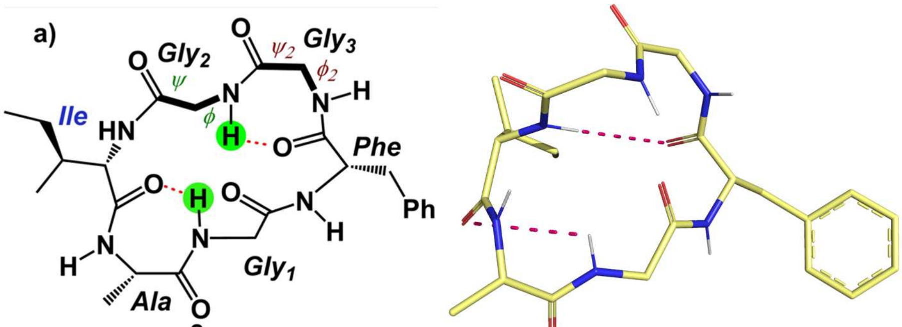

8

Residue 1	Atom 1	Residue 2	Atom 2	Calculated NOE (Å)	NOE Upper Bound (Å)	NOE Lower Bound (Å)	MD Average Distance (Å)	Violation (Å)
**Gly1**	*NH*	**Ala**	*NH*	2.46	2.66	2.26	2.4	0.00
**Gly**	*NH*	**Phe**	*NH*	2.95	3.25	2.65	3	0.09
**Gly**	*NH*	**Ala**	*αCH*	2.63	2.93	2.33	3.4	0.47
**Ile**	*NH*	**Ile**	*αCH*	2.63	2.93	2.93	2	0.43
**Gly2**	*NH*	**Gly3**	*NH*	2.71	3.01	2.41	3.9	0.89
**Gly3**	*NH*	**Phe**	*αCH*	2.27	2.47	2.07	2.9	0.43

Compound 9

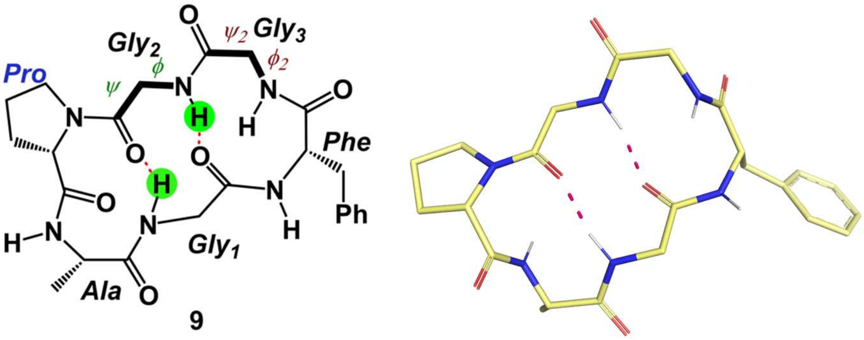

9

Residue 1	Atom 1	Residue 2	Atom 2	Calculated NOE (Å)	NOE Upper Bound (Å)	NOE Lower Bound (Å)	MD Average Distance (Å)	Violation (Å)
**Ala**	*NH*	**Gly**	*NH*	2.47	2.67	2.27	2.4	0.00
**Gly3**	*NH*	**Phe**	*αCH*	2.31	2.51	2.11	3	0.49
**Phe**	*NH*	**Phe**	*αCH*	2.32	2.52	2.12	3.2	0.68
**Gly1**	*NH*	**Ala**	*αCH*	3.42	3.72	3.12	2.2	0.92
**Ala**	*NH*	**Gly**	*NH*	2.47	2.67	2.27	2.4	0.00

Compound 10

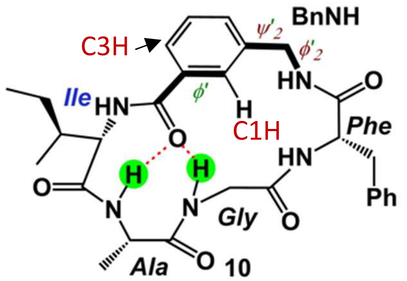

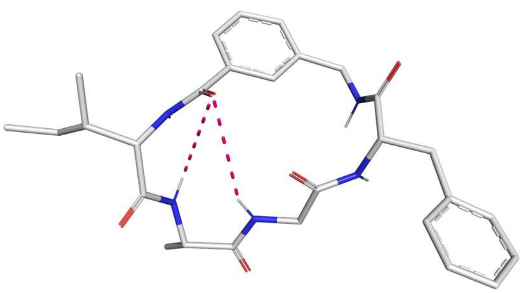

Residue 1	Atom 1	Residue 2	Atom 2	Calculated NOE (Å)	NOE Upper Bound (Å)	NOE Lower Bound (Å)	MD Average Distance (Å)	Violation (Å)
**AlaNH**	*NH*	**Gly**	*NH*	2.85	3.05	2.65	2.4	0.25
**AlaNH**	*NH*	**Ala**	αCH	2.85	3.05	2.65	3	0.00
**AlaNH**	*NH*	**Ile**	αCH	2.94	3.24	2.64	3.4	0.16
**IleNH**	*NH*	**Ala**	*NH*	2.62	2.92	2.32	2	0.32
**IleNH**	*NH*	**Aryl**	C1H	3.05	3.35	2.75	3.9	0.55
**IleNH**	*NH*	**Aryl**	C3H	2.52	2.82	2.22	2.9	0.08
**PheNH**	*NH*	**Gly**	*NH*	3.42	3.72	3.12	4.2	0.38
**PheNH**	*NH*	**Aryl**	C1H	3.42	3.72	3.12	3.6	0.00
**Gly**	*NH*	**Ala**	αCH	2.72	3.02	3.42	3	0.02
Bn	*NH*	**Aryl**	C1H	2.62	2.92	2.32	2.4	0.00

Compound 11

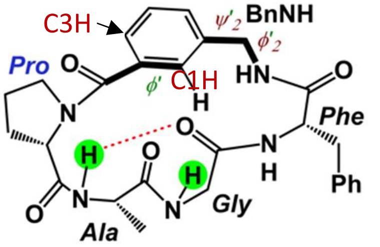

11

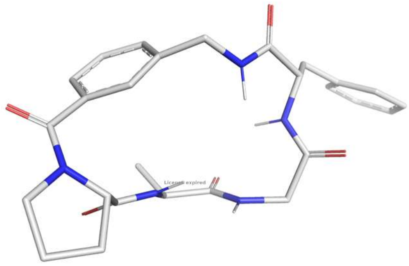

Residue 1	Atom 1	Residue 2	Atom 2	Calculated NOE (Å)	NOE Upper Bound (Å)	NOE Lower Bound (Å)	MD Average Distance (Å)	Violation (Å)
**Ala**	*NH*	**GlyNH**	*NH*	2.64	2.94	2.34	1.8	0.54
**Ala**	*NH*	**Ala**	αCH	2.59	2.89	2.29	2.8	0.00
**Bn**	*NH*	**Aryl**	C1H	2.44	2.64	2.24	3.4	0.76
**Phe**	*NH*	**Bn**	αCH	2.10	2.30	1.90	2.4	0.10
**Phe**	*NH*	**Phe**	αCH	2.50	2.80	2.20	3	0.20
**Gly**	*NH*	**Ala**	αCH	2.66	2.96	2.36	3	0.04

Compound 4

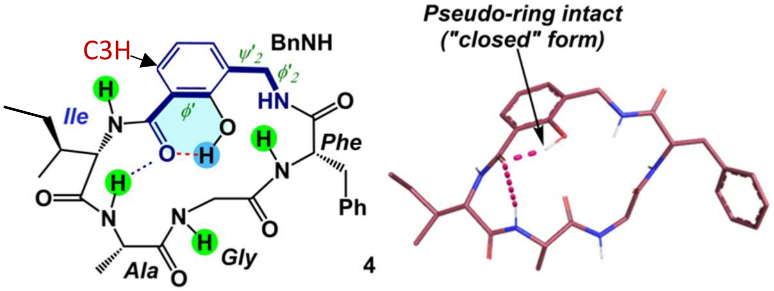

Residue 1	Atom 1	Residue 2	Atom 2	Calculated NOE (Å)	NOE Upper Bound (Å)	NOE Lower Bound (Å)	MD Average Distance (Å)	Violation (Å)
**Ala**	*NH*	**Ile**	αCH	3.19	3.89	2.66	3.6	0.00
**Ala**	*NH*	**Ala**	αCH	2.89	3.19	2.59	2.9	0.00
**Ala**	*NH*	**Ile**	*NH*	2.53	2.83	2.23	2.4	0.00
**Ile**	*NH*	**Aryl**	C3H	2.16	2.36	1.96	2	0.00
**Ile**	*NH*	**Ile**	αCH	2.93	3.23	2.63	2.9	0.00
**Aryl**	*NH*	**Phe**	*NH*	3.19	3.49	2.89	3.2	0.00
**Phe**	*NH*	**Phe**	αCH	2.93	3.23	2.63	3	0.25
**Aryl**	*NH*	**Phe**	αCH	2.34	2.54	2.14	2.7	0.16

Compound 5

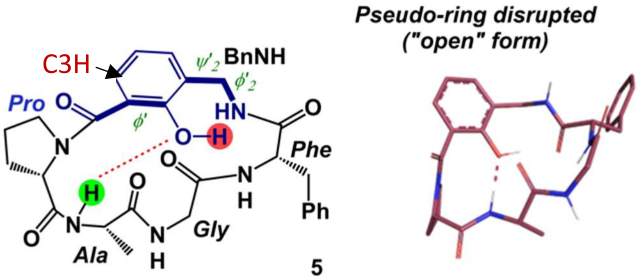

Residue 1	Atom 1	Residue 2	Atom 2	Calculated NOE (Å)	NOE Upper Bound (Å)	NOE Lower Bound (Å)	MD Average Distance (Å)	Violation (Å)
**Ala**	*NH*	**Ala**	αCH	3.29	3.59	2.99	2.9	0.09
**Bn**	*NH*	**Phe**	αCH	2.31	2.51	2.11	2.2	0.00
**Phe**	*NH*	**Phe**	αCH	3.05	3.35	2.75	2.9	0.00
**Gly**	*NH*	**Ala**	αCH	2.31	2.51	2.11	2.6	0.09

Compound 6

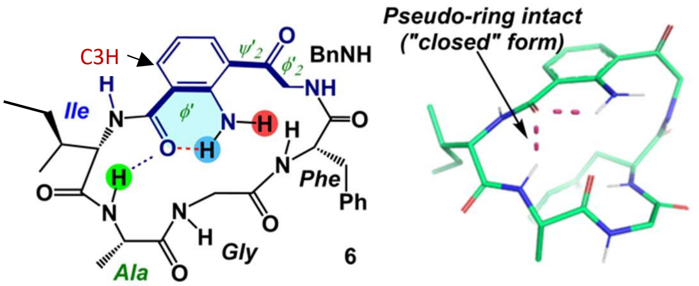

Residue 1	Atom 1	Residue 2	Atom 2	Calculated NOE (Å)	NOE Upper Bound (Å)	NOE Lower Bound (Å)	MD Average Distance (Å)	Violation (Å)
Ala	*NH*	*Gly*	*NH*	3.20	3.50	2.90	4	0.50
Ile	*NH*	*Ile*	*αCH*	3.20	3.50	2.90	2.7	0.20
Bn	*NH*	*Phe*	*αCH*	2.47	2.67	2.27	3.3	0.63
Phe	*NH*	*Phe*	*αCH*	3.05	3.35	2.75	2.8	0.00
Phe	*NH*	*Gly*	*NH*	3.42	3.72	3.12	2.9	0.22
Gly	*NH*	*Ala*	*αCH*	2.71	3.01	2.41	3.4	0.39

Compound 7

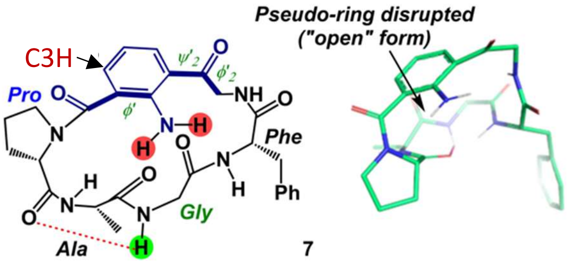

Residue 1	Atom 1	Residue 2	Atom 2	Calculated NOE (Å)	NOE Upper Bound (Å)	NOE Lower Bound (Å)	MD Average Distance (Å)	Violation (Å)
**ProCH**	*αCH*	**Aryl**	*NH2*	**2.71**	3.01	2.41	2.6	0.00
**Bn**	*NH*	**Aryl**	*αCH*	**3.19**	3.49	2.89	2.9	0.00
**Bn**	*NH*	**Phe**	*αCH*	**2.20**	2.4	2	3.4	1.00
**Phe**	*NH*	**Ala**	*αCH*	**3.04**	3.44	2.74	3.4	0.00
**Gly**	*NH*	**Ala**	*αCH*	**2.57**	2.87	2.27	2.1	0.17
**GlyNH**	*NH*	**Ala**	*NH*	**3.42**	3.72	3.12	4.3	0.58
**AlaNH**	*NH*	**Ala**	*αCH*	**2.89**	3.19	2.59	2.8	0.00
**AlaNH**	*NH*	**Pro**	*αCH*	**2.93**	3.23	2.63	3.6	0.37

Photophysical data

Theoretical section

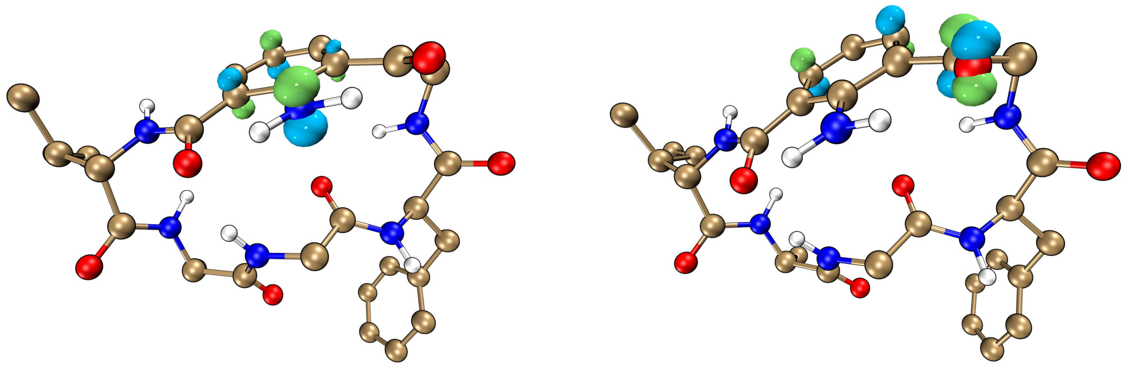

compound **6** *particle* *compound* **6** *hole*

Figure S76*:* NTO analysis to demonstrate the electronic transition (6 stands for the compound number)

The initial structures were obtained from the previous MD simulation. Geometry optimizations and vibrational frequency calculations were performed using the B3LYP-D3 functional combined with the 6-31G(d,p) basis set in the Gaussian 16.[44–49] To account for solvent effects, the IEFPCM solvent model with DMSO as the medium was applied in all computations.[50] The optimized ground-state geometries served as the starting point for vertical excitation energy simulations, conducted using time-dependent density functional theory (TD-DFT) with the same functional and basis set.[51–53] The natural transition orbital (NTO) analyses were conducted using Multiwfn, with visualization carried out in VMD, providing detailed insights into the electronic transitions.[54–56] The transition involves electron density transit from the non-bonding orbital of the aniline group to the antibonding orbital of the neighboring amide group.

Absorption and Emission measurements

Absorption spectra were measured using a Varian Cary 5000 UV–Vis-NIR spectrophotometer. Fluorescence spectra were measured using a Photon Technology International (PTI) QuantaMaster 40-F NA spectrofluorometer with a photomultiplier detector and xenon arc lamp. Cuvettes were quartz. Anthranilic acid (Ant) for quantum yield measurements was purchased from Sigma-Aldrich. For all fluorescent experiments, slit widths were set to 5 nm for both excitation and emission.

1.3 mg/mL stock: UV-Vis 1, 2, 4, 12, 18, 24 μM in DMSO of peptide **6**

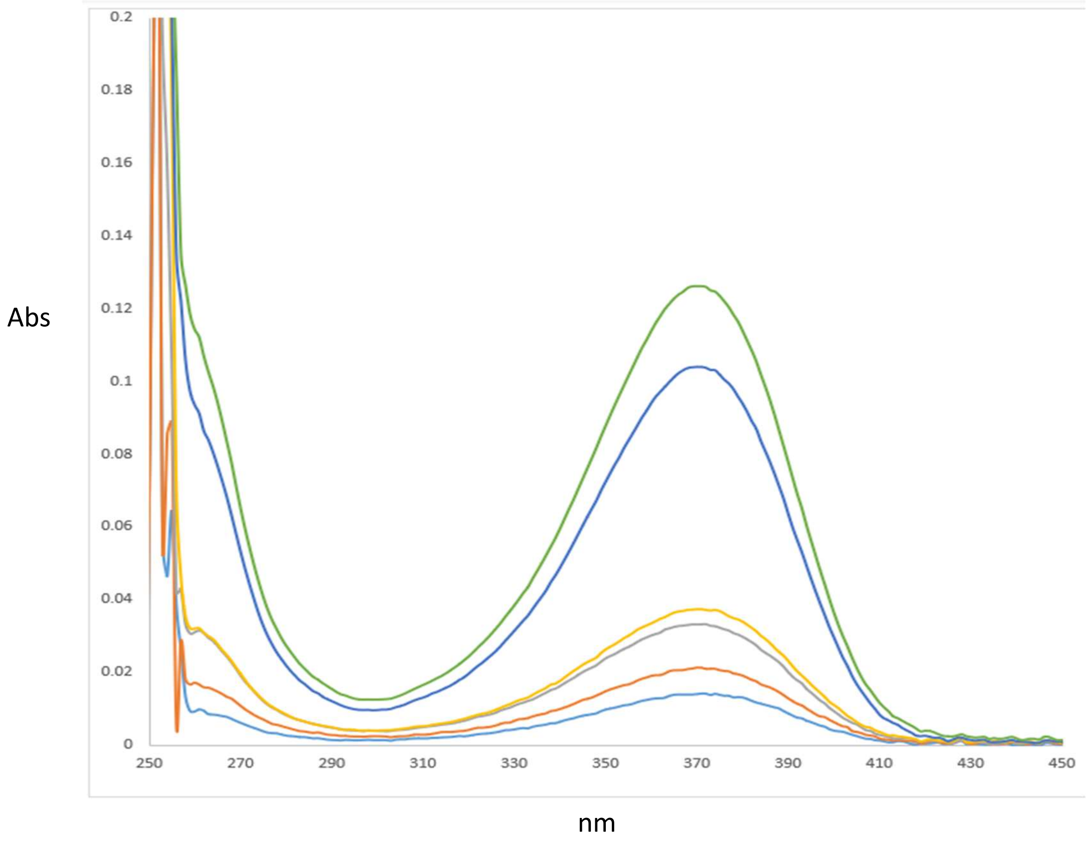

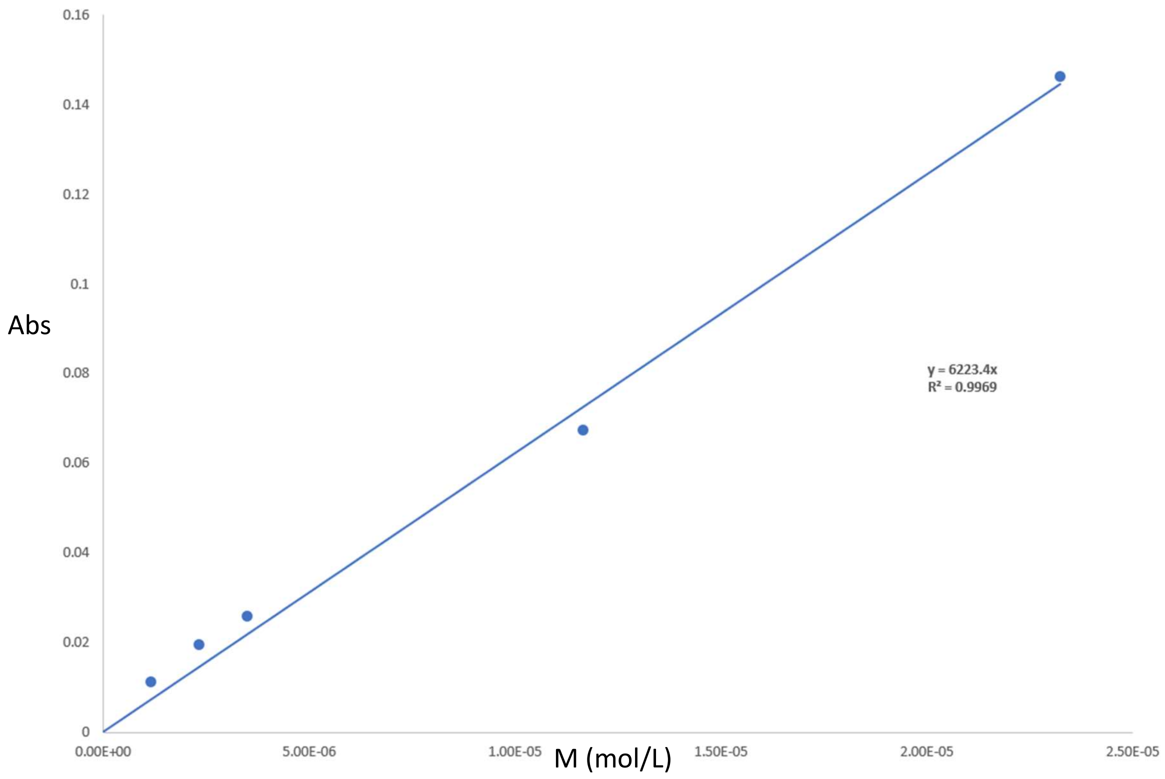
Abs
0.16
0.14
0.12
0.1
0.08
0.06
0.04
0.02
0
y = 6223.4x
R² = 0.9969
0.00E+00
5.00E-06
1.00E-05
M (mol/L)
1.50E-05
2.00E-05
2.50E-05

1.7 mg/mL stock: UV-Vis 2, 3, 15, 23, 30 μM in DMSO of peptide **7**

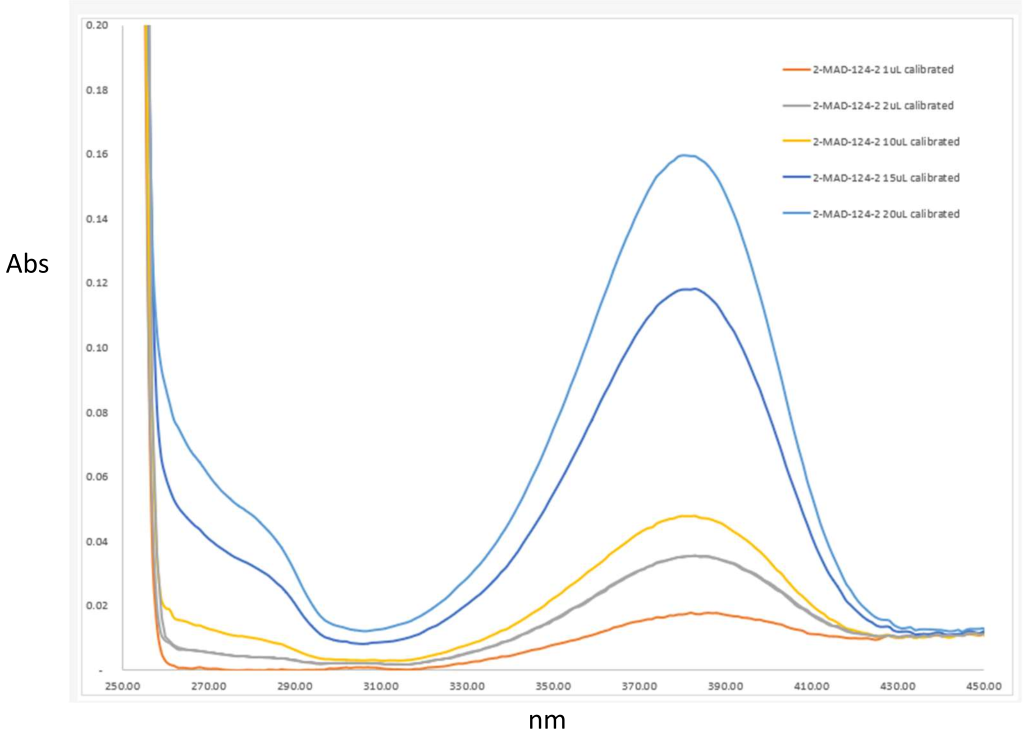

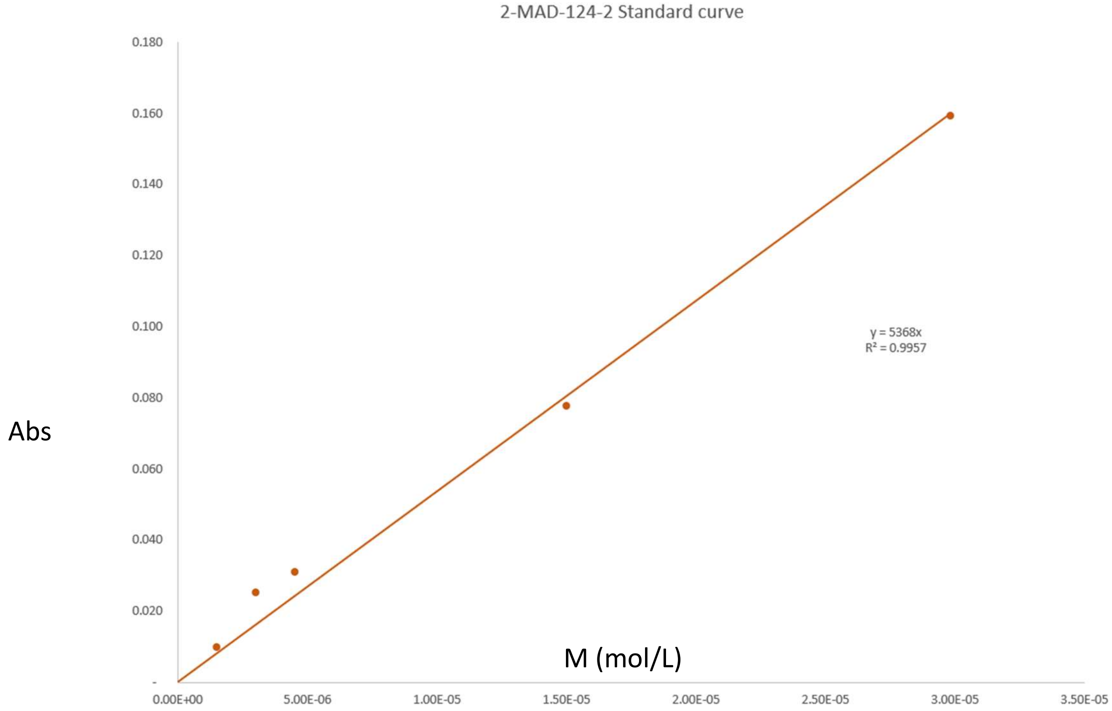

Excitation and emission spectrum at ca.10 μM in DMSO and H_2O of peptide **6**

Abs

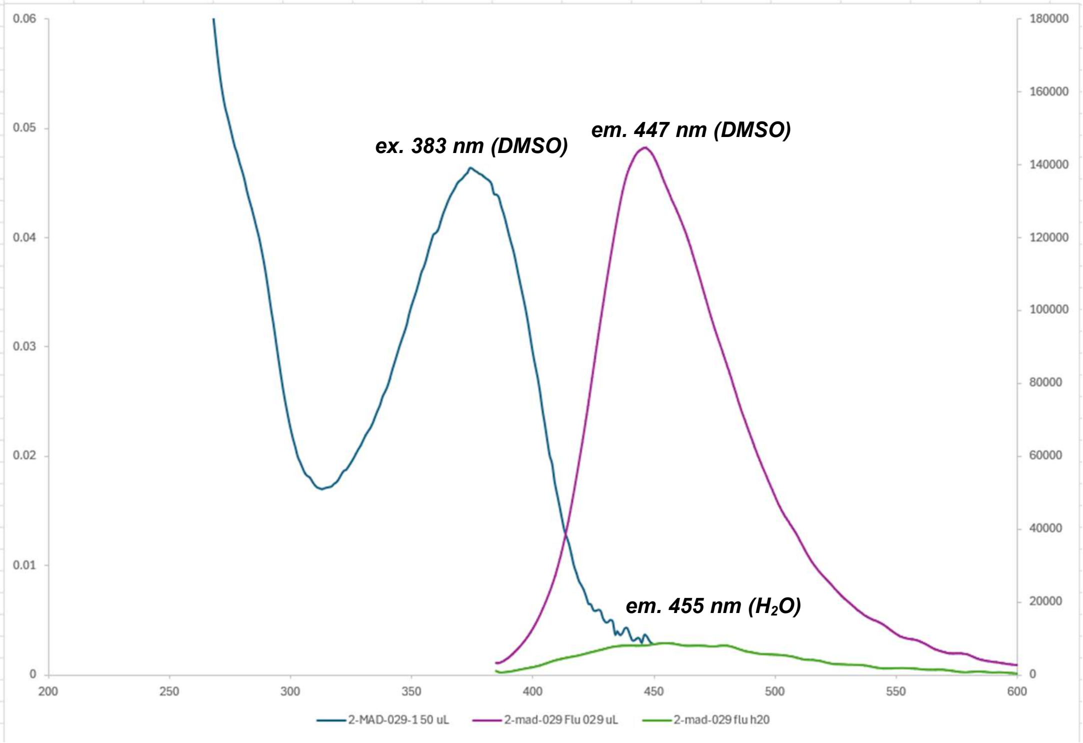

nm

Excitation and emission spectrum at ca. 10 μM in DMSO and H_2O of peptide **7**

Abs

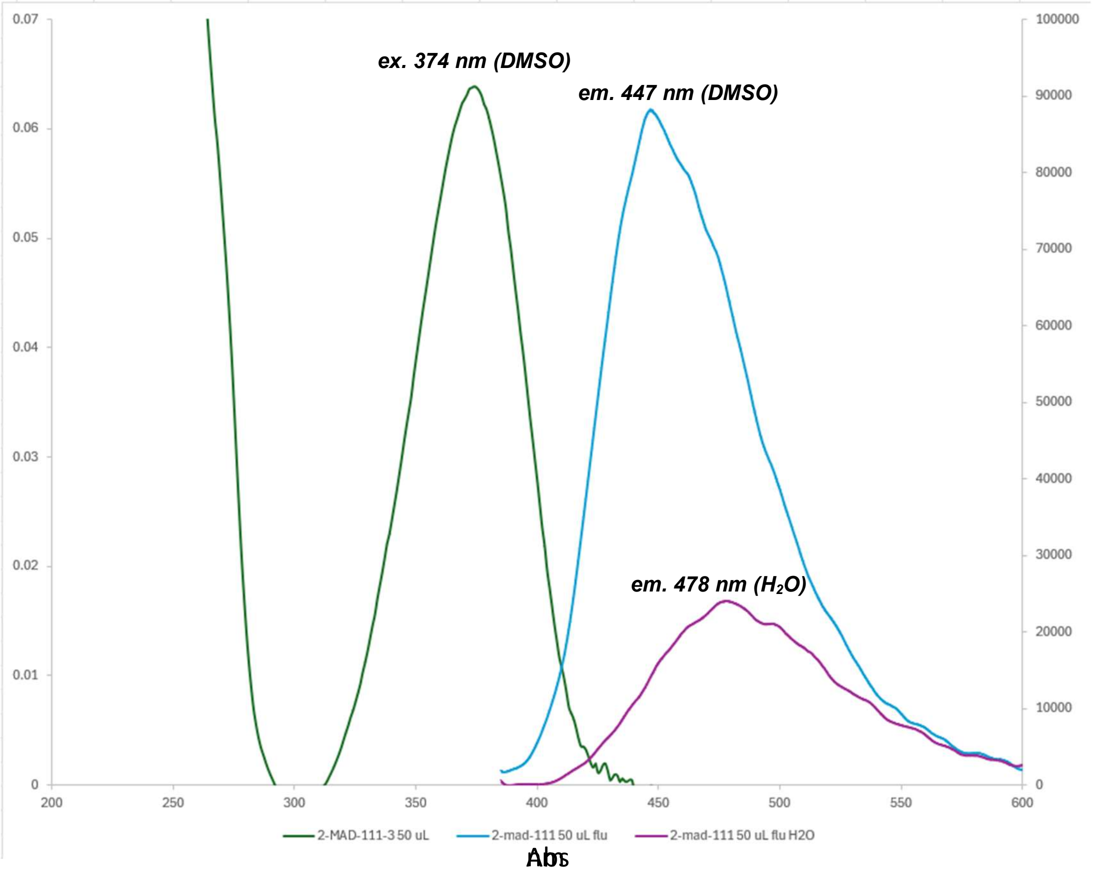

Abs nm

1.4 mg/mL stock in Ethanol: UV-vis and emission spectra at 5, 10, 25 μM in Ethanol of anthranilic acid

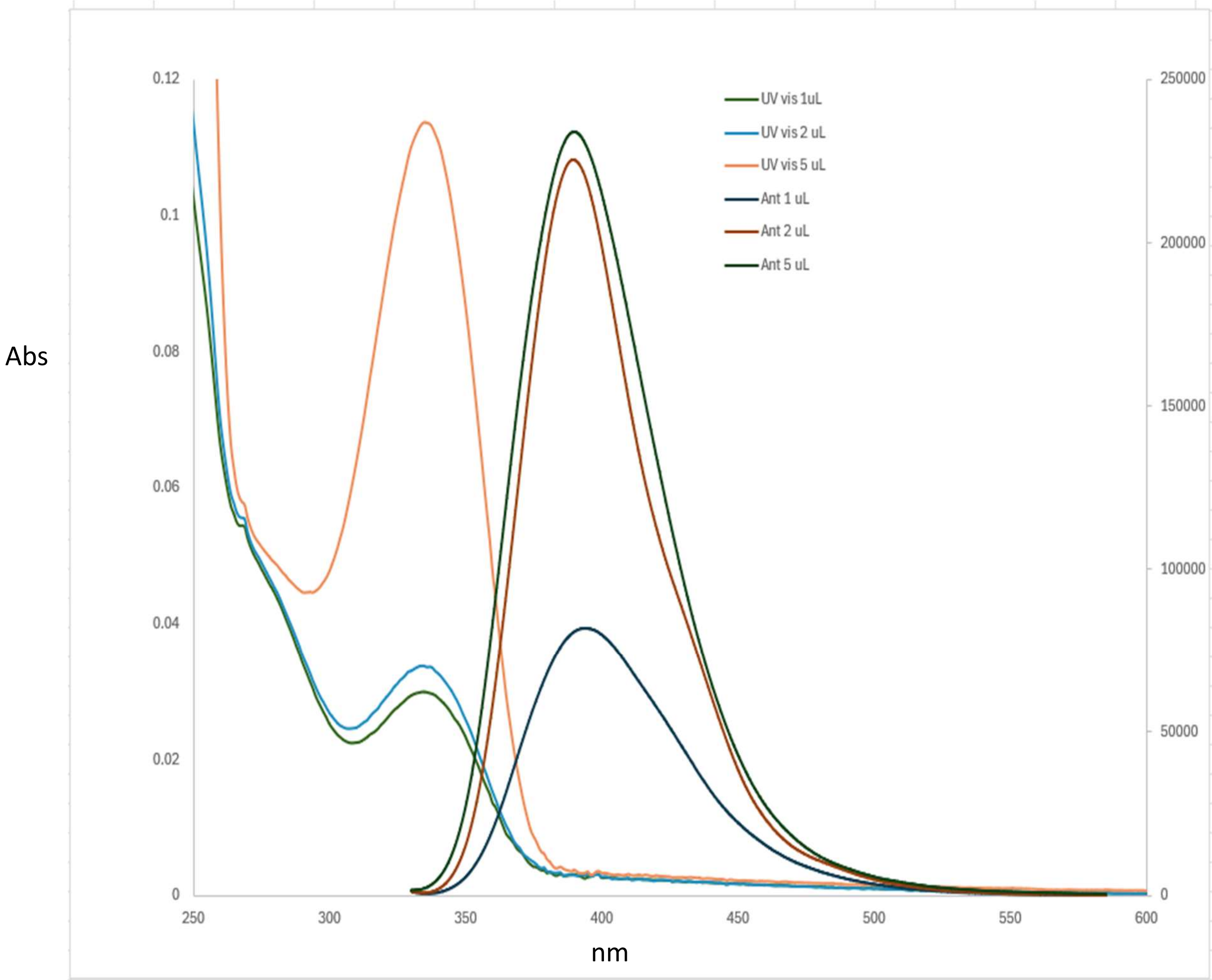

Visualization of fluorescence for peptide **6** and **7** at ca.10 μM

6 **7**

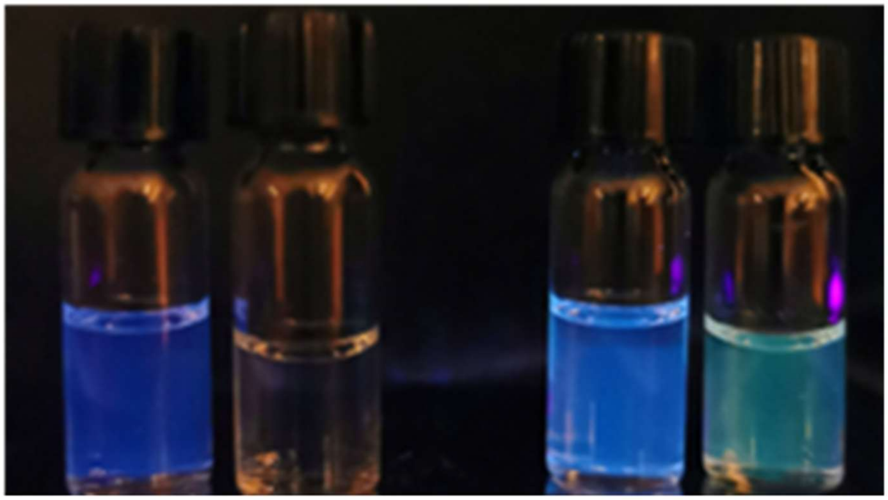

DMSO H_2O *DMSO* H_2O

Quantum yield determination

Quantum yields were determined using the following equation[57] where **Q** is the quantum yield, **I** is the integrated intensity of the emission spectra, **A** is the absorbance at the excitation wavelength, **n** is the refractive index (1.36 Ethanol, 1.48 DMSO and 1.33 H_2O), and the subscript **r** refers to the reference fluorophore (in this case Ant) with a known quantum yield (Q = 0.6 in Ethanol):

$$Q = Q_r \times \frac{I}{I_r} \times \frac{A_r}{A} \times \frac{n^2}{{n_r}^2}$$

Acknowledgements

We thank Digital Research Alliance of Canada login for computational resources. DFT computations were performed on the Cider supercomputer.

References

[39] N. Bajwa, M. P. Jennings, *J Org Chem* **2006**, *71*, 3646–3649.

[40] M. Livendahl, J. Jamroskovic, S. Ivanova, P. Demirel, N. Sabouri, E. Chorell, *Chemistry – A European Journal* **2016**, *22*, 13004–13009.

[41] H. Crassier, U. Eckert, H. Boettcher, A. Bathe, S. Emmert, *PROCESS FOR THE PREPARATION OF (3-CYANO-1H-INDOL-7-YL)(4-(4-FLUOROPHENETHYL)PIPERAZIN-1-YL)-METHANONE AND SALTS THEREOF*, **2007**, US007312342B2.

[42] B. Laleu, Y. Akao, A. Ochida, S. Duffy, L. Lucantoni, D. M. Shackleford, G. Chen, K. Katneni, F. C. K. Chiu, K. L. White, X. Chen, A. Sturm, K. J. Dechering, B. Crespo, L. M. Sanz, B. Wang, S. Wittlin, S. A. Charman, V. M. Avery, N. Cho, M. Kamaura, *J Med Chem* **2021**, *64*, 12582–12602.

[43] S. Huh, G. J. Saunders, A. K. Yudin, *Angewandte Chemie International Edition* **2022**, *n/a*, e202214729.

[44] A. D. Becke, *Phys Rev A (Coll Park)* **1988**, *38*, 3098–3100.

[45] C. Lee, W. Yang, R. G. Parr, *Phys Rev B* **1988**, *37*, 785–789.

[46] A. D. Becke, *J Chem Phys* **1993**, *98*, 5648–5652.

[47] P. C. Hariharan, J. A. Pople, *Mol Phys* **1974**, *27*, 209–214.

[48] G. A. Petersson, A. Bennett, T. G. Tensfeldt, M. A. Al-Laham, W. A. Shirley, J. Mantzaris, *J Chem Phys* **1988**, *89*, 2193–2218.

[49] S. Grimme, J. Antony, S. Ehrlich, H. Krieg, *J Chem Phys* **2010**, *132*, 154104.

[50] J. Tomasi, B. Mennucci, R. Cammi, *Chem Rev* **2005**, *105*, 2999–3094.

[51] S. Grimme, S. Ehrlich, L. Goerigk, *J Comput Chem* **2011**, *32*, 1456–1465.

[52] M. A. L. Marques, E. K. U. Gross, *Annu Rev Phys Chem* **2004**, *55*, 427–455.

[53] E. Runge, E. K. U. Gross, *Phys Rev Lett* **1984**, *52*, 997–1000.

[54] T. Lu, F. Chen, *J Comput Chem* **2012**, *33*, 580–592.

[55] W. Humphrey, A. Dalke, K. Schulten, *J Mol Graph* **1996**, *14*, 33–38.

[56] R. L. Martin, *J Chem Phys* **2003**, *118*, 4775–4777.

[57] J. Hu, C. Zhang, *Anal Chem* **2013**, *85*, 2000–2004.
